# Genetic causality and site-specific relationship between sarcopenia and osteoarthritis: a bidirectional Mendelian randomization study

**DOI:** 10.3389/fgene.2023.1340245

**Published:** 2024-01-08

**Authors:** Xue-Min Jia, Ting-Ting Deng, Hang Su, Hao-Jun Shi, Hao Qin, Gong-Chang Yu, Ying Yin, Fan-Jie Liu, Bin Shi

**Affiliations:** ^1^ Shandong First Medical University and Shandong Academy of Medical Sciences, Jinan, Shandong, China; ^2^ Neck-Shoulder and Lumbocrural Pain Hospital of Shandong First Medical University, Shandong First Medical University and Shandong Academy of Medical Sciences, Jinan, Shandong, China; ^3^ College of Traditional Chinese Medicine, Shandong University of Traditional Chinese Medicine, Jinan, China; ^4^ College of Rehabilitation, Shandong University of Traditional Chinese Medicine, Jinan, Shandong, China; ^5^ School of TCM, Macau University of Science and Technology, Macau, China; ^6^ Affiliated Hospital of Shandong University of Traditional Chinese Medicine, Jinan, Shandong, China

**Keywords:** sarcopenia, osteoarthritis, Mendelian randomization, degenerative musculoskeletal diseases, causal relationship

## Abstract

**Background:** Previous studies demonstrated a controversial relationship between sarcopenia (SP) and osteoarthritis (OA) and their genetic causality is unclear. Thus, we conducted a Mendelian randomization (MR) analysis to evaluate the possible causal association between sarcopenia-related traits (appendicular lean mass (ALM), grip strength, usual walking pace) and OA.

**Method:** We used pooled genetic data from the UK Biobank for ALM(n = 450,243), left-hand grip strength (n = 461,026), right-hand grip strength (n = 461,089) and usual walking pace (n = 459,915). Moreover, summary statistics for OA were obtained from the latest study conducted by the Genetics of Osteoarthritis Consortium, including all OA (n = 826,690), hand OA (n = 303,7782), hip OA (n = 353,388) and knee OA (n = 396,054). The primary method for estimating causal effects was the inverse-variance weighted (IVW) method, with the utilizing of false discovery rate adjusted *p* values (*P*_FDR_). Additional MR methods such as MR-Egger regression, MR pleiotropy residual sum and outlier (MR-PRESSO), weighted median were employed as supplementary analyses.

**Results:** We discovered ALM (odds ratio (OR) = 1.103, 95% confidence interval (CI) = 1.052–1.156, *P*_FDR_ = 2.87E-04), hand grip strength (left, IVW OR = 0.823, 95% CI = 0.712 to 0.952, *P*_FDR_ = 0.020; right, OR = 0.826, 95% CI = 0.718 to 0.950, *P*_FDR_ = 0.020), and usual walking pace (OR = 0.339, 95% CI = 0.204 to 0.564, *P*_*FDR*_ = 2.38E-04) were causally associated with OA risk. In the reverse MR analysis, we identified a causal effect of OA on ALM (β = −0.258, 95% CI = −0.369 to 0.146, *P*_FDR_ = 0.6.07E-06), grip strength (left, β = −0.064, 95% CI = −0.104 to 0.024, *P*_FDR_ = 0.002; right, β = −0.055, 95% CI = −0.095 to 0.014, *P*_FDR_ = 0.008), and usual walking pace (β = −0.104, 95% CI = −0.147 to 0.061, *P*_FDR_ = 1.61E-05).

**Conclusion:** This present study suggests an obvious causality of SP on OA, with condition exhibiting site-specific effects, while evidence was also provided for the causal effect of OA on SP.

## Introduction

Sarcopenia (SP) and osteoarthritis (OA) as degenerative musculoskeletal diseases (DMD) emerged as major challenges for the aging population (Yin et al., 2023). SP is a muscle disease (muscle failure) (Cruz-Jentoft et al., 2019), characterized by an accelerated loss of muscle mass and function (Cruz-Jentoft and Sayer, 2019). Currently, about 50 million people worldwide suffer from SP, and its prevalence increases with age (Hida et al., 2014). Some studies have shown that the global prevalence of SP in people over 60 years old ranges from 10.00% to 27.00%, and in people over 80 years old is as high as 50.00% (Therakomen et al., 2020; Petermann-Rocha et al., 2022). OA is a degenerative disease with clinical manifestations of chronic pain, joint stiffness, and swelling (Cho et al., 2021), which afflicts more than 500 million people worldwide and has become the leading cause of chronic pain and disability in older adults (Wen and Xiao, 2022). As SP and OA are often diagnosed as comorbidities clinically, epidemiologic studies are increasingly examining the relationship between these two prevalent diseases.

Several studies have demonstrated a significant interaction between SP and OA. SP or its related traits are likely to be associated with outcomes in OA (predominantly OA of the knee). James S Andrews et al. showed that ALM and grip strength may be related to the development of KOA in older men (Andrews et al., 2021). On the other hand, a systematic review and meta-analysis showed that the prevalence of sarcopenia was more than twice as high in patients with osteoarthritis of the knee compared with controls (Pegreffi et al., 2023). However, there are also some studies do not agree with the aforementioned notion, and they consider the interconnection of the two to be highly controversial (Jones et al., 2021; Mezian et al., 2021; Tzartza et al., 2023). In addition, for ethical and practical purposes, a causal association between the two diseases could not be proved by a randomized controlled trial (RCT).

Mendelian randomization (MR) is a data analysis technique used to evaluate etiologic inferences in epidemiologic studies. The technique utilizes genetic variation as an instrumental variable (IV) to estimate reliable causal associations between exposures and outcomes (Burgess et al., 2019; Richmond and Davey Smith, 2022). Based on a two-sample bidirectional MR framework, we examined the potential causality of all OA, hand OA, hip OA, and knee OA with SP-related traits (appendicular lean mass (ALM), hand grip strength (left), hand grip strength (right), and usual walking pace).

## Materials and methods

### Study design overview

**FIGURE 1:**
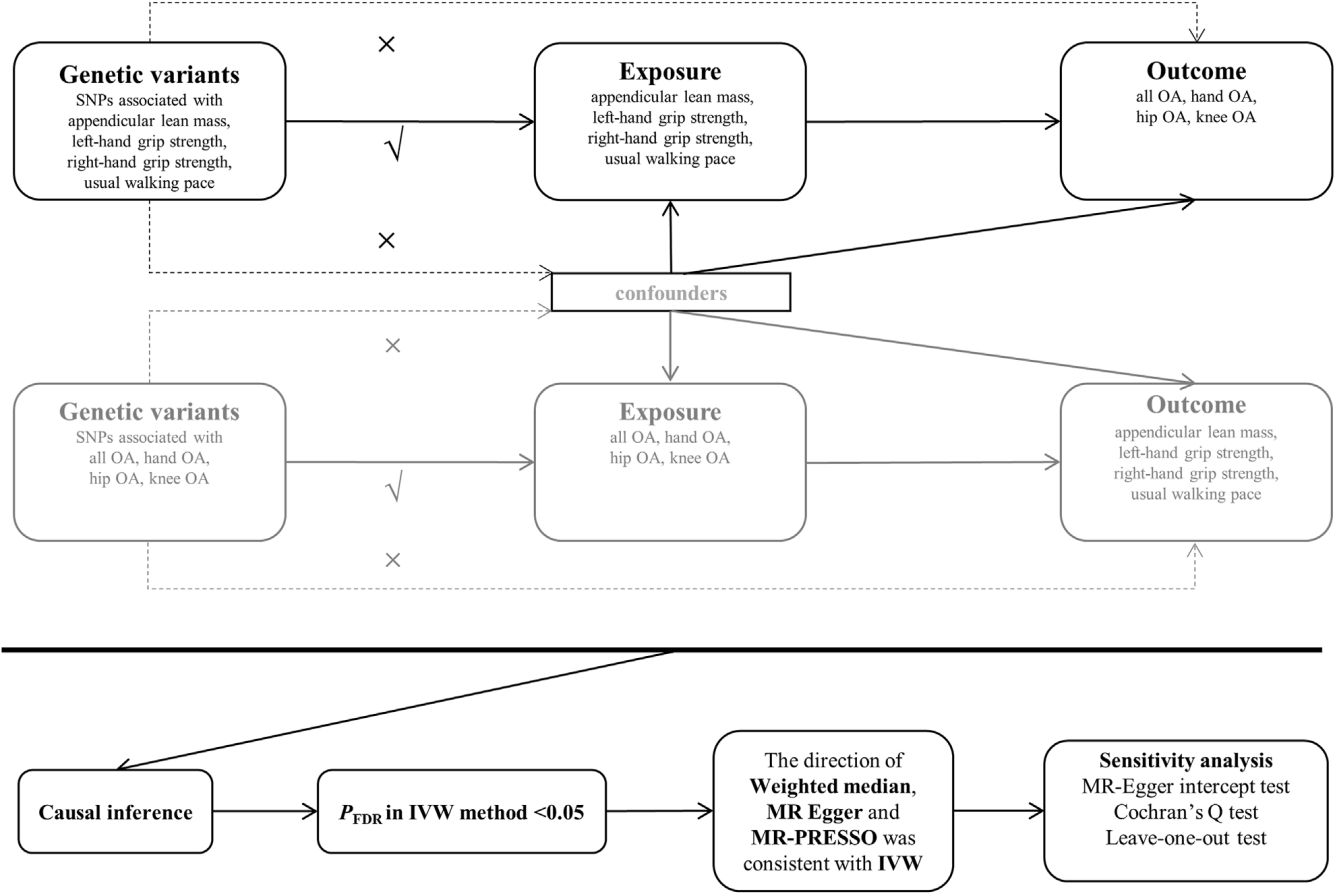
Generalized diagram of the study process. Abbreviations: OA: osteoarthritis; MR: Mendelian randomization;*P*_FDR_: The inverse-variance weighted (IVW) method with false discovery rate (FDR) adjusted *p* values.

Figure 1 illustrates the design of our bidirectional MR study. We first estimated the causal effect of SP-related traits on OA and then assessed the causal effect of OA on SP-related traits. Genetic variants were considered as IVs only if they met the following three strict core assumptions. First, genetic variants were highly correlated with exposure. Second, genetic variants are not associated with confounding factors. Finally, genetic variation cannot act directly on the outcome, its effect on the outcome can only be reflected by exposure.

### Data sources for sarcopenia-related traits

All summary-level genetic data for three SP-related traits were obtained from the UK Biobank (UKB). UKB is a large-scale repository of biomedical genes and information resources, containing about half a million people. The repository includes samples of volunteers’ genetic information, lifestyle choices, and pedigree data (Sudlow et al., 2015). Identification of ALM, hand grip strength, and usual walking pace as consensus diagnostic criteria for SP was based on the report of the European Working Group on Sarcopenia in Older People (EWGSOP) (Cruz-Jentoft et al., 2010). In terms of the ALM, pooled data were analyzed for 450,243 UKB cohort participants, and ALM-related values were quantified and adjusted (Pei et al., 2020). Grip strength data were obtained from UKB’s summary of hand grip strength (left and right) for 461,026 and 461,089 European ethnicity (Mitchell et al., 2019), calibrated to hand size, and adjusted for factors such as age and sex. Finally, for the “usual walking pace”, data was similarly summarized from the GWAS summary data collected by the UKB for 459,915 European populations (Mitchell et al., 2019).

### Data sources for all and site-specific OA

Summary statistics for all OA and its specific sites were obtained from the Genetics of Osteoarthritis (GO) Consortium’s GWAS meta-analysis involving 826,690 individuals (177,517 patients with OA and 649,173 controls) from nine different populations (Boer et al., 2021). OA was defined by the GO based on self-reported status, hospital diagnosis, the 10th edition of the International Classification of Diseases (ICD-10) code, or TREAT-OA Consortium-defined radiology definition. The study identified 100 independently associated risk variants in 11 OA phenotypes and is the most recent and comprehensive GWAS analysis known for OA. We selected four phenotypes for analysis based on research needs: all OA (n = 826,690), hand OA (n = 303,782), hip OA (n = 353,388) and knee OA (n = 396,054).

Table 1 shows additional details such as phenotypes of all study participants.

**TABLE 1:** Data sources used in this study.

Phenotype	Sample size (cases/controls)	Population	GWAS ID or PMID
ALM	450,243	European	ebi-a-GCST90000025
hand grip strength (left)	461,026	European	ukb-b-7478
hand grip strength (right)	461,089	European	ukb-b-10215
usual walking pace	459,915	European	ukb-b-4711
All OA	826,690 (177,517/649,173)	Mixed	34,822,786
Hand OA	303,782 (20,901/282,881)	Mixed	34,822,786
Hip OA	353,388 (36,445/316,943)	Mixed	34,822,786
Knee OA	396,054 (62,497/333,557)	Mixed	34,822,786

Abbreviations: ALM: appendicular lean mass; OA, osteoarthritis.

### Selection of IVs

We first ensured that the SNPs of the three SP-related traits were genome-wide significant (*p* < 5E-08). Furthermore, to exclude the SNPs of strong linkage disequilibrium, we carried out an aggregation with *r* ^2^ < 0.001 and kb = 10,000 processes. Then, we also assessed the strength of IVs based on calculated *R* ^2^ [*R* ^2^ = 2 * MAF*(1-MAF) *beta (Cruz-Jentoft et al., 2019)] and F-statistics [F = R (Cruz-Jentoft et al., 2019)/(1-R^2^) * (N-K-1)/K] for each SNP (Burgess et al., 2011). In order to reduce bias, the IVs with F-statistics less than 10 were excluded (Hemani et al., 2018a), and the SNPs and their related data such as F-statistics and *R* ^2^ for subsequent causal analysis are listed in Supplementary Table S1. In the reverse MR analysis, the SNP screening process was consistent with the aforementioned procedure.

### MR analysis

This study used R software (Version 4.3.0) and the two-sample MR package (Version 0.5.7) for data analysis (Yavorska and Burgess, 2017). We used the random-effects IVW method as the primary analytical method for MR estimation. This method was considered the most reliable in the absence of indications of directional pleiotropy in the selected IVs (Holmes et al., 2017). In addition, several sensitivity analyses were carried out, including weighted median (WM), MR-Egger, MR pleiotropy residual sum, and outlier (MR-PRESSO) test. Within this framework, the WM selected the median estimate to calculate the causal effects (Burgess et al., 2017). The MR-Egger regression method effectively tests the null causality hypothesis and gives consistent estimates of causality even if no genetic variation is valid. Additionally, the MR-Egger regression method is robust to horizontal pleiotropy (Bowden et al., 2015). The MR-PRESSO method detects pleiotropy, removes potentially pleiotropic IVs (outliers), and provides outlier-adjusted estimates (Verbanck et al., 2018). In order to correct the problem of multiple testing relatively gently, the *p*-value was adjusted using the false discovery rate (FDR) which is also called *q* value in the main IVW MR analysis, and the significance of causal inference was set to less than 0.05 (Chen et al., 2021). In addition, the statistical power was calculated with an online sample size and power calculator for MR (https://sb452.shinyapps.io/power/), and the results are shown in Supplementary Table S4.

Heterogeneity was assessed using Cochran’s Q test, and *p* < 0.05 was considered statistically significant (Bowden et al., 2016). Moreover, we performed pleiotropic tests using MR-Egger intercept test and MR-PRESSO global testing to ensure that IVs do not influence the risk of outcome through other confounding factors or other biological pathways unrelated to exposure (Hemani et al., 2018b). Furthermore, we performed leave-one-out analyses to ensure the reliability of associations with individual SNPs.

## Results

### Overview

Based on the inverse-variance weighted (IVW) method, we observed significant evidence of a bi-directionally causal relationship between SP and OA. After FDR correction, most of the meaningful results were still retained. Additionally, the results were corroborated using other MR analysis methods.

### The causal effects of SP-related traits on OA

After accounting for the independence of the genetic variation, none of the IVs for ALM, hand grip strength (left), hand grip strength (right), and usual walking pace that we obtained were without linkage disequilibrium (kb > 10,000 and *r* ^2^ < 0.001). Furthermore, all IVs reached genome-wide significance (*p* < 5E-08). Additionally, IVs with an F-statistic of less than 10 were considered weak instruments and were omitted from the MR analysis thus reducing the bias in the estimation of the IVs. Ultimately, we selected 583 SNPs, 127 SNPs, 144 SNPs, and 47 SNPs as IVs for ALM, hand grip strength (left), hand grip strength (right), and usual walking pace, respectively. Details of the IVs for SP-related traits are displayed in Supplementary Tables S1, S2.

As displayed in Table 2, a portion of Cochran’s Q test was used to detect heterogeneity (*p* < 0.05), hence we used the IVW method in the random effects model. In the IVW model, adjusted for the FDR, genetically elevated SP-related traits levels were causally associated with all OA, ALM (Odds ratio (OR) = 1.103, 95% confidence interval (CI) = 1.052–1.156, *P*_FDR_ = 2.87E-04), hand grip strength (left, OR = 0.823, 95% CI = 0.712 to 0.952, *P*_FDR_ = 0.020; right, OR = 0.826, 95% CI = 0.718 to 0.950, *P*_FDR_ = 0.020), and usual walking pace (OR = 0.339, 95% CI = 0.204 to 0.564, *P*_*FDR*_ = 2.38E-04). The results of the WM and MR-PRESSO were consistent with the IVW (Table 3). No evidence of directional pleiotropy in the MR-Egger intercept test was observed for all IVs (*p* for intercept >0.05) (Table 2). Leave-one-out test indicated that SNPs without large effect sizes skewed the estimates (Supplementary Figure S1).

**TABLE 2:** Sensitivity and IVW analysis of the causal effects of sarcopenia-related traits on osteoarthritis.

Exposures	Outcomes	No. of IVs	Heterogeneity test	MR Egger		IVW		
			Cochran’s Q (*P*)	Intercept	*P*	OR (95% CI)	*P*	*P*_FDR_
**Appendicular lean mass**	**All OA**	188	314.35 (<0.001)	0.002	0.160	1.103 (1.052,1.156)	5.38E-05	2.87E-04
**Hand grip strength (left)**	**All OA**	40	32.320 (0.767)	−0.001	0.827	0.823 (0.712,0.952)	0.009	0.020
**Hand grip strength (right)**	**All OA**	44	32.080 (0.889)	−0.001	0.706	0.826 (0.718,0.950)	0.007	0.020
**Usual walking pace**	**All OA**	18	63.293 (<0.001)	0.008	0.666	0.339 (0.204,0.564)	2.98E-05	2.38E-04
**Appendicular lean mass**	**Hand OA**	196	229.148 (0.047)	−4.86E-04	0.852	1.123 (1.014,1.243)	0.026	0.052
**Hand grip strength (left)**	**Hand OA**	52	84.381 (0.002)	−0.013	0.213	0.817 (0.535,1.250)	0.352	0.402
**Hand grip strength (right)**	**Hand OA**	59	63.282 (0.295)	−0.008	0.326	0.768 (0.550,1.073)	0.122	0.195
**Usual walking pace**	**Hand OA**	18	28.491 (0.040)	0.015	0.663	1.026 (0.412,2.556)	0.956	0.956
**Appendicular lean mass**	**Hip OA**	194	320.495 (<0.001)	0.004	0.070	1.095 (1.001,1.197)	0.048	0.085
**Hand grip strength (left)**	**Hip OA**	44	31.552 (0.902)	0.003	0.608	1.159 (0.885,1.518)	0.282	0.348
**Hand grip strength (right)**	**Hip OA**	50	37.682 (0.880)	0.001	0.847	1.194 (0.930,1.534)	0.165	0.240
**Usual walking pace**	**Hip OA**	16	13.983 (0.527)	0.013	0.546	0.474 (0.272,0.827)	0.008	0.020
**Appendicular lean mass**	**Knee OA**	205	472.488 (<0.001)	0.001	0.606	1.246 (1.151,1.350)	6.07E-08	9.71E-07
**Hand grip strength (left)**	**Knee OA**	50	94.915 (<0.001)	−0.013	0.051	1.182 (0.902,1.548)	0.226	0.301
**Hand grip strength (right)**	**Knee OA**	59	135.377 (<0.001)	−0.004	0.530	0.991 (0.753,1.305)	0.950	0.956
**Usual walking pace**	**Knee OA**	18	63.585 (<0.001)	0.010	0.744	0.299 (0.136,0.659)	0.003	0.011

Abbreviations: MR, mendelian randomization; IVW, inverse variance weighted; IVs, instrumental variables; *P*_FDR_, false discovery rate (FDR) adjusted *p* values; CI, confidence interval; OA, osteoarthritis.

**TABLE 3:** Weighted median, MR Egger and MR PRESSO analysis of the causal effects of sarcopenia-related traits on osteoarthritis.

Exposures	Outcomes	No. of IVs	Weighted median		MR Egger		MR PRESSO	
			OR (95% CI)	*P*	OR (95% CI)	*P*	OR (95% CI)	*P*
**Appendicular lean mass**	**All OA**	188	1.096 (1.029,1.168)	0.004	1.025 (0.916,1.147)	0.670	1.098 (1.095,1.102)	1.08E-04
**Hand grip strength (left)**	**All OA**	40	0.896 (0.731,1.098)	0.290	0.888 (0.446,1.769)	0.737	0.823 (0.806,0.841)	0.006
**Hand grip strength (right)**	**All OA**	44	0.924 (0.754,1.131)	0.442	0.928 (0.500,1.722)	0.814	0.826 (0.811,0.841)	0.003
**Usual walking pace**	**All OA**	18	0.385 (0.236,0.629)	1.40E-04	0.131 (0.002,9.437)	0.366	0.296 (0.268,0.326)	4.85E-05
**Appendicular lean mass**	**Hand OA**	196	1.138 (0.984,1.315)	0.081	1.146 (0.906,1.448)	0.257	1.123 (1.115,1.131)	0.027
**Hand grip strength (left)**	**Hand OA**	52	0.934 (0.551,1.582)	0.799	2.540 (0.415,15.543)	0.318	0.817 (0.771,0.867)	0.356
**Hand grip strength (right)**	**Hand OA**	59	0.697 (0.439,1.107)	0.126	1.496 (0.384,5.822)	0.564	0.768 (0.736,0.802)	0.127
**Usual walking pace**	**Hand OA**	18	0.774 (0.272,2.202)	0.631	0.180 (0.001,416.109)	0.670	0.788 (0.658,0.945)	0.552
**Appendicular lean mass**	**Hip OA**	194	1.048 (0.932,1.179)	0.435	0.921 (0.750,1.132)	0.435	1.095 (1.088,1.102)	0.049
**Hand grip strength (left)**	**Hip OA**	44	1.185 (0.805,1.746)	0.390	0.863 (0.273,2.727)	0.803	1.159 (1.12,1.2)	0.216
**Hand grip strength (right)**	**Hip OA**	50	1.241 (0.865,1.78)	0.241	1.079 (0.379,3.076)	0.887	1.194 (1.157,1.232)	0.12
**Usual walking pace**	**Hip OA**	16	0.538 (0.252,1.147)	0.109	0.105 (0.001,12.883)	0.374	0.474 (0.415,0.542)	0.016
**Appendicular lean mass**	**Knee OA**	205	1.271 (1.155,1.399)	9.32E-07	1.194 (0.995,1.432)	0.057	1.256 (1.25,1.263)	1.04E-08
**Hand grip strength (left)**	**Knee OA**	50	0.964 (0.697,1.334)	0.824	3.382 (1.174,9.742)	0.029	1.124 (1.083,1.167)	0.385
**Hand grip strength (right)**	**Knee OA**	59	0.985 (0.744,1.305)	0.919	1.387 (0.472,4.078)	0.555	1.057 (1.022,1.093)	0.675
**Usual walking pace**	**Knee OA**	18	0.422 (0.204,0.871)	0.020	0.098 (0.001,74.976)	0.502	0.357 (0.306,0.416)	0.008

Abbreviations: MR, mendelian randomization; IVW, inverse variance weighted; IVs, instrumental variables; CI, confidence interval; OA, osteoarthritis.

In the site-specific OA analysis, considering the results of the heterogeneity test, the IVW in the random effects model was used. In the IVW model, we observed that genetically determined levels of ALM were causally associated with knee OA (OR = 1.246, 95% CI = 1.151 to 1.350, *P*_*FDR*_ = 9.71E-07), usual walking pace was negatively correlated with hip OA (OR = 0.474, 95% CI = 0.272 to 0.827, *P*_*FDR*_ = 0.020) and knee OA (OR = 0.299, 95% CI = 0.136 to 0.659, *P*_*FDR*_ = 0.011) (Table 2). Moreover, WM and MR-PRESSO reached similar causal conclusions, and the MR-Egger effect estimate was in the same direction as IVW, which was considered supportive (Table 3). Further, no directional pleiotropy was detected for our selected IVs on MR-Egger analysis (Table 2). Leave-one-out test results were consistent with the above (Supplementary Figures S2–S4).

### The causal effect of OA on SP-related traits

After similar screening criteria, 25 SNPs, 8 SNPs, 28 SNPs, and 22 SNPs were obtained as IVs for all OA, hand OA, hip OA, and knee OA, respectively (Supplementary Tables S1, S3). As Table 4 illustrates, heterogeneity was observed (*p* < 0.05) between the IVs of all the selected OA and the traits associated with SP. Therefore, we used the random effects model of IVW. The results of our analysis showed that the onset and progression of OA could lead to the worsening of sarcopenia-related traits [ALM (IVW β = −0.258, 95% CI = −0.369 to −0.146, *P*_FDR_ = 0.6.07E-06), grip strength (left, β = −0.064, 95% CI = −0.104 to −0.024, *P*_FDR_ = 0.002; right, β = −0.055, 95% CI = −0.095 to −0.014, *P*_*FDR*_ = 0.008), and usual walking pace (β = −0.104, 95% CI = −0.147 to −0.061, *P*_FDR_ = 1.61E-05)]. The WM and MR-PRESSO suggested similar findings, and the beta values of the MR-Egger are in the same direction (Table 5). MR-Egger intercept test did not demonstrate directional pleiotropy for our selected IVs (Table 4). The results of the leave-one-out- test displayed that SNPs without large effect sizes resulted in biases in the estimates (Supplementary Figure S5).

**TABLE 4:** Sensitivity and IVW analysis of the causal effects of osteoarthritis on sarcopenia-related traits.

Exposures	Outcomes	No. of IVs	Heterogeneity test	MR Egger		IVW		
			Cochran’s Q (*P*)	Intercept	*P*	Beta (95% CI)	*P*	*P*_FDR_
**All OA**	**Appendicular lean mass**	11	72.193 (<0.001)	−0.002	0.891	−0.258 (-0.369, −0.146)	6.07E-06	3.24E-05
**All OA**	**Hand grip strength (left)**	20	53.312 (<0.001)	−0.002	0.676	−0.064 (-0.104, −0.024)	0.002	0.004
**All OA**	**Hand grip strength (right)**	19	48.792 (<0.001)	−0.001	0.758	−0.055 (-0.095, −0.014)	0.008	0.014
**All OA**	**Usual walking pace**	22	104.602 (<0.001)	−0.002	0.673	−0.104 (-0.147, −0.061)	2.01E-06	1.61E-05
**Hand OA**	**Appendicular lean mass**	7	186.506 (<0.001)	0.010	0.919	0.013 (-0.095,0.122)	0.81	0.810
**Hand OA**	**Hand grip strength (left)**	8	84.682 (<0.001)	−0.007	0.684	−0.102 (-0.149, −0.055)	2.26E-05	7.23E-05
**Hand OA**	**Hand grip strength (right)**	8	86.489 (<0.001)	−0.005	0.759	−0.104 (-0.152, −0.057)	1.84E-05	7.23E-05
**Hand OA**	**Usual walking pace**	8	26.509 (<0.001)	−0.001	0.937	−0.021 (-0.044,0.001)	0.062	0.100
**Hip OA**	**Appendicular lean mass**	22	366.557 (<0.001)	−0.004	0.654	0.047 (-0.006,0.100)	0.08	0.117
**Hip OA**	**Hand grip strength (left)**	28	266.255 (<0.001)	0.003	0.564	0.013 (-0.015,0.041)	0.359	0.414
**Hip OA**	**Hand grip strength (right)**	28	309.543 (<0.001)	0.003	0.566	0.011 (-0.019,0.041)	0.474	0.506
**Hip OA**	**Usual walking pace**	22	45.030 (0.002)	0.001	0.666	−0.025 (-0.038, −0.013)	9.81E-05	2.62E-04
**Knee OA**	**Appendicular lean mass**	15	316.000 (<0.001)	0.013	0.51	−0.179 (-0.281, −0.076)	0.001	0.001
**Knee OA**	**Hand grip strength (left)**	19	309.382 (<0.001)	0.003	0.841	0.029 (-0.092,0.034)	0.362	0.414
**Knee OA**	**Hand grip strength (right)**	19	342.572 (<0.001)	0.003	0.825	0.041 (-0.107,0.025)	0.228	0.304
**Knee OA**	**Usual walking pace**	20	70.118 (<0.001)	0.007	0.066	−0.064 (-0.087, −0.04)	8.55E-08	1.37E-06

Abbreviations: MR, mendelian randomization; IVW, inverse variance weighted; IVs, instrumental variables; *P*_FDR_, false discovery rate (FDR) adjusted *p* values; CI, confidence interval; OA, osteoarthritis.

**TABLE 5:** Weighted median, MR Egger and MR PRESSO analysis of the causal effects of osteoarthritis on sarcopenia-related traits.

Exposures	Outcomes	No. of IVs	Weighted median		MR Egger		MR-PRESSO	
			Beta (95% CI)	*P*	Beta (95% CI)	*P*	Beta (95% CI)	*P*
**All OA**	**Appendicular lean mass**	11	−0.168 (-0.245, −0.092)	1.66E-05	−0.193 (-1.104,0.719)	0.689	−0.218 (-0.245, −0.191)	0.002
**All OA**	**Hand grip strength (left)**	20	−0.085 (-0.124, −0.046)	1.71E-05	−0.001 (-0.292,0.290)	0.993	−0.064 (-0.073, −0.055)	0.006
**All OA**	**Hand grip strength (right)**	19	−0.053 (-0.095, −0.012)	0.012	−0.009 (-0.297,0.278)	0.949	−0.055 (-0.064, −0.046)	0.016
**All OA**	**Usual walking pace**	22	−0.112 (-0.148, −0.076)	1.56E-09	−0.027 (-0.382,0.328)	0.884	−0.102 (-0.109, −0.094)	2.09E-05
**Hand OA**	**Appendicular lean mass**	7	0.014 (-0.019,0.047)	0.403	−0.112 (−2.417,2.192)	0.928	0.01 (0.003,0.017)	0.411
**Hand OA**	**Hand grip strength (left)**	8	−0.088 (-0.114, −0.062)	3.46E-11	−0.014 (-0.420,0.391)	0.948	−0.09 (-0.094, −0.085)	0.001
**Hand OA**	**Hand grip strength (right)**	8	−0.087 (-0.113, −0.061)	5.04E-11	−0.037 (-0.450,0.376)	0.866	−0.088 (-0.097, −0.079)	0.002
**Hand OA**	**Usual walking pace**	8	−0.018 (-0.034, −0.001)	0.032	−0.013 (−0.209,0.183)	0.899	−0.021 (-0.024, −0.018)	0.006
**Hip OA**	**Appendicular lean mass**	22	0.03 (0.002,0.059)	0.036	0.100 (−0.134,0.333)	0.413	0.029 (0.024,0.033)	0.023
**Hip OA**	**Hand grip strength (left)**	28	0.02 (0.004,0.036)	0.015	−0.026 (−0.159,0.107)	0.706	0.015 (0.012,0.017)	0.035
**Hip OA**	**Hand grip strength (right)**	28	0.016 (-0.001,0.032)	0.061	−0.031 (−0.175,0.113)	0.678	0.012 (0.009,0.016)	0.176
**Hip OA**	**Usual walking pace**	22	−0.023 (-0.038, −0.009)	0.001	−0.039 (−0.104,0.026)	0.248	−0.025 (-0.028, −0.022)	0.001
**Knee OA**	**Appendicular lean mass**	15	−0.083 (-0.139, −0.027)	0.004	−0.439 (−1.198,0.321)	0.278	−0.182 (-0.199, −0.165)	0.001
**Knee OA**	**Hand grip strength (left)**	19	0.001 (-0.036,0.039)	0.945	−0.090 (−0.679,0.499)	0.768	−0.032 (-0.041, −0.023)	0.127
**Knee OA**	**Hand grip strength (right)**	19	−0.039 (-0.075, −0.003)	0.035	−0.111 (−0.731,0.509)	0.729	−0.051 (-0.06, −0.042)	0.031
**Knee OA**	**Usual walking pace**	20	−0.048 (-0.07, −0.027)	7.94E-06	−0.202 (−0.343, −0.062)	0.011	−0.06 (-0.064, −0.056)	4.83E-06

Abbreviations: MR, mendelian randomization; IVW, inverse variance weighted; IVs, instrumental variables; CI, confidence interval; OA, osteoarthritis.

In the site-specific analysis, the results of using the IVW method in the random effects model pointed to a negative causal relationship between hand OA and grip strength (left, β = −0.102, 95% CI = −0.149 to −0.055, *P*_FDR_ = 7.23E-05; right, β = -0.104, 95% CI = −0.152 to −0.057, *P*_FDR_ = 7.23E-05). Hip OA (β = −0.025, 95% CI = −0.038 to −0.013, *P*_FDR_ = 2.62E-04) and knee OA (β = −0.064, 95% CI = −0.087 to −0.04, *P*_FDR_ = 1.37E-06), remained significantly causally and negatively correlated with usual walking pace (Table 4). In addition, we identified that only knee OA was causally related to ALM in the available site-specific cohorts (β = −0.179, 95% CI = −0.281 to −0.076, *P*_FDR_ = 0.001), the results of all other MR analysis methods were similar to those of the IVW (Table 5). Heterogeneity for the associations between the selected IVs of the knee OA and ALM was not observed, as in the study above no directional pleiotropy was detected (Table 4). The results of the leave-one-out test were also consistent with the aforementioned results (Supplementary Figures S6–S8).

## Discussion

We investigated the potential causality between SP-related traits and OA. Following the FDR correction, the results demonstrated a possible causal relationship between SP-related traits and all OA, the usual walking pace and hip OA, the usual walking pace and knee OA, the ALM and knee OA, respectively. In addition, we found evidence of their previous reverse causation with each other. The results also indicated a causal relationship between hand OA and hand grip strength.

Firstly, SP increases the risk of OA. In recent years, studies have demonstrated a correlation of varying intensity between SP and OA (Jin et al., 2017; Vlietstra et al., 2019; Dalle and Koppo, 2020; Godziuk et al., 2021). Decreased muscle strength is the main feature of SP and previous experiments have demonstrated that decreased muscle strength or muscle weakness is a risk factor for the development and progression of OA (Tanaka et al., 2019; Xu et al., 2020). Andrews et al. discovered that in men only, the likelihood of knee OA was reduced for each standard deviation (SD) reduction in ALM (OR per SD reduction: 0.68; 95% CI: 0.47–0.97) (Andrews et al., 2021). Similarly, another study noted significant ALM or total lean mass and increased fat mass (FM) were associated with radiographic knee OA (Azuma et al., 2017). Optimizing medial femoral size is important in clinical management to reduce the progression of OA and subsequent knee arthroplasty, which in part reflects the important impact of SP on the development of OA (Wang et al., 2012).

In addition, OA has been noted as a risk factor for increasing the incidence of SP (Kemmler et al., 2015; Dharmakulsakti et al., 2022). A previous foundational experimental study provided some mechanistic insights, indicating that knee osteoarthritis induced by anterior cruciate ligament transection promotes remodeling and atrophy in the neuromuscular junctions of the quadriceps and tibialis anterior muscles. These changes were associated with signs of inflammation and alterations in muscle gene and protein expression (Cunha et al., 2019). Besides, some studies have indicated a strong relationship between OA and low skeletal muscle mass (Berenbaum and van den Berg, 2015; Jeon et al., 2019). A significant association between OA of the knee and walking pace was observed (*p* < 0.001, OR:0.073) (Nakamura and Ogata, 2016). A recent study conducted a more comprehensive analysis, revealing that the OA group had statistically significantly worse SP parameters than the control group, with lower appendicular skeletal muscle mass (*p* = 0.041), impaired performance on the 40-m fast walk test (*p* = 0.020), and reduced right (*p* < 0.01) and left (*p* < 0.01) hand grip strength. The findings suggest an early onset of sarcopenia in these individuals (de Almeida et al., 2020).

According to previous studies, bone, muscle, and fat tissue are connected and interact with each other through molecules. And SP seems to have a bidirectional relationship with the maintenance or destruction of joint structures (Spanoudaki et al., 2023). SP and OA elevate the risk of mutual development, which is one of the most important reasons for their frequent coexistence (Peng and Zeng, 2022). A new concept of “sarcopenic knee OA” has also been proposed (Iijima and Aoyama, 2021). The study by Jiyong Yang et al. identified a common network of genetic interactions between KOA and SP, including 14 common differentially expressed genes, 4 hub genes, and 10 potential chemical compounds, among other important findings, which have updated the research results of the mechanism between OA and SP (Yang et al., 2023).

These previous studies support our view to some extent. Our study’s methodology thus has some advantages. Firstly, the MR method can effectively avoid the drawbacks of traditional observational research methods such as residual confounding uncertainty and reverse causality. Secondly, the IVs for SP-related traits were obtained from the existing large GWAS, and the IVs for OA were obtained from the most recent GWAS. This allowed for a more precise assessment of effect sizes than would be possible with individual-level data or findings from studies with limited sample sizes. Lastly, we performed an analysis of the specific relationships between individual SP-related traits and the different sites of OA, which led to a more comprehensive understanding of the potential link between them.

Nevertheless, our study also has some limitations. Firstly, due to the lack of data on the large GWAS concerning SP, we could only use the related traits to analyze the relationship with OA. Secondly, selecting SNPs from the different large-sample GWAS studies may increase the risk of sample overlap between exposure and outcome variables, which may bias the results. Furthermore, due to the complexity of biological systems, bidirectional MR assumes that causality happens in one direction, and feedback loops may exist between the exposure and the outcome, which might affect the accuracy of the results. Finally, since a majority of the participants included in the study were of European ancestry and were not representative of other racial groups, further magnetic resonance studies are needed to verify causality.

## Conclusion

This present study suggests an obvious causality of SP on OA, with condition exhibiting site-specific effects, while evidence was also provided for the causal effect of OA on SP. It presents some evidence of reciprocal interaction between SP and OA, which may facilitate the development of novel treatment strategies for both diseases. However, the causal relationship between the two conditions still necessitates further investigation and substantiation through a multitude of studies.

## Data Availability

The original contributions presented in the study are included in the article/Supplementary Material, further inquiries can be directed to the corresponding authors.
