# Supplementary material for: Genetic causality and site-specific relationship between sarcopenia and osteoarthritis: a bidirectional Mendelian randomization study
Source: Front Genet. 2024 Jan 8;14:1340245. doi: 10.3389/fgene.2023.1340245 (PMC10804883; doi:10.3389/fgene.2023.1340245)
Supplement: Supplementary file 1 [file Table1.DOCX]

**Supplementary Table 1. Information such as F-statistics and R2 of IVs in MR analysis.**

| SNP | exposure | Sample size | EAF | SE | SD | R^2^ | F-statistics |
| --- | --- | --- | --- | --- | --- | --- | --- |
| rs10112506 | Appendicular lean mass | 450243 | 0.3898 | 0.0019 | 1.274902832 | 4.21E-05 | 18.97648182 |
| rs10471339 | Appendicular lean mass | 450243 | 0.3823 | 0.0019 | 1.274902832 | 3.52E-05 | 15.83081975 |
| rs1063582 | Appendicular lean mass | 450243 | 0.7649 | 0.0022 | 1.476203279 | 5.65E-05 | 25.43360861 |
| rs10776560 | Appendicular lean mass | 450243 | 0.4995 | 0.0019 | 1.274902832 | 7.58E-05 | 34.14229226 |
| rs10822117 | Appendicular lean mass | 450243 | 0.237 | 0.0022 | 1.476203279 | 5.14E-05 | 23.14745516 |
| rs10845408 | Appendicular lean mass | 450243 | 0.3538 | 0.002 | 1.342002981 | 1.65E-04 | 74.34382864 |
| rs10858246 | Appendicular lean mass | 450243 | 0.3183 | 0.002 | 1.342002981 | 8.52E-05 | 38.34870433 |
| rs10864899 | Appendicular lean mass | 450243 | 0.5643 | 0.0019 | 1.274902832 | 3.79E-05 | 17.0872039 |
| rs11014285 | Appendicular lean mass | 450243 | 0.1654 | 0.0026 | 1.744603875 | 1.06E-04 | 47.77421371 |
| rs11121615 | Appendicular lean mass | 450243 | 0.6894 | 0.002 | 1.342002981 | 9.70E-05 | 43.69036626 |
| rs111365325 | Appendicular lean mass | 450243 | 0.2312 | 0.0022 | 1.476203279 | 1.20E-04 | 53.94789767 |
| rs11178643 | Appendicular lean mass | 450243 | 0.3593 | 0.002 | 1.342002981 | 3.04E-05 | 13.67559413 |
| rs11191208 | Appendicular lean mass | 450243 | 0.2057 | 0.0024 | 1.610403577 | 2.72E-05 | 12.25944845 |
| rs11198591 | Appendicular lean mass | 450243 | 0.3684 | 0.002 | 1.342002981 | 5.66E-05 | 25.48460051 |
| rs11210892 | Appendicular lean mass | 450243 | 0.6745 | 0.002 | 1.342002981 | 3.39E-05 | 15.28550462 |
| rs11217863 | Appendicular lean mass | 450243 | 0.1162 | 0.003 | 2.013004471 | 3.64E-05 | 16.39196739 |
| rs112537273 | Appendicular lean mass | 450243 | 0.2299 | 0.0022 | 1.476203279 | 7.30E-05 | 32.88306104 |
| rs11260035 | Appendicular lean mass | 450243 | 0.2761 | 0.0021 | 1.40910313 | 4.53E-05 | 20.39560776 |
| rs113671109 | Appendicular lean mass | 450243 | 0.22 | 0.0023 | 1.543303428 | 3.24E-05 | 14.59776193 |
| rs115010283 | Appendicular lean mass | 450243 | 0.3162 | 0.002 | 1.342002981 | 2.78E-04 | 125.0078929 |
| rs11562101 | Appendicular lean mass | 450243 | 0.4579 | 0.002 | 1.342002981 | 3.65E-05 | 16.41457489 |
| rs11580040 | Appendicular lean mass | 450243 | 0.08 | 0.0035 | 2.348505216 | 2.82E-05 | 12.69254632 |
| rs11590254 | Appendicular lean mass | 450243 | 0.3114 | 0.002 | 1.342002981 | 8.24E-05 | 37.09499953 |
| rs115912456 | Appendicular lean mass | 450243 | 0.0412 | 0.0047 | 3.153707004 | 2.64E-05 | 11.90750312 |
| rs11605297 | Appendicular lean mass | 450243 | 0.2328 | 0.0022 | 1.476203279 | 3.49E-05 | 15.73240658 |
| rs11612462 | Appendicular lean mass | 450243 | 0.1698 | 0.0025 | 1.677503726 | 2.25E-05 | 10.14987684 |
| rs11629593 | Appendicular lean mass | 450243 | 0.6158 | 0.002 | 1.342002981 | 3.12E-05 | 14.05502664 |
| rs11672848 | Appendicular lean mass | 450243 | 0.5246 | 0.0019 | 1.274902832 | 8.97E-05 | 40.40541034 |
| rs11721522 | Appendicular lean mass | 450243 | 0.4125 | 0.0019 | 1.274902832 | 3.35E-05 | 15.08616907 |
| rs11727162 | Appendicular lean mass | 450243 | 0.4987 | 0.0019 | 1.274902832 | 8.89E-05 | 40.03081125 |
| rs11867855 | Appendicular lean mass | 450243 | 0.2372 | 0.0022 | 1.476203279 | 2.89E-05 | 13.0277226 |
| rs12051245 | Appendicular lean mass | 450243 | 0.2318 | 0.0022 | 1.476203279 | 1.46E-04 | 65.79248249 |
| rs12150907 | Appendicular lean mass | 450243 | 0.1977 | 0.0024 | 1.610403577 | 5.87E-05 | 26.41573832 |
| rs12188208 | Appendicular lean mass | 450243 | 0.2349 | 0.0022 | 1.476203279 | 6.27E-05 | 28.24102051 |
| rs12517711 | Appendicular lean mass | 450243 | 0.3917 | 0.0019 | 1.274902832 | 6.34E-05 | 28.52689068 |
| rs12541381 | Appendicular lean mass | 450243 | 0.2575 | 0.0022 | 1.476203279 | 1.79E-04 | 80.41097321 |
| rs12563442 | Appendicular lean mass | 450243 | 0.2669 | 0.0021 | 1.40910313 | 2.93E-05 | 13.20789694 |
| rs12655296 | Appendicular lean mass | 450243 | 0.6251 | 0.002 | 1.342002981 | 3.15E-05 | 14.1785579 |
| rs12702693 | Appendicular lean mass | 450243 | 0.4542 | 0.0019 | 1.274902832 | 9.13E-05 | 41.10866591 |
| rs12724708 | Appendicular lean mass | 450243 | 0.3574 | 0.002 | 1.342002981 | 1.51E-04 | 67.81742602 |
| rs1290786 | Appendicular lean mass | 450243 | 0.4307 | 0.0019 | 1.274902832 | 6.17E-05 | 27.78022703 |
| rs12943867 | Appendicular lean mass | 450243 | 0.3367 | 0.002 | 1.342002981 | 8.40E-05 | 37.80883564 |
| rs13391980 | Appendicular lean mass | 450243 | 0.1198 | 0.0029 | 1.945904322 | 2.82E-05 | 12.6954335 |
| rs143554698 | Appendicular lean mass | 450243 | 0.1409 | 0.0027 | 1.811704024 | 4.87E-05 | 21.93525335 |
| rs1472852 | Appendicular lean mass | 450243 | 0.1582 | 0.0026 | 1.744603875 | 3.56E-04 | 160.4326849 |
| rs14976 | Appendicular lean mass | 450243 | 0.3057 | 0.002 | 1.342002981 | 4.89E-05 | 22.00679968 |
| rs1514134 | Appendicular lean mass | 450243 | 0.3849 | 0.0019 | 1.274902832 | 3.79E-05 | 17.04671295 |
| rs1556659 | Appendicular lean mass | 450243 | 0.3818 | 0.002 | 1.342002981 | 6.96E-05 | 31.35728508 |
| rs17197114 | Appendicular lean mass | 450243 | 0.1772 | 0.0025 | 1.677503726 | 3.25E-05 | 14.61728389 |
| rs17278379 | Appendicular lean mass | 450243 | 0.124 | 0.0029 | 1.945904322 | 2.93E-05 | 13.19433381 |
| rs173135 | Appendicular lean mass | 450243 | 0.115 | 0.003 | 2.013004471 | 5.84E-05 | 26.30030556 |
| rs17478946 | Appendicular lean mass | 450243 | 0.3004 | 0.0021 | 1.40910313 | 7.80E-05 | 35.13788002 |
| rs1880318 | Appendicular lean mass | 450243 | 0.2039 | 0.0024 | 1.610403577 | 2.71E-05 | 12.17970727 |
| rs2070598 | Appendicular lean mass | 450243 | 0.4559 | 0.0019 | 1.274902832 | 1.27E-04 | 57.19850618 |
| rs2071450 | Appendicular lean mass | 450243 | 0.3675 | 0.002 | 1.342002981 | 7.82E-05 | 35.18992873 |
| rs2089111 | Appendicular lean mass | 450243 | 0.267 | 0.0022 | 1.476203279 | 5.31E-05 | 23.92643053 |
| rs2101017 | Appendicular lean mass | 450243 | 0.8695 | 0.0028 | 1.878804173 | 3.20E-05 | 14.39513403 |
| rs2142331 | Appendicular lean mass | 450243 | 0.6023 | 0.0019 | 1.274902832 | 8.02E-05 | 36.1320047 |
| rs2188805 | Appendicular lean mass | 450243 | 0.3359 | 0.002 | 1.342002981 | 3.22E-05 | 14.49556821 |
| rs2209098 | Appendicular lean mass | 450243 | 0.3108 | 0.002 | 1.342002981 | 1.37E-04 | 61.69874737 |
| rs2230033 | Appendicular lean mass | 450243 | 0.5644 | 0.0019 | 1.274902832 | 2.12E-04 | 95.67087836 |
| rs2236096 | Appendicular lean mass | 450243 | 0.2327 | 0.0023 | 1.543303428 | 4.86E-05 | 21.87258352 |
| rs2237485 | Appendicular lean mass | 450243 | 0.2234 | 0.0023 | 1.543303428 | 5.31E-05 | 23.9300037 |
| rs2268718 | Appendicular lean mass | 450243 | 0.2704 | 0.0021 | 1.40910313 | 3.95E-05 | 17.78837896 |
| rs2289629 | Appendicular lean mass | 450243 | 0.3451 | 0.002 | 1.342002981 | 5.50E-05 | 24.7534269 |
| rs2347808 | Appendicular lean mass | 450243 | 0.5139 | 0.0019 | 1.274902832 | 4.80E-05 | 21.62549158 |
| rs244711 | Appendicular lean mass | 450243 | 0.686 | 0.0022 | 1.476203279 | 1.54E-04 | 69.29656579 |
| rs2569888 | Appendicular lean mass | 450243 | 0.2449 | 0.0022 | 1.476203279 | 3.00E-05 | 13.51737246 |
| rs2578565 | Appendicular lean mass | 450243 | 0.6575 | 0.002 | 1.342002981 | 4.97E-05 | 22.3863983 |
| rs2592208 | Appendicular lean mass | 450243 | 0.513 | 0.0019 | 1.274902832 | 4.73E-05 | 21.28291399 |
| rs2648725 | Appendicular lean mass | 450243 | 0.2134 | 0.0023 | 1.543303428 | 3.84E-05 | 17.27847091 |
| rs2663126 | Appendicular lean mass | 450243 | 0.6905 | 0.0021 | 1.40910313 | 4.16E-05 | 18.72670965 |
| rs2754255 | Appendicular lean mass | 450243 | 0.2245 | 0.0023 | 1.543303428 | 3.42E-05 | 15.40878042 |
| rs2763263 | Appendicular lean mass | 450243 | 0.2445 | 0.0022 | 1.476203279 | 4.90E-05 | 22.06049019 |
| rs2788213 | Appendicular lean mass | 450243 | 0.7104 | 0.0021 | 1.40910313 | 3.14E-05 | 14.11610325 |
| rs2789365 | Appendicular lean mass | 450243 | 0.4807 | 0.0019 | 1.274902832 | 6.46E-05 | 29.07885901 |
| rs28379706 | Appendicular lean mass | 450243 | 0.3931 | 0.002 | 1.342002981 | 3.44E-05 | 15.50289882 |
| rs28485212 | Appendicular lean mass | 450243 | 0.1494 | 0.0027 | 1.811704024 | 2.74E-05 | 12.32264958 |
| rs28529055 | Appendicular lean mass | 450243 | 0.4368 | 0.0019 | 1.274902832 | 6.54E-05 | 29.45297839 |
| rs28529426 | Appendicular lean mass | 450243 | 0.1711 | 0.0026 | 1.744603875 | 2.63E-05 | 11.84304847 |
| rs28678024 | Appendicular lean mass | 450243 | 0.2854 | 0.0021 | 1.40910313 | 2.91E-05 | 13.09824214 |
| rs2871960 | Appendicular lean mass | 450243 | 0.4448 | 0.0019 | 1.274902832 | 6.68E-04 | 301.1418854 |
| rs2923411 | Appendicular lean mass | 450243 | 0.5946 | 0.0019 | 1.274902832 | 4.78E-05 | 21.54059686 |
| rs2925155 | Appendicular lean mass | 450243 | 0.2612 | 0.0022 | 1.476203279 | 3.98E-05 | 17.94248488 |
| rs2978362 | Appendicular lean mass | 450243 | 0.5326 | 0.0019 | 1.274902832 | 3.44E-05 | 15.49663531 |
| rs3116194 | Appendicular lean mass | 450243 | 0.0978 | 0.0032 | 2.147204769 | 3.33E-05 | 14.99782514 |
| rs331917 | Appendicular lean mass | 450243 | 0.5803 | 0.0019 | 1.274902832 | 4.83E-05 | 21.76410633 |
| rs336630 | Appendicular lean mass | 450243 | 0.43 | 0.0019 | 1.274902832 | 3.39E-05 | 15.25775453 |
| rs34312629 | Appendicular lean mass | 450243 | 0.2613 | 0.0021 | 1.40910313 | 5.62E-05 | 25.29992488 |
| rs34338597 | Appendicular lean mass | 450243 | 0.3832 | 0.0019 | 1.274902832 | 3.65E-05 | 16.42640853 |
| rs34345560 | Appendicular lean mass | 450243 | 0.1952 | 0.0024 | 1.610403577 | 5.81E-05 | 26.16295712 |
| rs34517439 | Appendicular lean mass | 450243 | 0.1221 | 0.0029 | 1.945904322 | 1.00E-04 | 45.18565254 |
| rs35073631 | Appendicular lean mass | 450243 | 0.4331 | 0.0019 | 1.274902832 | 3.79E-05 | 17.06349578 |
| rs35288270 | Appendicular lean mass | 450243 | 0.1344 | 0.0028 | 1.878804173 | 7.09E-05 | 31.93059463 |
| rs35732917 | Appendicular lean mass | 450243 | 0.2843 | 0.0021 | 1.40910313 | 8.53E-05 | 38.40561576 |
| rs35756741 | Appendicular lean mass | 450243 | 0.0924 | 0.0033 | 2.214304918 | 4.89E-05 | 22.00753861 |
| rs35811052 | Appendicular lean mass | 450243 | 0.2559 | 0.0022 | 1.476203279 | 3.83E-05 | 17.23551842 |
| rs36000545 | Appendicular lean mass | 450243 | 0.3957 | 0.002 | 1.342002981 | 1.29E-04 | 57.87458669 |
| rs36048468 | Appendicular lean mass | 450243 | 0.2088 | 0.0023 | 1.543303428 | 8.95E-05 | 40.29911146 |
| rs36226649 | Appendicular lean mass | 450243 | 0.0667 | 0.0038 | 2.549805663 | 4.50E-05 | 20.28201098 |
| rs3782232 | Appendicular lean mass | 450243 | 0.0712 | 0.0037 | 2.482705514 | 2.47E-05 | 11.10291269 |
| rs3828729 | Appendicular lean mass | 450243 | 0.3094 | 0.002 | 1.342002981 | 6.07E-05 | 27.3515099 |
| rs40270 | Appendicular lean mass | 450243 | 0.7724 | 0.0022 | 1.476203279 | 3.68E-05 | 16.56407161 |
| rs4282339 | Appendicular lean mass | 450243 | 0.2075 | 0.0023 | 1.543303428 | 1.34E-04 | 60.14071005 |
| rs4287835 | Appendicular lean mass | 450243 | 0.534 | 0.0019 | 1.274902832 | 6.62E-05 | 29.79280846 |
| rs4360494 | Appendicular lean mass | 450243 | 0.5541 | 0.0019 | 1.274902832 | 1.19E-04 | 53.66963396 |
| rs45474992 | Appendicular lean mass | 450243 | 0.0362 | 0.0051 | 3.422107601 | 2.27E-05 | 10.21323644 |
| rs45528934 | Appendicular lean mass | 450243 | 0.1623 | 0.0026 | 1.744603875 | 6.13E-05 | 27.61325623 |
| rs4640244 | Appendicular lean mass | 450243 | 0.3993 | 0.0019 | 1.274902832 | 1.18E-04 | 53.16050192 |
| rs4682483 | Appendicular lean mass | 450243 | 0.1534 | 0.0026 | 1.744603875 | 2.32E-05 | 10.46075361 |
| rs4735761 | Appendicular lean mass | 450243 | 0.2857 | 0.0021 | 1.40910313 | 2.25E-04 | 101.422468 |
| rs4752689 | Appendicular lean mass | 450243 | 0.5839 | 0.0019 | 1.274902832 | 1.26E-04 | 56.5743203 |
| rs4847378 | Appendicular lean mass | 450243 | 0.6121 | 0.0019 | 1.274902832 | 5.40E-05 | 24.33124374 |
| rs4870941 | Appendicular lean mass | 450243 | 0.238 | 0.0023 | 1.543303428 | 1.34E-04 | 60.4888829 |
| rs4932439 | Appendicular lean mass | 450243 | 0.8254 | 0.0025 | 1.677503726 | 2.34E-05 | 10.51527876 |
| rs496783 | Appendicular lean mass | 450243 | 0.4648 | 0.0019 | 1.274902832 | 4.71E-05 | 21.1917538 |
| rs4976262 | Appendicular lean mass | 450243 | 0.3156 | 0.002 | 1.342002981 | 1.44E-04 | 64.83503857 |
| rs55758152 | Appendicular lean mass | 450243 | 0.3263 | 0.002 | 1.342002981 | 5.13E-05 | 23.11053462 |
| rs55852614 | Appendicular lean mass | 450243 | 0.2474 | 0.0022 | 1.476203279 | 2.64E-04 | 118.8629085 |
| rs56112295 | Appendicular lean mass | 450243 | 0.2257 | 0.0024 | 1.610403577 | 3.20E-05 | 14.39135627 |
| rs568267 | Appendicular lean mass | 450243 | 0.7431 | 0.0022 | 1.476203279 | 2.61E-05 | 11.74153987 |
| rs57059662 | Appendicular lean mass | 450243 | 0.6774 | 0.002 | 1.342002981 | 3.38E-05 | 15.21445221 |
| rs57513571 | Appendicular lean mass | 450243 | 0.2004 | 0.0024 | 1.610403577 | 4.51E-05 | 20.29842771 |
| rs591668 | Appendicular lean mass | 450243 | 0.396 | 0.0019 | 1.274902832 | 8.91E-05 | 40.12270324 |
| rs599004 | Appendicular lean mass | 450243 | 0.2807 | 0.0021 | 1.40910313 | 5.01E-05 | 22.5716354 |
| rs59950280 | Appendicular lean mass | 450243 | 0.3316 | 0.002 | 1.342002981 | 1.59E-04 | 71.50813338 |
| rs604723 | Appendicular lean mass | 450243 | 0.7248 | 0.0021 | 1.40910313 | 5.54E-05 | 24.92850949 |
| rs6142059 | Appendicular lean mass | 450243 | 0.4926 | 0.0019 | 1.274902832 | 4.14E-05 | 18.63372522 |
| rs61919240 | Appendicular lean mass | 450243 | 0.3244 | 0.002 | 1.342002981 | 4.57E-05 | 20.56835386 |
| rs62033029 | Appendicular lean mass | 450243 | 0.2064 | 0.0023 | 1.543303428 | 2.73E-05 | 12.31214858 |
| rs62143873 | Appendicular lean mass | 450243 | 0.5033 | 0.0019 | 1.274902832 | 4.07E-05 | 18.31704041 |
| rs62466110 | Appendicular lean mass | 450243 | 0.067 | 0.0041 | 2.75110611 | 2.27E-05 | 10.23704219 |
| rs62501195 | Appendicular lean mass | 450243 | 0.1712 | 0.0025 | 1.677503726 | 3.95E-05 | 17.80119275 |
| rs62515437 | Appendicular lean mass | 450243 | 0.2253 | 0.0023 | 1.543303428 | 2.00E-04 | 89.86830513 |
| rs6582398 | Appendicular lean mass | 450243 | 0.6002 | 0.002 | 1.342002981 | 5.22E-05 | 23.51719992 |
| rs6675858 | Appendicular lean mass | 450243 | 0.2141 | 0.0023 | 1.543303428 | 2.65E-05 | 11.94012853 |
| rs6738207 | Appendicular lean mass | 450243 | 0.4007 | 0.0019 | 1.274902832 | 4.77E-05 | 21.45915542 |
| rs67527161 | Appendicular lean mass | 450243 | 0.209 | 0.0023 | 1.543303428 | 4.60E-05 | 20.70417408 |
| rs67551338 | Appendicular lean mass | 450243 | 0.0615 | 0.004 | 2.684005961 | 5.32E-05 | 23.93787158 |
| rs67716382 | Appendicular lean mass | 450243 | 0.2214 | 0.0023 | 1.543303428 | 7.39E-05 | 33.28996959 |
| rs6789000 | Appendicular lean mass | 450243 | 0.6449 | 0.002 | 1.342002981 | 3.72E-05 | 16.76478684 |
| rs68049170 | Appendicular lean mass | 450243 | 0.2764 | 0.0021 | 1.40910313 | 1.35E-04 | 60.85332266 |
| rs6821305 | Appendicular lean mass | 450243 | 0.399 | 0.0019 | 1.274902832 | 1.23E-04 | 55.2944955 |
| rs684905 | Appendicular lean mass | 450243 | 0.4176 | 0.0019 | 1.274902832 | 4.17E-05 | 18.7622463 |
| rs6849302 | Appendicular lean mass | 450243 | 0.1977 | 0.0024 | 1.610403577 | 2.94E-05 | 13.23199122 |
| rs6874142 | Appendicular lean mass | 450243 | 0.1138 | 0.0031 | 2.08010462 | 3.87E-05 | 17.40926574 |
| rs700677 | Appendicular lean mass | 450243 | 0.3505 | 0.002 | 1.342002981 | 7.57E-05 | 34.06907327 |
| rs7014590 | Appendicular lean mass | 450243 | 0.2614 | 0.0022 | 1.476203279 | 9.21E-05 | 41.47699569 |
| rs702886 | Appendicular lean mass | 450243 | 0.3505 | 0.002 | 1.342002981 | 3.64E-05 | 16.39130591 |
| rs71414738 | Appendicular lean mass | 450243 | 0.1763 | 0.0025 | 1.677503726 | 2.32E-05 | 10.45591469 |
| rs718603 | Appendicular lean mass | 450243 | 0.2774 | 0.0021 | 1.40910313 | 3.46E-05 | 15.60096744 |
| rs7229520 | Appendicular lean mass | 450243 | 0.6616 | 0.002 | 1.342002981 | 1.25E-04 | 56.1751376 |
| rs72656010 | Appendicular lean mass | 450243 | 0.1322 | 0.0028 | 1.878804173 | 2.90E-04 | 130.6297256 |
| rs72721979 | Appendicular lean mass | 450243 | 0.143 | 0.0027 | 1.811704024 | 3.92E-05 | 17.63215397 |
| rs72801843 | Appendicular lean mass | 450243 | 0.3015 | 0.0021 | 1.40910313 | 2.08E-04 | 93.58842604 |
| rs72841270 | Appendicular lean mass | 450243 | 0.1354 | 0.0028 | 1.878804173 | 5.73E-05 | 25.81460355 |
| rs72894003 | Appendicular lean mass | 450243 | 0.0647 | 0.0038 | 2.549805663 | 3.33E-05 | 14.99723818 |
| rs73052033 | Appendicular lean mass | 450243 | 0.1851 | 0.0024 | 1.610403577 | 2.65E-05 | 11.94212831 |
| rs7320878 | Appendicular lean mass | 450243 | 0.6034 | 0.0019 | 1.274902832 | 6.63E-05 | 29.832536 |
| rs7328187 | Appendicular lean mass | 450243 | 0.4976 | 0.0019 | 1.274902832 | 4.14E-05 | 18.63737838 |
| rs73384223 | Appendicular lean mass | 450243 | 0.1967 | 0.0024 | 1.610403577 | 5.12E-05 | 23.05778964 |
| rs73413540 | Appendicular lean mass | 450243 | 0.2248 | 0.0023 | 1.543303428 | 2.25E-05 | 10.13061025 |
| rs73696333 | Appendicular lean mass | 450243 | 0.2012 | 0.0024 | 1.610403577 | 4.52E-05 | 20.35907244 |
| rs7418410 | Appendicular lean mass | 450243 | 0.4089 | 0.0019 | 1.274902832 | 7.15E-05 | 32.17313367 |
| rs75022676 | Appendicular lean mass | 450243 | 0.2079 | 0.0023 | 1.543303428 | 3.67E-05 | 16.54238264 |
| rs75702986 | Appendicular lean mass | 450243 | 0.1861 | 0.0025 | 1.677503726 | 2.86E-05 | 12.8781388 |
| rs7598430 | Appendicular lean mass | 450243 | 0.5046 | 0.0019 | 1.274902832 | 7.87E-05 | 35.45669715 |
| rs7610055 | Appendicular lean mass | 450243 | 0.1207 | 0.0029 | 1.945904322 | 7.80E-05 | 35.11785249 |
| rs76364830 | Appendicular lean mass | 450243 | 0.0634 | 0.0039 | 2.616905812 | 3.85E-05 | 17.32210977 |
| rs7689420 | Appendicular lean mass | 450243 | 0.8312 | 0.0025 | 1.677503726 | 2.17E-04 | 97.51960081 |
| rs7701233 | Appendicular lean mass | 450243 | 0.4283 | 0.0019 | 1.274902832 | 9.65E-05 | 43.46954767 |
| rs772222 | Appendicular lean mass | 450243 | 0.2651 | 0.0021 | 1.40910313 | 2.87E-05 | 12.93631764 |
| rs7731023 | Appendicular lean mass | 450243 | 0.5749 | 0.0019 | 1.274902832 | 8.29E-05 | 37.31268003 |
| rs7768973 | Appendicular lean mass | 450243 | 0.411 | 0.0019 | 1.274902832 | 1.72E-04 | 77.26360813 |
| rs7828086 | Appendicular lean mass | 450243 | 0.2391 | 0.0022 | 1.476203279 | 3.04E-05 | 13.70157897 |
| rs78766798 | Appendicular lean mass | 450243 | 0.0847 | 0.0035 | 2.348505216 | 2.86E-05 | 12.88049759 |
| rs7971536 | Appendicular lean mass | 450243 | 0.4945 | 0.0019 | 1.274902832 | 1.16E-04 | 52.12691921 |
| rs8017006 | Appendicular lean mass | 450243 | 0.3275 | 0.002 | 1.342002981 | 3.64E-05 | 16.39106376 |
| rs8018486 | Appendicular lean mass | 450243 | 0.1914 | 0.0024 | 1.610403577 | 2.27E-05 | 10.23409155 |
| rs8019890 | Appendicular lean mass | 450243 | 0.5313 | 0.0019 | 1.274902832 | 1.92E-04 | 86.24200227 |
| rs80295797 | Appendicular lean mass | 450243 | 0.3265 | 0.002 | 1.342002981 | 9.57E-05 | 43.10829249 |
| rs8084413 | Appendicular lean mass | 450243 | 0.4693 | 0.0019 | 1.274902832 | 4.94E-05 | 22.25611802 |
| rs861674 | Appendicular lean mass | 450243 | 0.4578 | 0.0019 | 1.274902832 | 5.00E-05 | 22.53190123 |
| rs900399 | Appendicular lean mass | 450243 | 0.3986 | 0.0019 | 1.274902832 | 7.93E-05 | 35.72265546 |
| rs9266244 | Appendicular lean mass | 450243 | 0.7077 | 0.0021 | 1.40910313 | 3.80E-04 | 171.1150708 |
| rs9343327 | Appendicular lean mass | 450243 | 0.4985 | 0.0019 | 1.274902832 | 6.03E-05 | 27.14808634 |
| rs9375188 | Appendicular lean mass | 450243 | 0.4844 | 0.0019 | 1.274902832 | 5.68E-05 | 25.59413235 |
| rs9385002 | Appendicular lean mass | 450243 | 0.2394 | 0.0022 | 1.476203279 | 3.61E-05 | 16.25973732 |
| rs9391254 | Appendicular lean mass | 450243 | 0.3205 | 0.002 | 1.342002981 | 6.66E-05 | 30.00756047 |
| rs951366 | Appendicular lean mass | 450243 | 0.3932 | 0.0019 | 1.274902832 | 1.23E-04 | 55.5573157 |
| rs9568031 | Appendicular lean mass | 450243 | 0.706 | 0.0021 | 1.40910313 | 2.76E-05 | 12.44942226 |
| rs9636364 | Appendicular lean mass | 450243 | 0.5429 | 0.0019 | 1.274902832 | 3.69E-05 | 16.6361698 |
| rs9809116 | Appendicular lean mass | 450243 | 0.4081 | 0.0019 | 1.274902832 | 7.61E-05 | 34.26169239 |
| rs10203320 | Appendicular lean mass | 450243 | 0.3289 | 0.002 | 1.342002981 | 4.67E-05 | 21.01830228 |
| rs1047891 | Appendicular lean mass | 450243 | 0.3159 | 0.002 | 1.342002981 | 1.30E-04 | 58.66859942 |
| rs11243202 | Appendicular lean mass | 450243 | 0.486 | 0.0019 | 1.274902832 | 2.80E-04 | 126.2571283 |
| rs117068593 | Appendicular lean mass | 450243 | 0.1895 | 0.0024 | 1.610403577 | 1.92E-04 | 86.62863299 |
| rs12347137 | Appendicular lean mass | 450243 | 0.2023 | 0.0024 | 1.610403577 | 2.63E-04 | 118.5962896 |
| rs1341215 | Appendicular lean mass | 450243 | 0.1374 | 0.0027 | 1.811704024 | 3.79E-05 | 17.05234605 |
| rs2871865 | Appendicular lean mass | 450243 | 0.1159 | 0.003 | 2.013004471 | 1.23E-04 | 55.34999787 |
| rs3764002 | Appendicular lean mass | 450243 | 0.2617 | 0.0021 | 1.40910313 | 1.53E-04 | 68.70817299 |
| rs42039 | Appendicular lean mass | 450243 | 0.2444 | 0.0022 | 1.476203279 | 3.92E-04 | 176.6185594 |
| rs59985551 | Appendicular lean mass | 450243 | 0.2261 | 0.0022 | 1.476203279 | 1.57E-04 | 70.84759658 |
| rs62106258 | Appendicular lean mass | 450243 | 0.0486 | 0.0044 | 2.952406557 | 2.69E-05 | 12.1337462 |
| rs6977416 | Appendicular lean mass | 450243 | 0.3338 | 0.002 | 1.342002981 | 5.16E-04 | 232.3354526 |
| rs7129320 | Appendicular lean mass | 450243 | 0.1661 | 0.0025 | 1.677503726 | 1.49E-04 | 67.08038675 |
| rs7301341 | Appendicular lean mass | 450243 | 0.3268 | 0.002 | 1.342002981 | 1.59E-04 | 71.53911959 |
| rs7952436 | Appendicular lean mass | 450243 | 0.0823 | 0.0034 | 2.281405067 | 5.96E-05 | 26.81594269 |
| rs1478575 | Appendicular lean mass | 450243 | 0.6843 | 0.002 | 1.342002981 | 2.34E-04 | 105.1719243 |
| rs28592876 | Appendicular lean mass | 450243 | 0.2051 | 0.0023 | 1.543303428 | 1.23E-04 | 55.48129305 |
| rs6505216 | Appendicular lean mass | 450243 | 0.233 | 0.0023 | 1.543303428 | 3.72E-04 | 167.6270202 |
| rs905938 | Appendicular lean mass | 450243 | 0.2649 | 0.0021 | 1.40910313 | 3.04E-04 | 137.1331834 |
| rs9634212 | Appendicular lean mass | 450243 | 0.221 | 0.0023 | 1.543303428 | 3.21E-04 | 144.4385901 |
| rs9894577 | Appendicular lean mass | 450243 | 0.318 | 0.002 | 1.342002981 | 2.31E-04 | 104.2325798 |
| rs4752829 | Appendicular lean mass | 450243 | 0.2861 | 0.0021 | 1.40910313 | 1.41E-04 | 63.59290078 |
| rs10097417 | Hand grip strength (left) | 461026 | 0.170904 | 0.00197055 | 1.337981684 | 2.77E-05 | 12.77482895 |
| rs10176878 | Hand grip strength (left) | 461026 | 0.190861 | 0.0018962 | 1.287498855 | 3.12E-05 | 14.40143231 |
| rs10403906 | Hand grip strength (left) | 461026 | 0.476375 | 0.00148665 | 1.009418929 | 4.93E-05 | 22.7196899 |
| rs10786706 | Hand grip strength (left) | 461026 | 0.465745 | 0.00148741 | 1.00993496 | 4.89E-05 | 22.52733897 |
| rs10831903 | Hand grip strength (left) | 461026 | 0.423383 | 0.00151154 | 1.026318964 | 4.00E-05 | 18.43072387 |
| rs10846071 | Hand grip strength (left) | 461026 | 0.393672 | 0.00151685 | 1.029924395 | 1.25E-04 | 57.46427888 |
| rs10934857 | Hand grip strength (left) | 461026 | 0.258913 | 0.00170182 | 1.155516982 | 2.48E-05 | 11.42778842 |
| rs10988217 | Hand grip strength (left) | 461026 | 0.603742 | 0.00152911 | 1.0382488 | 3.76E-05 | 17.34571258 |
| rs11121542 | Hand grip strength (left) | 461026 | 0.122677 | 0.00225916 | 1.533944686 | 2.27E-05 | 10.47573731 |
| rs11642954 | Hand grip strength (left) | 461026 | 0.195019 | 0.00187683 | 1.274346839 | 2.65E-05 | 12.20701819 |
| rs12473732 | Hand grip strength (left) | 461026 | 0.486507 | 0.00148601 | 1.008984376 | 5.92E-05 | 27.31393941 |
| rs12889267 | Hand grip strength (left) | 461026 | 0.167084 | 0.00198726 | 1.349327589 | 2.89E-05 | 13.30463198 |
| rs12906830 | Hand grip strength (left) | 461026 | 0.601065 | 0.00151797 | 1.030684863 | 5.30E-05 | 24.45766154 |
| rs13091492 | Hand grip strength (left) | 461026 | 0.372833 | 0.00153407 | 1.041616585 | 3.10E-05 | 14.28700195 |
| rs13146142 | Hand grip strength (left) | 461026 | 0.158623 | 0.00202888 | 1.37758711 | 5.74E-05 | 26.46873252 |
| rs13337177 | Hand grip strength (left) | 461026 | 0.180836 | 0.00193621 | 1.314665203 | 3.50E-05 | 16.11552497 |
| rs16870531 | Hand grip strength (left) | 461026 | 0.238492 | 0.00174241 | 1.183077144 | 3.25E-05 | 15.00274565 |
| rs17282763 | Hand grip strength (left) | 461026 | 0.296281 | 0.00163298 | 1.108775383 | 2.71E-05 | 12.49722297 |
| rs1884447 | Hand grip strength (left) | 461026 | 0.400566 | 0.00151391 | 1.027928168 | 3.25E-05 | 14.99923631 |
| rs2359239 | Hand grip strength (left) | 461026 | 0.391621 | 0.00152153 | 1.033102064 | 3.47E-05 | 15.9929481 |
| rs2974438 | Hand grip strength (left) | 461026 | 0.211026 | 0.00182387 | 1.238387584 | 2.20E-05 | 10.15802696 |
| rs35175534 | Hand grip strength (left) | 461026 | 0.139986 | 0.00235477 | 1.59886282 | 2.53E-05 | 11.64138502 |
| rs4121165 | Hand grip strength (left) | 461026 | 0.211447 | 0.00181868 | 1.234863631 | 2.85E-05 | 13.14185648 |
| rs4575361 | Hand grip strength (left) | 461026 | 0.31205 | 0.00160223 | 1.087896472 | 4.23E-05 | 19.51868311 |
| rs4962700 | Hand grip strength (left) | 461026 | 0.301977 | 0.00163488 | 1.110065462 | 2.80E-05 | 12.8986389 |
| rs59116179 | Hand grip strength (left) | 461026 | 0.617442 | 0.00153635 | 1.04316468 | 3.19E-05 | 14.71319059 |
| rs61286123 | Hand grip strength (left) | 461026 | 0.227916 | 0.00177111 | 1.202564127 | 2.48E-05 | 11.45466705 |
| rs62081464 | Hand grip strength (left) | 461026 | 0.227338 | 0.00177979 | 1.208457751 | 2.35E-05 | 10.83212353 |
| rs6689375 | Hand grip strength (left) | 461026 | 0.185489 | 0.00191158 | 1.297941705 | 4.60E-05 | 21.21461632 |
| rs7197751 | Hand grip strength (left) | 461026 | 0.363019 | 0.00156268 | 1.061042459 | 3.68E-05 | 16.96042655 |
| rs73307079 | Hand grip strength (left) | 461026 | 0.211119 | 0.00183407 | 1.245313271 | 2.65E-05 | 12.20641626 |
| rs772014 | Hand grip strength (left) | 461026 | 0.392538 | 0.00151891 | 1.031323113 | 5.05E-05 | 23.30536369 |
| rs7856625 | Hand grip strength (left) | 461026 | 0.609861 | 0.0015209 | 1.032674301 | 5.53E-05 | 25.48402027 |
| rs821100 | Hand grip strength (left) | 461026 | 0.265512 | 0.00168615 | 1.144877225 | 3.09E-05 | 14.26666486 |
| rs9371201 | Hand grip strength (left) | 461026 | 0.335102 | 0.0015738 | 1.068592816 | 3.40E-05 | 15.69015783 |
| rs9371881 | Hand grip strength (left) | 461026 | 0.359174 | 0.00154996 | 1.05240572 | 3.73E-05 | 17.21930281 |
| rs9611273 | Hand grip strength (left) | 461026 | 0.25267 | 0.00173048 | 1.174976806 | 3.18E-05 | 14.67983428 |
| rs9944324 | Hand grip strength (left) | 461026 | 0.456723 | 0.00150169 | 1.019630923 | 3.49E-05 | 16.09775854 |
| rs11002322 | Hand grip strength (left) | 461026 | 0.340459 | 0.00157255 | 1.06774408 | 3.93E-05 | 18.12206858 |
| rs12673062 | Hand grip strength (left) | 461026 | 0.215756 | 0.00180808 | 1.227666348 | 2.60E-05 | 12.00541127 |
| rs12790261 | Hand grip strength (left) | 461026 | 0.082383 | 0.00270305 | 1.835341093 | 2.85E-05 | 13.12881838 |
| rs17466480 | Hand grip strength (left) | 461026 | 0.386968 | 0.00152732 | 1.03703341 | 6.17E-05 | 28.43446939 |
| rs181766 | Hand grip strength (left) | 461026 | 0.321793 | 0.00160372 | 1.088908166 | 3.42E-05 | 15.74708042 |
| rs3814877 | Hand grip strength (left) | 461026 | 0.401594 | 0.00151385 | 1.027887428 | 5.05E-05 | 23.29298344 |
| rs4621706 | Hand grip strength (left) | 461026 | 0.544473 | 0.00150003 | 1.018503801 | 6.55E-05 | 30.21506646 |
| rs4713506 | Hand grip strength (left) | 461026 | 0.255607 | 0.00169811 | 1.152997933 | 7.08E-05 | 32.64246693 |
| rs56338231 | Hand grip strength (left) | 461026 | 0.258266 | 0.00169739 | 1.152509061 | 3.39E-05 | 15.64639448 |
| rs755547 | Hand grip strength (left) | 461026 | 0.188906 | 0.00189766 | 1.288490179 | 5.03E-05 | 23.21233467 |
| rs7963801 | Hand grip strength (left) | 461026 | 0.571927 | 0.00150824 | 1.0240783 | 5.08E-05 | 23.43883257 |
| rs9388769 | Hand grip strength (left) | 461026 | 0.67318 | 0.00158065 | 1.073243891 | 7.58E-05 | 34.92723929 |
| rs10278546 | Hand grip strength (right) | 461089 | 0.194705 | 0.00188373 | 1.279119251 | 2.22E-05 | 10.2203207 |
| rs1043515 | Hand grip strength (right) | 461089 | 0.566183 | 0.00149848 | 1.017520884 | 9.07E-05 | 41.83637324 |
| rs10770125 | Hand grip strength (right) | 461089 | 0.477072 | 0.00148775 | 1.010234835 | 3.48E-05 | 16.06311899 |
| rs10799428 | Hand grip strength (right) | 461089 | 0.18721 | 0.00190367 | 1.292659216 | 3.75E-05 | 17.31429352 |
| rs113315602 | Hand grip strength (right) | 461089 | 0.095826 | 0.00266761 | 1.811401477 | 2.39E-05 | 11.0388901 |
| rs113835839 | Hand grip strength (right) | 461089 | 0.247871 | 0.00172486 | 1.171240905 | 2.64E-05 | 12.19311549 |
| rs12412806 | Hand grip strength (right) | 461089 | 0.294214 | 0.00164139 | 1.114561825 | 2.77E-05 | 12.7537132 |
| rs12452505 | Hand grip strength (right) | 461089 | 0.142315 | 0.00213545 | 1.450046028 | 2.42E-05 | 11.14934106 |
| rs12823922 | Hand grip strength (right) | 461089 | 0.222184 | 0.00178649 | 1.213089854 | 3.06E-05 | 14.12488483 |
| rs13169333 | Hand grip strength (right) | 461089 | 0.260299 | 0.00169626 | 1.151820495 | 2.51E-05 | 11.55292314 |
| rs1442883 | Hand grip strength (right) | 461089 | 0.253067 | 0.00171426 | 1.164043131 | 3.15E-05 | 14.506566 |
| rs1885690 | Hand grip strength (right) | 461089 | 0.410365 | 0.00151212 | 1.026782926 | 3.24E-05 | 14.93291439 |
| rs2194747 | Hand grip strength (right) | 461089 | 0.707014 | 0.00163925 | 1.11310869 | 3.24E-05 | 14.96271022 |
| rs2226685 | Hand grip strength (right) | 461089 | 0.758696 | 0.00174324 | 1.183721575 | 2.79E-05 | 12.86578402 |
| rs248831 | Hand grip strength (right) | 461089 | 0.265259 | 0.00171686 | 1.165808623 | 2.81E-05 | 12.93474453 |
| rs2854152 | Hand grip strength (right) | 461089 | 0.678266 | 0.00159765 | 1.084860819 | 4.48E-05 | 20.65645074 |
| rs35833641 | Hand grip strength (right) | 461089 | 0.312389 | 0.00160181 | 1.087685606 | 3.09E-05 | 14.24321869 |
| rs3848369 | Hand grip strength (right) | 461089 | 0.38657 | 0.00153126 | 1.039779663 | 3.99E-05 | 18.40066446 |
| rs4549685 | Hand grip strength (right) | 461089 | 0.329917 | 0.00158124 | 1.073717849 | 3.62E-05 | 16.6902095 |
| rs4751671 | Hand grip strength (right) | 461089 | 0.530724 | 0.00150019 | 1.018682034 | 3.25E-05 | 15.00320739 |
| rs4868110 | Hand grip strength (right) | 461089 | 0.323165 | 0.00158981 | 1.079537182 | 3.54E-05 | 16.32151031 |
| rs56074046 | Hand grip strength (right) | 461089 | 0.371748 | 0.00153954 | 1.045402075 | 3.49E-05 | 16.08800037 |
| rs56365901 | Hand grip strength (right) | 461089 | 0.222684 | 0.00179293 | 1.217462841 | 4.75E-05 | 21.90571236 |
| rs600038 | Hand grip strength (right) | 461089 | 0.20706 | 0.00183358 | 1.245065628 | 2.28E-05 | 10.51581951 |
| rs62037412 | Hand grip strength (right) | 461089 | 0.357285 | 0.0015542 | 1.055356733 | 3.59E-05 | 16.54987599 |
| rs6693965 | Hand grip strength (right) | 461089 | 0.12803 | 0.00223033 | 1.514472901 | 2.57E-05 | 11.85426126 |
| rs6792762 | Hand grip strength (right) | 461089 | 0.419501 | 0.0015155 | 1.029078066 | 3.82E-05 | 17.62605097 |
| rs721101 | Hand grip strength (right) | 461089 | 0.271238 | 0.0016742 | 1.136840975 | 2.74E-05 | 12.64106304 |
| rs7266065 | Hand grip strength (right) | 461089 | 0.322982 | 0.00159481 | 1.082932359 | 3.68E-05 | 16.9853117 |
| rs7301953 | Hand grip strength (right) | 461089 | 0.312268 | 0.00160449 | 1.089505421 | 4.82E-05 | 22.21829662 |
| rs7790322 | Hand grip strength (right) | 461089 | 0.415619 | 0.0015092 | 1.024800143 | 3.42E-05 | 15.7848959 |
| rs7871404 | Hand grip strength (right) | 461089 | 0.188654 | 0.00190074 | 1.290669642 | 2.62E-05 | 12.06909278 |
| rs935728 | Hand grip strength (right) | 461089 | 0.327604 | 0.0015872 | 1.0777649 | 3.46E-05 | 15.96126651 |
| rs9639938 | Hand grip strength (right) | 461089 | 0.540731 | 0.0014948 | 1.015022034 | 3.67E-05 | 16.94281709 |
| rs9652468 | Hand grip strength (right) | 461089 | 0.249359 | 0.00172118 | 1.168742055 | 4.30E-05 | 19.84308286 |
| rs9757079 | Hand grip strength (right) | 461089 | 0.318301 | 0.00159699 | 1.084412656 | 3.55E-05 | 16.35006771 |
| rs9853018 | Hand grip strength (right) | 461089 | 0.443249 | 0.00149426 | 1.014655355 | 4.99E-05 | 22.98850112 |
| rs10784502 | Hand grip strength (right) | 461089 | 0.512218 | 0.00148608 | 1.009100846 | 6.07E-05 | 28.00446834 |
| rs11039348 | Hand grip strength (right) | 461089 | 0.348363 | 0.00156104 | 1.060001335 | 3.86E-05 | 17.77930416 |
| rs1125 | Hand grip strength (right) | 461089 | 0.336554 | 0.00157472 | 1.069290539 | 3.93E-05 | 18.11904621 |
| rs1550115 | Hand grip strength (right) | 461089 | 0.748561 | 0.00171276 | 1.163024578 | 6.50E-05 | 29.97605554 |
| rs1952256 | Hand grip strength (right) | 461089 | 0.345023 | 0.00156323 | 1.061488423 | 3.83E-05 | 17.64722273 |
| rs246181 | Hand grip strength (right) | 461089 | 0.373429 | 0.00154856 | 1.051526974 | 3.99E-05 | 18.3779852 |
| rs645144 | Hand grip strength (right) | 461089 | 0.329996 | 0.00158627 | 1.077133397 | 2.88E-05 | 13.26493364 |
| rs6473015 | Hand grip strength (right) | 461089 | 0.285659 | 0.00164629 | 1.117889098 | 3.01E-05 | 13.87679491 |
| rs6693567 | Hand grip strength (right) | 461089 | 0.733032 | 0.00167765 | 1.139183647 | 2.87E-05 | 13.21060464 |
| rs8055199 | Hand grip strength (right) | 461089 | 0.660123 | 0.00157271 | 1.067925678 | 3.16E-05 | 14.57340887 |
| rs10798483 | Hand grip strength (right) | 461089 | 0.54729 | 0.00149469 | 1.01494734 | 1.01E-04 | 46.69178592 |
| rs6870324 | Hand grip strength (right) | 461089 | 0.269673 | 0.00168316 | 1.142925132 | 3.01E-05 | 13.86438084 |
| rs9322822 | Hand grip strength (right) | 461089 | 0.320183 | 0.00159266 | 1.081472433 | 4.57E-05 | 21.06996349 |
| rs10828258 | Usual walking pace | 459915 | 0.319319 | 0.00135882 | 0.921511411 | 4.45E-05 | 20.47957692 |
| rs10862220 | Usual walking pace | 459915 | 0.674897 | 0.00135051 | 0.915875816 | 3.70E-05 | 17.00819978 |
| rs11039324 | Usual walking pace | 459915 | 0.403963 | 0.00128765 | 0.873246029 | 6.58E-05 | 30.28097567 |
| rs11150623 | Usual walking pace | 459915 | 0.644857 | 0.0013224 | 0.896812448 | 5.35E-05 | 24.58511443 |
| rs113825410 | Usual walking pace | 459915 | 0.224386 | 0.00151778 | 1.029313367 | 2.55E-05 | 11.73259732 |
| rs11682482 | Usual walking pace | 459915 | 0.680801 | 0.00135595 | 0.919565062 | 3.31E-05 | 15.20095052 |
| rs11732213 | Usual walking pace | 459915 | 0.195605 | 0.00159593 | 1.082312379 | 2.25E-05 | 10.37010676 |
| rs11761141 | Usual walking pace | 459915 | 0.325697 | 0.00135132 | 0.916425134 | 3.26E-05 | 15.00992544 |
| rs11848096 | Usual walking pace | 459915 | 0.386669 | 0.00130696 | 0.886341498 | 3.40E-05 | 15.65441919 |
| rs12461902 | Usual walking pace | 459915 | 0.329608 | 0.00135739 | 0.920541628 | 3.35E-05 | 15.39466516 |
| rs205262 | Usual walking pace | 459915 | 0.26893 | 0.00142654 | 0.967437106 | 3.19E-05 | 14.65742581 |
| rs2170670 | Usual walking pace | 459915 | 0.606925 | 0.0012972 | 0.879722555 | 3.10E-05 | 14.24451138 |
| rs2280406 | Usual walking pace | 459915 | 0.506176 | 0.00126505 | 0.857919379 | 6.74E-05 | 30.99994038 |
| rs2439823 | Usual walking pace | 459915 | 0.545578 | 0.00127453 | 0.864348434 | 3.52E-05 | 16.17301384 |
| rs2602731 | Usual walking pace | 459915 | 0.681058 | 0.00136907 | 0.928462657 | 2.96E-05 | 13.62410807 |
| rs273512 | Usual walking pace | 459915 | 0.405352 | 0.00129058 | 0.875233068 | 5.88E-05 | 27.03871623 |
| rs35711462 | Usual walking pace | 459915 | 0.511213 | 0.00126947 | 0.860916892 | 3.46E-05 | 15.92433416 |
| rs4109292 | Usual walking pace | 459915 | 0.493943 | 0.00126787 | 0.85983182 | 3.66E-05 | 16.81676532 |
| rs1149620 | All OA | 826690 | 0.439 | 0.004719937 | 4.291484301 | 1.86E-05 | 15.40990295 |
| rs11726937 | All OA | 826690 | 0.2446 | 0.005298546 | 4.817570091 | 1.56E-05 | 12.89568536 |
| rs11749736 | All OA | 826690 | 0.7411 | 0.005175771 | 4.705939965 | 1.49E-05 | 12.29785673 |
| rs2061027 | All OA | 826690 | 0.4999 | 0.004539931 | 4.127818843 | 1.94E-05 | 16.02304404 |
| rs2460451 | All OA | 826690 | 0.4403 | 0.004624563 | 4.204768123 | 1.81E-05 | 14.98579381 |
| rs2820444 | All OA | 826690 | 0.3021 | 0.004928033 | 4.480690576 | 1.65E-05 | 13.61284374 |
| rs3731695 | All OA | 826690 | 0.4546 | 0.004560053 | 4.146114118 | 1.92E-05 | 15.87381967 |
| rs4098282 | All OA | 826690 | 0.8465 | 0.006296114 | 5.724584037 | 1.29E-05 | 10.70009054 |
| rs4858241 | All OA | 826690 | 0.621 | 0.004693031 | 4.267021013 | 1.87E-05 | 15.46555682 |
| rs6942634 | All OA | 826690 | 0.5269 | 0.004577374 | 4.161863035 | 2.13E-05 | 17.6045231 |
| rs696618 | All OA | 826690 | 0.6151 | 0.005558538 | 5.053961231 | 1.77E-05 | 14.632755 |
| rs9445214 | All OA | 826690 | 0.5308 | 0.004556002 | 4.142430792 | 1.81E-05 | 14.99815787 |
| rs10831476 | All OA | 826690 | 0.8107 | 0.005785071 | 5.25993053 | 1.24E-05 | 10.2310618 |
| rs11730727 | All OA | 826690 | 0.6411 | 0.004744057 | 4.3134153 | 2.08E-05 | 17.19623395 |
| rs1467246 | All OA | 826690 | 0.4926 | 0.004544175 | 4.131676843 | 1.92E-05 | 15.86543824 |
| rs1913707 | All OA | 826690 | 0.6062 | 0.0046296 | 4.209348 | 2.90E-05 | 23.96590797 |
| rs1975163 | All OA | 826690 | 0.3188 | 0.00488279 | 4.439554383 | 2.20E-05 | 18.19154322 |
| rs216175 | All OA | 826690 | 0.8278 | 0.006050977 | 5.501699163 | 1.69E-05 | 13.93235635 |
| rs2856821 | All OA | 826690 | 0.7884 | 0.005860523 | 5.328534027 | 1.21E-05 | 10.00999233 |
| rs4979340 | All OA | 826690 | 0.728 | 0.005214992 | 4.741601001 | 1.71E-05 | 14.17550487 |
| rs62262106 | All OA | 826690 | 0.7518 | 0.005280092 | 4.800791147 | 1.42E-05 | 11.72842474 |
| rs8050136 | All OA | 826690 | 0.402 | 0.004625194 | 4.205341568 | 1.80E-05 | 14.84466356 |
| rs1498507 | All OA | 826690 | 0.5344 | 0.004643267 | 4.221773988 | 2.22E-05 | 18.35561544 |
| rs3771501 | All OA | 826690 | 0.4679 | 0.004545783 | 4.13313886 | 3.71E-05 | 30.71217027 |
| rs981819 | All OA | 826690 | 0.3721 | 0.004688744 | 4.26312305 | 2.78E-05 | 23.00752637 |
| rs12939002 | Hand OA | 303782 | 0.7299 | 0.013656988 | 7.527242983 | 3.86E-05 | 11.73368893 |
| rs28538668 | Hand OA | 303782 | 0.7246 | 0.013490727 | 7.435606155 | 4.09E-05 | 12.43444516 |
| rs3993110 | Hand OA | 303782 | 0.6075 | 0.01244414 | 6.858764997 | 6.87E-05 | 20.85992596 |
| rs6511707 | Hand OA | 303782 | 0.6712 | 0.012943329 | 7.13390009 | 4.56E-05 | 13.8488814 |
| rs7294636 | Hand OA | 303782 | 0.3716 | 0.012491174 | 6.884688044 | 4.91E-05 | 14.91980866 |
| rs7748189 | Hand OA | 303782 | 0.7315 | 0.013744905 | 7.575700132 | 4.37E-05 | 13.27439628 |
| rs11071365 | Hand OA | 303782 | 0.3847 | 0.012430245 | 6.85110649 | 1.08E-04 | 32.88844693 |
| rs10465114 | Hip OA | 353388 | 0.2203 | 0.010873718 | 6.46403907 | 3.21E-05 | 11.34977898 |
| rs10831477 | Hip OA | 353388 | 0.8137 | 0.011579246 | 6.883450076 | 3.17E-05 | 11.2073585 |
| rs10940168 | Hip OA | 353388 | 0.3942 | 0.00924821 | 5.497732372 | 4.51E-05 | 15.92429151 |
| rs11049206 | Hip OA | 353388 | 0.1985 | 0.011272101 | 6.700863306 | 9.26E-05 | 32.72009279 |
| rs12046389 | Hip OA | 353388 | 0.4196 | 0.009121927 | 5.422661648 | 9.82E-05 | 34.70893666 |
| rs12074699 | Hip OA | 353388 | 0.3269 | 0.009599077 | 5.706310073 | 5.31E-05 | 18.77678658 |
| rs12209223 | Hip OA | 353388 | 0.1117 | 0.014341135 | 8.525294928 | 5.34E-05 | 18.85866151 |
| rs2716212 | Hip OA | 353388 | 0.6099 | 0.009218742 | 5.480214446 | 4.86E-05 | 17.18539276 |
| rs4148949 | Hip OA | 353388 | 0.3913 | 0.00920359 | 5.471207436 | 4.66E-05 | 16.46041886 |
| rs62578126 | Hip OA | 353388 | 0.3619 | 0.009493962 | 5.643822782 | 5.68E-05 | 20.08090452 |
| rs6792369 | Hip OA | 353388 | 0.6175 | 0.009255941 | 5.502328234 | 7.11E-05 | 25.1242537 |
| rs67924081 | Hip OA | 353388 | 0.7441 | 0.010440953 | 6.20677583 | 4.07E-05 | 14.3991401 |
| rs6908606 | Hip OA | 353388 | 0.711 | 0.009910487 | 5.891432061 | 5.60E-05 | 19.80643206 |
| rs76622165 | Hip OA | 353388 | 0.2225 | 0.011032085 | 6.558182271 | 2.97E-05 | 10.50903544 |
| rs7875152 | Hip OA | 353388 | 0.1408 | 0.013128878 | 7.804651717 | 3.47E-05 | 12.27177848 |
| rs798756 | Hip OA | 353388 | 0.1936 | 0.011422327 | 6.790167489 | 3.16E-05 | 11.16424508 |
| rs8135498 | Hip OA | 353388 | 0.7056 | 0.010055302 | 5.977519994 | 4.43E-05 | 15.64311331 |
| rs9835230 | Hip OA | 353388 | 0.2433 | 0.010523458 | 6.255821571 | 3.83E-05 | 13.53425835 |
| rs10808583 | Hip OA | 353388 | 0.1969 | 0.011369174 | 6.758569654 | 4.67E-05 | 16.4926967 |
| rs1407243 | Hip OA | 353388 | 0.4005 | 0.009157762 | 5.443963805 | 8.61E-05 | 30.43217834 |
| rs1926872 | Hip OA | 353388 | 0.6496 | 0.009410334 | 5.594109184 | 7.27E-05 | 25.69792045 |
| rs1982499 | Hip OA | 353388 | 0.6612 | 0.009463884 | 5.625942813 | 5.71E-05 | 20.17145359 |
| rs4073717 | Hip OA | 353388 | 0.2013 | 0.011276711 | 6.703603999 | 3.23E-05 | 11.41938387 |
| rs9940278 | Hip OA | 353388 | 0.4363 | 0.009023501 | 5.364150606 | 5.04E-05 | 17.81280066 |
| rs10842226 | Knee OA | 396054 | 0.4208 | 0.007914888 | 4.981062098 | 4.28E-05 | 16.97051213 |
| rs10974438 | Knee OA | 396054 | 0.6434 | 0.007417481 | 4.668030062 | 3.97E-05 | 15.70992092 |
| rs11057203 | Knee OA | 396054 | 0.7327 | 0.011299633 | 7.111177535 | 3.43E-05 | 13.56695803 |
| rs11704274 | Knee OA | 396054 | 0.7645 | 0.008642042 | 5.438680844 | 3.19E-05 | 12.63913175 |
| rs1563350 | Knee OA | 396054 | 0.6826 | 0.007551968 | 4.752666802 | 3.78E-05 | 14.97831781 |
| rs2066928 | Knee OA | 396054 | 0.4825 | 0.007228237 | 4.54893357 | 4.10E-05 | 16.224933 |
| rs34195470 | Knee OA | 396054 | 0.4453 | 0.007175423 | 4.515696117 | 6.63E-05 | 26.24676654 |
| rs3823277 | Knee OA | 396054 | 0.3696 | 0.007286507 | 4.585604358 | 3.51E-05 | 13.90332261 |
| rs4380013 | Knee OA | 396054 | 0.1875 | 0.009166453 | 5.768707723 | 2.89E-05 | 11.45341421 |
| rs4912848 | Knee OA | 396054 | 0.2725 | 0.007900198 | 4.971817571 | 3.29E-05 | 13.03653095 |
| rs58973023 | Knee OA | 396054 | 0.4894 | 0.008910928 | 5.607898143 | 4.90E-05 | 19.38804299 |
| rs72979233 | Knee OA | 396054 | 0.7583 | 0.008323737 | 5.238362256 | 3.29E-05 | 13.01626799 |
| rs737142 | Knee OA | 396054 | 0.6635 | 0.00744636 | 4.686204701 | 4.55E-05 | 18.01804762 |
| rs7967762 | Knee OA | 396054 | 0.1718 | 0.009514204 | 5.987556841 | 2.58E-05 | 10.21414938 |
| rs11629600 | Knee OA | 396054 | 0.3411 | 0.007545995 | 4.748907443 | 3.72E-05 | 14.73260929 |
| rs3843750 | Knee OA | 396054 | 0.3173 | 0.007598615 | 4.782022918 | 3.58E-05 | 14.19884305 |
| rs4523957 | Knee OA | 396054 | 0.6299 | 0.007302331 | 4.59556268 | 5.56E-05 | 22.03571309 |
| rs143384 | Knee OA | 396054 | 0.5908 | 0.007190905 | 4.525439284 | 1.23E-04 | 48.74888435 |

Abbreviations: EAF: minor allele frequency; SE: standard error; SD: standard deviation MR: Mendelian randomization；OA: osteoarthritis.
