# Supplementary material for: Genetic causality and site-specific relationship between sarcopenia and osteoarthritis: a bidirectional Mendelian randomization study
Source: Front Genet. 2024 Jan 8;14:1340245. doi: 10.3389/fgene.2023.1340245 (PMC10804883; doi:10.3389/fgene.2023.1340245)
Supplement: Supplementary file 2 [file Table2.DOCX]

**Supplementary Table 2. IVs of sarcopenia-related traits for MR analyses of sarcopenia-related traits on osteoarthritis.**

| **Exposure** | **Outcome** | **SNP** | **Effect allele** | **Other allele** | **GWAS Beta** | | **GWAS P** | |
| --- | --- | --- | --- | --- | --- | --- | --- | --- |
|  |  |  |  |  | **Exposure** | **Outcome** | **Exposure** | **Outcome** |
| **Appendicular lean mass** | **All OA** | rs10112506 | G | A | -0.012 | -0.0102 | 5.76E-10 | 0.03014 |
| **Appendicular lean mass** | **All OA** | rs10471339 | G | C | -0.011 | 6.00E-04 | 1.45E-08 | 0.8997 |
| **Appendicular lean mass** | **All OA** | rs1063582 | G | T | -0.0185 | -0.0026 | 1.12E-16 | 0.6312 |
| **Appendicular lean mass** | **All OA** | rs10776560 | T | C | -0.0157 | -2.00E-04 | 7.88E-17 | 0.959 |
| **Appendicular lean mass** | **All OA** | rs10822117 | G | A | -0.0176 | 0.011 | 4.24E-15 | 0.03874 |
| **Appendicular lean mass** | **All OA** | rs10845408 | T | C | 0.0255 | 0.0092 | 3.25E-38 | 0.05274 |
| **Appendicular lean mass** | **All OA** | rs10858246 | C | G | -0.0188 | 0.0107 | 2.35E-20 | 0.02855 |
| **Appendicular lean mass** | **All OA** | rs10864899 | G | A | -0.0112 | -0.0037 | 3.85E-09 | 0.4804 |
| **Appendicular lean mass** | **All OA** | rs11014285 | A | G | 0.0342 | 0.0062 | 2.89E-40 | 0.3252 |
| **Appendicular lean mass** | **All OA** | rs11121615 | T | C | -0.0202 | 0.0121 | 3.32E-23 | 0.01939 |
| **Appendicular lean mass** | **All OA** | rs111365325 | T | C | -0.0271 | -0.0146 | 1.12E-33 | 0.007204 |
| **Appendicular lean mass** | **All OA** | rs11178643 | T | A | 0.0109 | 3.00E-04 | 4.44E-08 | 0.9581 |
| **Appendicular lean mass** | **All OA** | rs11191208 | A | G | 0.0147 | 0.0108 | 3.69E-10 | 0.06174 |
| **Appendicular lean mass** | **All OA** | rs11198591 | A | G | 0.0148 | 0.0026 | 4.91E-14 | 0.5943 |
| **Appendicular lean mass** | **All OA** | rs11210892 | A | G | 0.0118 | 0.001 | 3.57E-09 | 0.8385 |
| **Appendicular lean mass** | **All OA** | rs11217863 | A | G | -0.0268 | 0.0068 | 1.06E-19 | 0.3305 |
| **Appendicular lean mass** | **All OA** | rs112537273 | C | T | -0.0212 | 0.0052 | 3.34E-21 | 0.3317 |
| **Appendicular lean mass** | **All OA** | rs11260035 | A | G | 0.015 | 0.006 | 1.86E-12 | 0.2399 |
| **Appendicular lean mass** | **All OA** | rs113671109 | C | T | -0.015 | -0.0052 | 4.23E-11 | 0.3445 |
| **Appendicular lean mass** | **All OA** | rs115010283 | C | G | 0.034 | -0.0121 | 2.35E-63 | 0.04372 |
| **Appendicular lean mass** | **All OA** | rs11562101 | T | A | 0.0115 | 0.0033 | 9.39E-09 | 0.5836 |
| **Appendicular lean mass** | **All OA** | rs11580040 | G | A | 0.0325 | 0.0082 | 6.76E-21 | 0.336 |
| **Appendicular lean mass** | **All OA** | rs11590254 | T | A | 0.0186 | 0.0115 | 4.34E-20 | 0.01888 |
| **Appendicular lean mass** | **All OA** | rs115912456 | G | A | 0.0577 | -0.0243 | 3.69E-34 | 0.03281 |
| **Appendicular lean mass** | **All OA** | rs11605297 | A | G | 0.0146 | -0.0034 | 8.03E-11 | 0.5257 |
| **Appendicular lean mass** | **All OA** | rs11612462 | G | T | 0.015 | 0.0059 | 2.55E-09 | 0.335 |
| **Appendicular lean mass** | **All OA** | rs11629593 | G | T | -0.0109 | -1.00E-04 | 4.42E-08 | 0.9925 |
| **Appendicular lean mass** | **All OA** | rs11672848 | T | C | -0.0171 | 0.0043 | 7.73E-19 | 0.4021 |
| **Appendicular lean mass** | **All OA** | rs11721522 | G | A | 0.0106 | -0.0031 | 4.03E-08 | 0.5038 |
| **Appendicular lean mass** | **All OA** | rs11727162 | T | C | -0.017 | 0.0024 | 2.15E-19 | 0.6015 |
| **Appendicular lean mass** | **All OA** | rs11867855 | T | G | -0.0132 | 4.00E-04 | 2.93E-09 | 0.9447 |
| **Appendicular lean mass** | **All OA** | rs12051245 | C | T | 0.0299 | -0.0054 | 2.56E-40 | 0.3135 |
| **Appendicular lean mass** | **All OA** | rs12150907 | A | G | -0.0219 | 0.0028 | 7.22E-20 | 0.6228 |
| **Appendicular lean mass** | **All OA** | rs12188208 | C | A | -0.0195 | -0.0129 | 1.40E-18 | 0.01755 |
| **Appendicular lean mass** | **All OA** | rs12517711 | C | T | -0.0147 | -0.0096 | 2.79E-14 | 0.04443 |
| **Appendicular lean mass** | **All OA** | rs12541381 | A | G | -0.0319 | -6.00E-04 | 2.81E-49 | 0.9163 |
| **Appendicular lean mass** | **All OA** | rs12563442 | C | T | 0.0122 | -0.0089 | 9.93E-09 | 0.08385 |
| **Appendicular lean mass** | **All OA** | rs12655296 | T | C | -0.011 | -0.0037 | 1.62E-08 | 0.4267 |
| **Appendicular lean mass** | **All OA** | rs12702693 | T | C | 0.0173 | -0.0042 | 6.56E-20 | 0.3605 |
| **Appendicular lean mass** | **All OA** | rs12724708 | T | A | 0.0243 | -0.0102 | 1.66E-35 | 0.04544 |
| **Appendicular lean mass** | **All OA** | rs1290786 | T | C | -0.0143 | -0.0039 | 7.14E-14 | 0.3911 |
| **Appendicular lean mass** | **All OA** | rs12943867 | A | G | 0.0184 | -0.001 | 7.57E-20 | 0.8523 |
| **Appendicular lean mass** | **All OA** | rs13391980 | A | G | -0.0225 | -0.0117 | 7.60E-15 | 0.09083 |
| **Appendicular lean mass** | **All OA** | rs143554698 | T | C | -0.0257 | -0.0302 | 3.39E-21 | 4.52E-05 |
| **Appendicular lean mass** | **All OA** | rs1472852 | A | C | -0.0638 | -9.00E-04 | 8.22E-135 | 0.8903 |
| **Appendicular lean mass** | **All OA** | rs14976 | T | C | 0.0144 | -0.0015 | 1.43E-12 | 0.7706 |
| **Appendicular lean mass** | **All OA** | rs1514134 | C | T | -0.0114 | 0.0019 | 3.57E-09 | 0.6859 |
| **Appendicular lean mass** | **All OA** | rs1556659 | T | C | 0.0163 | 0.0092 | 7.19E-17 | 0.05524 |
| **Appendicular lean mass** | **All OA** | rs17197114 | C | T | 0.0177 | 0.0079 | 1.54E-12 | 0.1994 |
| **Appendicular lean mass** | **All OA** | rs17278379 | C | T | 0.0226 | 0.0142 | 2.40E-15 | 0.04174 |
| **Appendicular lean mass** | **All OA** | rs173135 | T | C | -0.0341 | -0.0053 | 3.25E-30 | 0.4605 |
| **Appendicular lean mass** | **All OA** | rs17478946 | G | A | -0.0192 | -0.0054 | 9.98E-21 | 0.2821 |
| **Appendicular lean mass** | **All OA** | rs1880318 | A | G | 0.0147 | -0.0058 | 6.86E-10 | 0.3122 |
| **Appendicular lean mass** | **All OA** | rs2070598 | A | G | 0.0204 | 0.0016 | 6.36E-27 | 0.7171 |
| **Appendicular lean mass** | **All OA** | rs2071450 | T | C | -0.0174 | -0.0051 | 8.85E-19 | 0.2751 |
| **Appendicular lean mass** | **All OA** | rs2089111 | G | C | -0.0172 | -0.0081 | 1.73E-15 | 0.1245 |
| **Appendicular lean mass** | **All OA** | rs2101017 | T | C | -0.0223 | -0.0037 | 1.44E-15 | 0.5713 |
| **Appendicular lean mass** | **All OA** | rs2142331 | T | C | -0.0165 | -0.0091 | 1.38E-17 | 0.05159 |
| **Appendicular lean mass** | **All OA** | rs2188805 | C | A | 0.0114 | 0.0056 | 2.00E-08 | 0.2514 |
| **Appendicular lean mass** | **All OA** | rs2209098 | C | T | 0.024 | 0.0057 | 1.73E-32 | 0.2415 |
| **Appendicular lean mass** | **All OA** | rs2230033 | A | G | -0.0265 | 0.0015 | 3.49E-43 | 0.7473 |
| **Appendicular lean mass** | **All OA** | rs2236096 | C | T | 0.018 | 0.0109 | 1.29E-15 | 0.08619 |
| **Appendicular lean mass** | **All OA** | rs2237485 | A | G | 0.0191 | -0.0036 | 3.73E-17 | 0.5037 |
| **Appendicular lean mass** | **All OA** | rs2268718 | T | C | 0.0141 | 0.0095 | 3.24E-11 | 0.06025 |
| **Appendicular lean mass** | **All OA** | rs2289629 | A | G | -0.0148 | -1.00E-04 | 8.02E-14 | 0.9905 |
| **Appendicular lean mass** | **All OA** | rs2347808 | A | G | -0.0125 | -0.0064 | 5.79E-11 | 0.1597 |
| **Appendicular lean mass** | **All OA** | rs244711 | T | C | 0.0279 | 0.0037 | 1.54E-37 | 0.4973 |
| **Appendicular lean mass** | **All OA** | rs2569888 | A | G | 0.0133 | -0.0092 | 2.29E-09 | 0.08821 |
| **Appendicular lean mass** | **All OA** | rs2578565 | T | C | -0.0141 | -0.0049 | 1.37E-12 | 0.3028 |
| **Appendicular lean mass** | **All OA** | rs2592208 | A | C | -0.0124 | 9.00E-04 | 5.52E-11 | 0.8477 |
| **Appendicular lean mass** | **All OA** | rs2648725 | A | T | 0.0165 | 0.0042 | 8.20E-13 | 0.4736 |
| **Appendicular lean mass** | **All OA** | rs2663126 | A | G | -0.0139 | -3.00E-04 | 1.36E-11 | 0.9442 |
| **Appendicular lean mass** | **All OA** | rs2754255 | G | A | -0.0153 | 2.00E-04 | 1.05E-11 | 0.9697 |
| **Appendicular lean mass** | **All OA** | rs2763263 | A | T | -0.017 | 0.0078 | 1.37E-14 | 0.1363 |
| **Appendicular lean mass** | **All OA** | rs2788213 | A | G | 0.0123 | 0.0012 | 3.80E-09 | 0.8078 |
| **Appendicular lean mass** | **All OA** | rs2789365 | T | C | -0.0145 | -0.0045 | 1.14E-14 | 0.3166 |
| **Appendicular lean mass** | **All OA** | rs28379706 | C | T | 0.0114 | 0.0103 | 4.49E-09 | 0.02756 |
| **Appendicular lean mass** | **All OA** | rs28485212 | T | C | -0.0188 | 0.0018 | 1.24E-12 | 0.7994 |
| **Appendicular lean mass** | **All OA** | rs28529055 | T | G | -0.0147 | 0.0049 | 1.88E-14 | 0.355 |
| **Appendicular lean mass** | **All OA** | rs28529426 | T | C | -0.0168 | -0.0148 | 5.82E-11 | 0.01735 |
| **Appendicular lean mass** | **All OA** | rs28678024 | G | A | -0.0119 | -0.0107 | 1.90E-08 | 0.03487 |
| **Appendicular lean mass** | **All OA** | rs2871960 | C | A | 0.0469 | 0.0012 | 2.17E-135 | 0.7839 |
| **Appendicular lean mass** | **All OA** | rs2923411 | C | T | 0.0127 | 0.0114 | 4.77E-11 | 0.01357 |
| **Appendicular lean mass** | **All OA** | rs2925155 | T | C | -0.015 | -0.0105 | 5.47E-12 | 0.04757 |
| **Appendicular lean mass** | **All OA** | rs2978362 | T | C | 0.0106 | -0.0033 | 2.85E-08 | 0.478 |
| **Appendicular lean mass** | **All OA** | rs3116194 | A | T | -0.0295 | 0.0175 | 8.29E-21 | 0.02489 |
| **Appendicular lean mass** | **All OA** | rs331917 | G | A | -0.0127 | 0.001 | 3.54E-11 | 0.8275 |
| **Appendicular lean mass** | **All OA** | rs336630 | T | C | -0.0106 | -0.0057 | 2.90E-08 | 0.2288 |
| **Appendicular lean mass** | **All OA** | rs34312629 | G | C | -0.017 | -0.002 | 2.12E-15 | 0.7007 |
| **Appendicular lean mass** | **All OA** | rs34338597 | G | A | -0.0112 | -0.0052 | 7.88E-09 | 0.2649 |
| **Appendicular lean mass** | **All OA** | rs34345560 | A | G | 0.0219 | 0.0021 | 7.10E-20 | 0.7165 |
| **Appendicular lean mass** | **All OA** | rs34517439 | A | C | 0.0421 | 0.0193 | 5.80E-48 | 0.006828 |
| **Appendicular lean mass** | **All OA** | rs35073631 | C | T | 0.0112 | 6.00E-04 | 5.92E-09 | 0.9039 |
| **Appendicular lean mass** | **All OA** | rs35288270 | C | T | -0.0328 | 0.0142 | 3.43E-32 | 0.05657 |
| **Appendicular lean mass** | **All OA** | rs35732917 | C | T | 0.0204 | 0.0031 | 2.08E-22 | 0.5369 |
| **Appendicular lean mass** | **All OA** | rs35756741 | T | C | -0.0378 | 8.00E-04 | 5.80E-31 | 0.9127 |
| **Appendicular lean mass** | **All OA** | rs35811052 | G | A | -0.0148 | 0.0101 | 8.84E-12 | 0.05422 |
| **Appendicular lean mass** | **All OA** | rs36000545 | G | A | -0.022 | -0.0091 | 2.56E-29 | 0.05426 |
| **Appendicular lean mass** | **All OA** | rs36048468 | T | C | 0.0254 | 0.0045 | 9.34E-28 | 0.428 |
| **Appendicular lean mass** | **All OA** | rs36226649 | C | T | 0.0485 | 0.0033 | 3.05E-37 | 0.7205 |
| **Appendicular lean mass** | **All OA** | rs3782232 | A | G | -0.0339 | -0.0116 | 2.41E-20 | 0.1995 |
| **Appendicular lean mass** | **All OA** | rs3828729 | G | A | -0.016 | -0.0088 | 4.67E-15 | 0.07586 |
| **Appendicular lean mass** | **All OA** | rs40270 | C | A | 0.0151 | -3.00E-04 | 1.90E-11 | 0.9573 |
| **Appendicular lean mass** | **All OA** | rs4282339 | A | G | -0.0311 | -7.00E-04 | 6.16E-41 | 0.8944 |
| **Appendicular lean mass** | **All OA** | rs4287835 | C | T | 0.0147 | -0.0066 | 9.94E-15 | 0.1499 |
| **Appendicular lean mass** | **All OA** | rs4360494 | C | G | -0.0198 | 0.0012 | 7.88E-26 | 0.7992 |
| **Appendicular lean mass** | **All OA** | rs45474992 | T | C | -0.0617 | -0.019 | 2.17E-33 | 0.1248 |
| **Appendicular lean mass** | **All OA** | rs45528934 | T | C | 0.0262 | -0.0052 | 1.97E-24 | 0.4139 |
| **Appendicular lean mass** | **All OA** | rs4640244 | G | A | -0.02 | 0.0034 | 3.81E-25 | 0.4584 |
| **Appendicular lean mass** | **All OA** | rs4682483 | A | G | -0.0165 | -8.00E-04 | 2.65E-10 | 0.8973 |
| **Appendicular lean mass** | **All OA** | rs4735761 | C | A | 0.0331 | 0.0111 | 3.66E-56 | 0.02715 |
| **Appendicular lean mass** | **All OA** | rs4752689 | A | G | 0.0205 | -0.0053 | 1.36E-26 | 0.2594 |
| **Appendicular lean mass** | **All OA** | rs4847378 | T | G | 0.0136 | -0.0069 | 1.64E-12 | 0.1413 |
| **Appendicular lean mass** | **All OA** | rs4870941 | C | G | -0.0297 | -0.0032 | 1.09E-39 | 0.5487 |
| **Appendicular lean mass** | **All OA** | rs4932439 | G | A | -0.0151 | -9.00E-04 | 1.43E-09 | 0.8842 |
| **Appendicular lean mass** | **All OA** | rs496783 | G | A | -0.0124 | -6.00E-04 | 8.13E-11 | 0.8968 |
| **Appendicular lean mass** | **All OA** | rs4976262 | C | T | -0.0245 | 0.0095 | 4.37E-33 | 0.0493 |
| **Appendicular lean mass** | **All OA** | rs55758152 | A | G | 0.0145 | -8.00E-04 | 1.05E-12 | 0.8835 |
| **Appendicular lean mass** | **All OA** | rs55852614 | C | T | -0.0393 | 9.00E-04 | 3.29E-73 | 0.8607 |
| **Appendicular lean mass** | **All OA** | rs56112295 | T | C | 0.0154 | 0.0121 | 1.12E-10 | 0.05786 |
| **Appendicular lean mass** | **All OA** | rs568267 | T | C | 0.0122 | 0.0088 | 2.23E-08 | 0.09215 |
| **Appendicular lean mass** | **All OA** | rs57059662 | C | T | 0.0118 | -0.0043 | 5.63E-09 | 0.3725 |
| **Appendicular lean mass** | **All OA** | rs57513571 | T | C | -0.0191 | -0.0191 | 7.02E-16 | 8.49E-04 |
| **Appendicular lean mass** | **All OA** | rs591668 | A | G | -0.0174 | -0.0026 | 2.00E-19 | 0.5742 |
| **Appendicular lean mass** | **All OA** | rs599004 | T | C | -0.0157 | -0.0101 | 6.53E-14 | 0.04819 |
| **Appendicular lean mass** | **All OA** | rs59950280 | A | G | -0.0254 | -0.0081 | 7.32E-36 | 0.1049 |
| **Appendicular lean mass** | **All OA** | rs604723 | C | T | -0.0166 | -0.0084 | 8.16E-15 | 0.09809 |
| **Appendicular lean mass** | **All OA** | rs6142059 | C | T | 0.0116 | 0.0026 | 1.19E-09 | 0.5705 |
| **Appendicular lean mass** | **All OA** | rs61919240 | A | T | 0.0137 | 0.0037 | 9.77E-12 | 0.4445 |
| **Appendicular lean mass** | **All OA** | rs62033029 | A | G | -0.0141 | 0.0018 | 1.73E-09 | 0.748 |
| **Appendicular lean mass** | **All OA** | rs62143873 | A | G | -0.0115 | 0.0038 | 1.19E-09 | 0.4198 |
| **Appendicular lean mass** | **All OA** | rs62466110 | C | T | -0.0371 | -0.0065 | 5.74E-20 | 0.524 |
| **Appendicular lean mass** | **All OA** | rs62501195 | C | A | -0.0198 | -0.0043 | 8.11E-15 | 0.4811 |
| **Appendicular lean mass** | **All OA** | rs62515437 | T | G | 0.0369 | -0.0018 | 8.79E-60 | 0.7413 |
| **Appendicular lean mass** | **All OA** | rs6582398 | T | C | 0.014 | 0.0087 | 1.14E-12 | 0.0811 |
| **Appendicular lean mass** | **All OA** | rs6675858 | T | C | -0.0137 | -0.0031 | 2.40E-09 | 0.574 |
| **Appendicular lean mass** | **All OA** | rs6738207 | A | G | 0.0127 | 0.0041 | 4.14E-11 | 0.3769 |
| **Appendicular lean mass** | **All OA** | rs67527161 | C | T | -0.0182 | -0.0041 | 5.79E-15 | 0.4616 |
| **Appendicular lean mass** | **All OA** | rs67551338 | T | C | 0.0576 | -4.00E-04 | 1.04E-47 | 0.9651 |
| **Appendicular lean mass** | **All OA** | rs67716382 | C | G | 0.0226 | -0.0079 | 1.65E-23 | 0.1481 |
| **Appendicular lean mass** | **All OA** | rs6789000 | T | G | 0.0121 | 0.0106 | 1.61E-09 | 0.07336 |
| **Appendicular lean mass** | **All OA** | rs68049170 | A | G | -0.0259 | -0.0034 | 2.69E-34 | 0.5035 |
| **Appendicular lean mass** | **All OA** | rs6821305 | C | A | 0.0204 | 0.0035 | 3.15E-26 | 0.471 |
| **Appendicular lean mass** | **All OA** | rs684905 | T | C | -0.0118 | 0.0072 | 7.10E-10 | 0.1651 |
| **Appendicular lean mass** | **All OA** | rs6849302 | G | A | 0.0155 | -0.0049 | 7.11E-11 | 0.386 |
| **Appendicular lean mass** | **All OA** | rs6874142 | G | T | 0.0288 | 0.0193 | 5.15E-20 | 0.01899 |
| **Appendicular lean mass** | **All OA** | rs700677 | A | C | 0.0173 | 0.0056 | 1.13E-18 | 0.2524 |
| **Appendicular lean mass** | **All OA** | rs7014590 | C | T | -0.0228 | 0.0109 | 4.48E-26 | 0.03689 |
| **Appendicular lean mass** | **All OA** | rs702886 | G | A | 0.012 | 0.0084 | 1.11E-09 | 0.07622 |
| **Appendicular lean mass** | **All OA** | rs71414738 | T | C | 0.015 | 0.0031 | 1.00E-09 | 0.608 |
| **Appendicular lean mass** | **All OA** | rs718603 | T | C | 0.0131 | 0.0012 | 6.68E-10 | 0.8149 |
| **Appendicular lean mass** | **All OA** | rs7229520 | A | G | -0.0224 | -0.0039 | 9.21E-29 | 0.4089 |
| **Appendicular lean mass** | **All OA** | rs72656010 | C | T | -0.0668 | -0.0114 | 7.31E-126 | 0.09104 |
| **Appendicular lean mass** | **All OA** | rs72721979 | G | T | -0.0229 | 0.0068 | 2.20E-17 | 0.3072 |
| **Appendicular lean mass** | **All OA** | rs72801843 | A | T | 0.0313 | 0.0125 | 8.83E-52 | 0.01164 |
| **Appendicular lean mass** | **All OA** | rs72841270 | G | T | 0.0294 | 0.0031 | 2.25E-26 | 0.6391 |
| **Appendicular lean mass** | **All OA** | rs72894003 | T | C | -0.0423 | 0.019 | 1.90E-28 | 0.05593 |
| **Appendicular lean mass** | **All OA** | rs73052033 | C | T | -0.0151 | -0.0178 | 4.79E-10 | 0.002378 |
| **Appendicular lean mass** | **All OA** | rs7320878 | A | G | -0.015 | -0.0043 | 1.34E-14 | 0.4068 |
| **Appendicular lean mass** | **All OA** | rs7328187 | G | T | 0.0116 | 0.0075 | 1.19E-09 | 0.1016 |
| **Appendicular lean mass** | **All OA** | rs73384223 | C | T | -0.0205 | -0.004 | 1.27E-17 | 0.4939 |
| **Appendicular lean mass** | **All OA** | rs73413540 | T | C | -0.0124 | -3.00E-04 | 4.42E-08 | 0.9618 |
| **Appendicular lean mass** | **All OA** | rs73696333 | G | C | 0.0191 | 0.0053 | 3.20E-15 | 0.3784 |
| **Appendicular lean mass** | **All OA** | rs7418410 | T | C | 0.0155 | -0.0114 | 5.64E-16 | 0.01287 |
| **Appendicular lean mass** | **All OA** | rs75022676 | A | G | -0.0163 | -0.0019 | 2.84E-12 | 0.7469 |
| **Appendicular lean mass** | **All OA** | rs75702986 | A | G | -0.0163 | 0.0031 | 3.14E-11 | 0.6013 |
| **Appendicular lean mass** | **All OA** | rs7598430 | T | C | -0.016 | -0.0111 | 1.37E-17 | 0.0148 |
| **Appendicular lean mass** | **All OA** | rs7610055 | A | G | -0.0373 | -0.0016 | 3.55E-38 | 0.8154 |
| **Appendicular lean mass** | **All OA** | rs76364830 | A | G | -0.0471 | -0.0029 | 2.70E-33 | 0.7703 |
| **Appendicular lean mass** | **All OA** | rs7689420 | C | T | 0.0466 | 0.0082 | 1.50E-76 | 0.1807 |
| **Appendicular lean mass** | **All OA** | rs7701233 | C | T | -0.0179 | -0.0035 | 5.12E-21 | 0.4783 |
| **Appendicular lean mass** | **All OA** | rs772222 | G | A | 0.0121 | 0.0052 | 1.55E-08 | 0.3139 |
| **Appendicular lean mass** | **All OA** | rs7731023 | G | A | 0.0166 | 0.0013 | 3.48E-18 | 0.7842 |
| **Appendicular lean mass** | **All OA** | rs7768973 | A | T | -0.024 | -0.0112 | 5.44E-36 | 0.0147 |
| **Appendicular lean mass** | **All OA** | rs7828086 | C | T | 0.0135 | 0.001 | 1.11E-09 | 0.8564 |
| **Appendicular lean mass** | **All OA** | rs78766798 | C | T | 0.0319 | 0.01 | 2.61E-20 | 0.257 |
| **Appendicular lean mass** | **All OA** | rs7971536 | A | T | -0.0194 | -0.0038 | 1.06E-24 | 0.402 |
| **Appendicular lean mass** | **All OA** | rs8017006 | G | A | 0.0122 | -0.0014 | 2.22E-09 | 0.8066 |
| **Appendicular lean mass** | **All OA** | rs8018486 | G | A | -0.0138 | 8.00E-04 | 1.18E-08 | 0.8862 |
| **Appendicular lean mass** | **All OA** | rs8019890 | A | C | 0.025 | -0.0044 | 1.96E-38 | 0.3492 |
| **Appendicular lean mass** | **All OA** | rs80295797 | T | C | -0.0198 | -0.0015 | 3.83E-23 | 0.7535 |
| **Appendicular lean mass** | **All OA** | rs8084413 | A | G | -0.0127 | 0.0055 | 3.25E-11 | 0.2377 |
| **Appendicular lean mass** | **All OA** | rs861674 | T | A | 0.0128 | 0.0147 | 1.36E-11 | 0.001225 |
| **Appendicular lean mass** | **All OA** | rs900399 | G | A | 0.0164 | 0.0026 | 1.35E-17 | 0.5826 |
| **Appendicular lean mass** | **All OA** | rs9266244 | A | G | -0.0427 | -0.0109 | 1.21E-94 | 0.06345 |
| **Appendicular lean mass** | **All OA** | rs9343327 | T | A | 0.014 | 0.0149 | 1.09E-13 | 0.00105 |
| **Appendicular lean mass** | **All OA** | rs9375188 | T | C | 0.0136 | 0.0038 | 6.80E-13 | 0.4014 |
| **Appendicular lean mass** | **All OA** | rs9385002 | T | A | -0.0147 | 4.00E-04 | 3.26E-11 | 0.9452 |
| **Appendicular lean mass** | **All OA** | rs9391254 | T | C | 0.0166 | -0.0081 | 2.10E-16 | 0.1006 |
| **Appendicular lean mass** | **All OA** | rs951366 | C | T | 0.0205 | -0.0068 | 9.15E-27 | 0.1435 |
| **Appendicular lean mass** | **All OA** | rs9568031 | T | C | -0.0115 | 1.00E-04 | 3.34E-08 | 0.9842 |
| **Appendicular lean mass** | **All OA** | rs9636364 | A | G | 0.011 | 0.0081 | 5.09E-09 | 0.07512 |
| **Appendicular lean mass** | **All OA** | rs9809116 | G | A | -0.016 | -0.0052 | 1.31E-16 | 0.2661 |
| **Hand grip strength (left)** | **All OA** | rs10097417 | G | A | -0.0132302 | 0.0077 | 1.90E-11 | 0.203 |
| **Hand grip strength (left)** | **All OA** | rs10176878 | C | T | -0.0129478 | 0.007 | 8.60E-12 | 0.2345 |
| **Hand grip strength (left)** | **All OA** | rs10403906 | A | G | -0.0100323 | 0.0022 | 1.50E-11 | 0.6365 |
| **Hand grip strength (left)** | **All OA** | rs10786706 | T | C | 0.0100072 | -0.001 | 1.70E-11 | 0.8322 |
| **Hand grip strength (left)** | **All OA** | rs10831903 | T | C | 0.00928662 | -0.0076 | 8.10E-10 | 0.1028 |
| **Hand grip strength (left)** | **All OA** | rs10846071 | T | C | -0.016641 | 0.0071 | 5.30E-28 | 0.1281 |
| **Hand grip strength (left)** | **All OA** | rs10934857 | A | G | 0.00928675 | -0.001 | 4.80E-08 | 0.8498 |
| **Hand grip strength (left)** | **All OA** | rs10988217 | G | A | -0.00920657 | 0.0041 | 1.70E-09 | 0.3848 |
| **Hand grip strength (left)** | **All OA** | rs11121542 | A | G | -0.0157601 | -5.00E-04 | 3.00E-12 | 0.9481 |
| **Hand grip strength (left)** | **All OA** | rs11243202 | C | T | 0.00972237 | -0.0121 | 7.00E-11 | 0.00808 |
| **Hand grip strength (left)** | **All OA** | rs11642954 | A | G | -0.0117025 | 0.0011 | 4.50E-10 | 0.8519 |
| **Hand grip strength (left)** | **All OA** | rs12473732 | T | C | 0.0109869 | -0.0011 | 1.40E-13 | 0.8087 |
| **Hand grip strength (left)** | **All OA** | rs12889267 | G | A | -0.0137394 | 0.0035 | 4.70E-12 | 0.5767 |
| **Hand grip strength (left)** | **All OA** | rs12906830 | C | T | 0.0108401 | -0.0012 | 9.30E-13 | 0.7892 |
| **Hand grip strength (left)** | **All OA** | rs13091492 | G | A | -0.00847903 | 0.0029 | 3.30E-08 | 0.5335 |
| **Hand grip strength (left)** | **All OA** | rs13146142 | C | T | -0.0202031 | -8.00E-04 | 2.30E-23 | 0.906 |
| **Hand grip strength (left)** | **All OA** | rs13337177 | T | G | -0.0142799 | 0.0048 | 1.60E-13 | 0.4481 |
| **Hand grip strength (left)** | **All OA** | rs16870531 | T | C | 0.011198 | 0.0055 | 1.30E-10 | 0.3015 |
| **Hand grip strength (left)** | **All OA** | rs17282763 | C | T | 0.00893956 | -0.0021 | 4.40E-08 | 0.6734 |
| **Hand grip strength (left)** | **All OA** | rs1884447 | A | G | 0.00846069 | -0.0071 | 2.30E-08 | 0.1277 |
| **Hand grip strength (left)** | **All OA** | rs2359239 | T | C | -0.00881461 | 0.0065 | 6.90E-09 | 0.164 |
| **Hand grip strength (left)** | **All OA** | rs2871960 | C | A | 0.0120879 | 0.0012 | 5.50E-16 | 0.7839 |
| **Hand grip strength (left)** | **All OA** | rs2974438 | A | G | -0.0100735 | -7.00E-04 | 3.30E-08 | 0.9067 |
| **Hand grip strength (left)** | **All OA** | rs35175534 | C | A | -0.0163733 | 9.00E-04 | 3.60E-12 | 0.924 |
| **Hand grip strength (left)** | **All OA** | rs4121165 | A | G | -0.0114169 | -0.0033 | 3.40E-10 | 0.5567 |
| **Hand grip strength (left)** | **All OA** | rs4575361 | T | A | -0.0108028 | -6.00E-04 | 1.60E-11 | 0.9005 |
| **Hand grip strength (left)** | **All OA** | rs4962700 | G | C | 0.00904307 | 0.0043 | 3.20E-08 | 0.4245 |
| **Hand grip strength (left)** | **All OA** | rs59116179 | T | C | 0.00857385 | -6.00E-04 | 2.40E-08 | 0.9061 |
| **Hand grip strength (left)** | **All OA** | rs61286123 | C | T | -0.0101041 | 0.0033 | 1.20E-08 | 0.5449 |
| **Hand grip strength (left)** | **All OA** | rs62081464 | T | C | -0.0098827 | 0.0086 | 2.80E-08 | 0.113 |
| **Hand grip strength (left)** | **All OA** | rs6689375 | T | A | -0.0160169 | 0.0068 | 5.30E-17 | 0.2464 |
| **Hand grip strength (left)** | **All OA** | rs7197751 | T | G | -0.00946321 | 0.0062 | 1.40E-09 | 0.1969 |
| **Hand grip strength (left)** | **All OA** | rs73307079 | C | T | 0.0111025 | -0.0043 | 1.40E-09 | 0.4314 |
| **Hand grip strength (left)** | **All OA** | rs772014 | G | A | -0.0106178 | -0.0053 | 2.70E-12 | 0.2648 |
| **Hand grip strength (left)** | **All OA** | rs7856625 | T | C | -0.0111297 | -0.0035 | 2.50E-13 | 0.4488 |
| **Hand grip strength (left)** | **All OA** | rs821100 | G | A | -0.0101977 | 0.0111 | 1.50E-09 | 0.03278 |
| **Hand grip strength (left)** | **All OA** | rs9371201 | T | C | -0.00933847 | -0.0048 | 3.00E-09 | 0.3152 |
| **Hand grip strength (left)** | **All OA** | rs9371881 | A | G | 0.00947946 | 0.0043 | 9.60E-10 | 0.3721 |
| **Hand grip strength (left)** | **All OA** | rs9611273 | T | C | 0.0107888 | -9.00E-04 | 4.50E-10 | 0.8727 |
| **Hand grip strength (left)** | **All OA** | rs9944324 | G | A | -0.00855272 | -0.0028 | 1.20E-08 | 0.544 |
| **Hand grip strength (right)** | **All OA** | rs10278546 | C | A | 0.0107539 | -0.0087 | 1.10E-08 | 0.1291 |
| **Hand grip strength (right)** | **All OA** | rs1043515 | G | A | 0.0138281 | -4.00E-04 | 2.80E-20 | 0.9313 |
| **Hand grip strength (right)** | **All OA** | rs10770125 | G | A | 0.00844132 | -0.001 | 1.40E-08 | 0.8234 |
| **Hand grip strength (right)** | **All OA** | rs10799428 | T | C | -0.0143588 | 0.0074 | 4.60E-14 | 0.2019 |
| **Hand grip strength (right)** | **All OA** | rs10846071 | T | C | -0.0156575 | 0.0071 | 6.50E-25 | 0.1281 |
| **Hand grip strength (right)** | **All OA** | rs113315602 | C | A | -0.0212911 | 0.008 | 1.40E-15 | 0.4595 |
| **Hand grip strength (right)** | **All OA** | rs113835839 | T | C | -0.00986353 | 0.0043 | 1.10E-08 | 0.4165 |
| **Hand grip strength (right)** | **All OA** | rs11642954 | A | G | -0.0131918 | 0.0011 | 2.20E-12 | 0.8519 |
| **Hand grip strength (right)** | **All OA** | rs12412806 | A | G | -0.00909582 | -6.00E-04 | 3.00E-08 | 0.902 |
| **Hand grip strength (right)** | **All OA** | rs12452505 | G | C | -0.0144313 | -0.0029 | 1.40E-11 | 0.6731 |
| **Hand grip strength (right)** | **All OA** | rs12823922 | G | A | -0.0114203 | 0.0088 | 1.60E-10 | 0.1084 |
| **Hand grip strength (right)** | **All OA** | rs12889267 | G | A | -0.0122874 | 0.0035 | 6.70E-10 | 0.5767 |
| **Hand grip strength (right)** | **All OA** | rs13146142 | C | T | -0.0207968 | -8.00E-04 | 1.40E-24 | 0.906 |
| **Hand grip strength (right)** | **All OA** | rs13169333 | C | T | 0.00929083 | -0.0107 | 4.30E-08 | 0.03592 |
| **Hand grip strength (right)** | **All OA** | rs1442883 | A | C | -0.0106189 | 0.0016 | 5.80E-10 | 0.7563 |
| **Hand grip strength (right)** | **All OA** | rs1885690 | A | C | -0.00839964 | 0.0101 | 2.80E-08 | 0.03212 |
| **Hand grip strength (right)** | **All OA** | rs2194747 | G | A | 0.00985126 | 0.0023 | 1.90E-09 | 0.6453 |
| **Hand grip strength (right)** | **All OA** | rs2226685 | C | T | 0.0103333 | 0.0014 | 3.10E-09 | 0.7934 |
| **Hand grip strength (right)** | **All OA** | rs248831 | A | G | 0.00988989 | -0.0078 | 8.40E-09 | 0.1415 |
| **Hand grip strength (right)** | **All OA** | rs2854152 | G | A | 0.010991 | 0.0025 | 6.00E-12 | 0.6042 |
| **Hand grip strength (right)** | **All OA** | rs35175534 | C | A | -0.0191822 | 9.00E-04 | 4.20E-16 | 0.924 |
| **Hand grip strength (right)** | **All OA** | rs35833641 | G | A | 0.00922306 | -0.0012 | 8.50E-09 | 0.7992 |
| **Hand grip strength (right)** | **All OA** | rs3848369 | T | C | -0.00953776 | -0.0027 | 4.70E-10 | 0.5657 |
| **Hand grip strength (right)** | **All OA** | rs4121165 | A | G | -0.0119792 | -0.0033 | 4.80E-11 | 0.5567 |
| **Hand grip strength (right)** | **All OA** | rs4549685 | T | C | 0.00971494 | 0.0018 | 8.10E-10 | 0.7145 |
| **Hand grip strength (right)** | **All OA** | rs4751671 | A | G | 0.0082332 | 7.00E-04 | 4.10E-08 | 0.8803 |
| **Hand grip strength (right)** | **All OA** | rs4752689 | A | G | 0.0087531 | -0.0053 | 6.40E-09 | 0.2594 |
| **Hand grip strength (right)** | **All OA** | rs4868110 | T | A | -0.00971069 | -0.0026 | 1.00E-09 | 0.5908 |
| **Hand grip strength (right)** | **All OA** | rs4962700 | G | C | 0.00932733 | 0.0043 | 1.20E-08 | 0.4245 |
| **Hand grip strength (right)** | **All OA** | rs56074046 | A | G | -0.00903502 | 0.0057 | 4.40E-09 | 0.2302 |
| **Hand grip strength (right)** | **All OA** | rs56365901 | G | A | -0.0142618 | 0.0098 | 1.80E-15 | 0.0837 |
| **Hand grip strength (right)** | **All OA** | rs600038 | C | T | -0.0103761 | 0.0045 | 1.50E-08 | 0.4208 |
| **Hand grip strength (right)** | **All OA** | rs62037412 | A | G | 0.00932966 | -0.0043 | 1.90E-09 | 0.3687 |
| **Hand grip strength (right)** | **All OA** | rs6693965 | T | G | -0.016251 | -0.0019 | 3.20E-13 | 0.7788 |
| **Hand grip strength (right)** | **All OA** | rs6792762 | A | G | -0.00911683 | 0.0087 | 1.80E-09 | 0.05998 |
| **Hand grip strength (right)** | **All OA** | rs721101 | C | T | 0.00946697 | 0.003 | 1.60E-08 | 0.5627 |
| **Hand grip strength (right)** | **All OA** | rs7266065 | A | G | 0.00993881 | -0.0021 | 4.60E-10 | 0.6628 |
| **Hand grip strength (right)** | **All OA** | rs7301953 | A | G | -0.0115397 | -8.00E-04 | 6.40E-13 | 0.8716 |
| **Hand grip strength (right)** | **All OA** | rs7790322 | T | C | -0.00860301 | -0.0039 | 1.20E-08 | 0.3964 |
| **Hand grip strength (right)** | **All OA** | rs7871404 | G | A | 0.0119345 | 0.0025 | 3.40E-10 | 0.6658 |
| **Hand grip strength (right)** | **All OA** | rs935728 | T | C | 0.00955337 | 8.00E-04 | 1.80E-09 | 0.8696 |
| **Hand grip strength (right)** | **All OA** | rs9639938 | G | C | 0.00873031 | 7.00E-04 | 5.20E-09 | 0.8715 |
| **Hand grip strength (right)** | **All OA** | rs9652468 | A | G | -0.0125308 | 0.0029 | 3.30E-13 | 0.5758 |
| **Hand grip strength (right)** | **All OA** | rs9757079 | T | C | 0.00980223 | -0.0076 | 8.40E-10 | 0.12 |
| **Hand grip strength (right)** | **All OA** | rs9853018 | T | C | 0.0101977 | -1.00E-04 | 8.80E-12 | 0.9874 |
| **Usual walking pace** | **All OA** | rs10828258 | G | A | -0.00932641 | 0.0167 | 6.70E-12 | 7.01E-04 |
| **Usual walking pace** | **All OA** | rs10862220 | G | T | 0.00840767 | -0.0108 | 4.80E-10 | 0.02445 |
| **Usual walking pace** | **All OA** | rs11039324 | A | G | -0.0102105 | -0.0041 | 2.20E-15 | 0.3774 |
| **Usual walking pace** | **All OA** | rs11150623 | T | G | -0.00968813 | 0.0042 | 2.40E-13 | 0.3808 |
| **Usual walking pace** | **All OA** | rs113825410 | G | A | -0.00881182 | 0.0195 | 6.40E-09 | 3.24E-04 |
| **Usual walking pace** | **All OA** | rs11682482 | G | T | 0.00801894 | 0.0011 | 3.30E-09 | 0.8193 |
| **Usual walking pace** | **All OA** | rs11732213 | C | T | 0.0091614 | -0.0313 | 9.40E-09 | 5.16E-08 |
| **Usual walking pace** | **All OA** | rs11761141 | G | T | -0.00789936 | -0.0064 | 5.00E-09 | 0.1859 |
| **Usual walking pace** | **All OA** | rs11848096 | C | T | -0.00750831 | -7.00E-04 | 9.20E-09 | 0.8884 |
| **Usual walking pace** | **All OA** | rs12461902 | A | G | -0.00801134 | 0.005 | 3.60E-09 | 0.2986 |
| **Usual walking pace** | **All OA** | rs205262 | G | A | -0.00870949 | 0.0237 | 1.00E-09 | 3.92E-06 |
| **Usual walking pace** | **All OA** | rs2170670 | A | G | -0.00708769 | 0.0076 | 4.70E-08 | 0.1005 |
| **Usual walking pace** | **All OA** | rs2280406 | A | G | -0.00996145 | 0.0206 | 3.40E-15 | 5.64E-06 |
| **Usual walking pace** | **All OA** | rs2439823 | G | A | -0.0072789 | 0.0036 | 1.10E-08 | 0.4394 |
| **Usual walking pace** | **All OA** | rs2602731 | G | A | -0.00766675 | 0.0104 | 2.10E-08 | 0.03564 |
| **Usual walking pace** | **All OA** | rs273512 | T | C | -0.00966507 | 0.0089 | 6.90E-14 | 0.05375 |
| **Usual walking pace** | **All OA** | rs35711462 | G | A | -0.0071659 | 0.0105 | 1.70E-08 | 0.02038 |
| **Usual walking pace** | **All OA** | rs4109292 | A | G | 0.00735336 | -0.0112 | 6.60E-09 | 0.0153 |
| **Appendicular lean mass** | **Hand OA** | rs10112506 | G | A | -0.012 | -0.0183 | 5.76E-10 | 0.1449 |
| **Appendicular lean mass** | **Hand OA** | rs10203320 | C | T | 0.0138 | 0.0143 | 7.86E-12 | 0.2698 |
| **Appendicular lean mass** | **Hand OA** | rs10471339 | G | C | -0.011 | 0.013 | 1.45E-08 | 0.3025 |
| **Appendicular lean mass** | **Hand OA** | rs1047891 | A | C | 0.0233 | 0.0213 | 5.70E-31 | 0.1692 |
| **Appendicular lean mass** | **Hand OA** | rs1063582 | G | T | -0.0185 | 0.0174 | 1.12E-16 | 0.2198 |
| **Appendicular lean mass** | **Hand OA** | rs10776560 | T | C | -0.0157 | -0.0309 | 7.88E-17 | 0.01079 |
| **Appendicular lean mass** | **Hand OA** | rs10822117 | G | A | -0.0176 | 0.0031 | 4.24E-15 | 0.8287 |
| **Appendicular lean mass** | **Hand OA** | rs10845408 | T | C | 0.0255 | 0.0241 | 3.25E-38 | 0.05618 |
| **Appendicular lean mass** | **Hand OA** | rs10858246 | C | G | -0.0188 | 0.0123 | 2.35E-20 | 0.3468 |
| **Appendicular lean mass** | **Hand OA** | rs10864899 | G | A | -0.0112 | -0.0096 | 3.85E-09 | 0.5607 |
| **Appendicular lean mass** | **Hand OA** | rs11014285 | A | G | 0.0342 | 0.0208 | 2.89E-40 | 0.2227 |
| **Appendicular lean mass** | **Hand OA** | rs11121615 | T | C | -0.0202 | 0.0105 | 3.32E-23 | 0.4895 |
| **Appendicular lean mass** | **Hand OA** | rs111365325 | T | C | -0.0271 | -0.0253 | 1.12E-33 | 0.08264 |
| **Appendicular lean mass** | **Hand OA** | rs11178643 | T | A | 0.0109 | 0.0313 | 4.44E-08 | 0.1147 |
| **Appendicular lean mass** | **Hand OA** | rs11191208 | A | G | 0.0147 | 0.0021 | 3.69E-10 | 0.8953 |
| **Appendicular lean mass** | **Hand OA** | rs11198591 | A | G | 0.0148 | 0.0034 | 4.91E-14 | 0.7981 |
| **Appendicular lean mass** | **Hand OA** | rs11210892 | A | G | 0.0118 | 0.0106 | 3.57E-09 | 0.413 |
| **Appendicular lean mass** | **Hand OA** | rs11217863 | A | G | -0.0268 | 0.0256 | 1.06E-19 | 0.1706 |
| **Appendicular lean mass** | **Hand OA** | rs11243202 | C | T | 0.0302 | -0.0108 | 2.83E-57 | 0.3738 |
| **Appendicular lean mass** | **Hand OA** | rs112537273 | C | T | -0.0212 | 0.0025 | 3.34E-21 | 0.8634 |
| **Appendicular lean mass** | **Hand OA** | rs11260035 | A | G | 0.015 | 0.018 | 1.86E-12 | 0.1897 |
| **Appendicular lean mass** | **Hand OA** | rs113671109 | C | T | -0.015 | -0.019 | 4.23E-11 | 0.1962 |
| **Appendicular lean mass** | **Hand OA** | rs115010283 | C | G | 0.034 | -0.0034 | 2.35E-63 | 0.8608 |
| **Appendicular lean mass** | **Hand OA** | rs11562101 | T | A | 0.0115 | 0.0221 | 9.39E-09 | 0.281 |
| **Appendicular lean mass** | **Hand OA** | rs11590254 | T | A | 0.0186 | 0.0159 | 4.34E-20 | 0.2234 |
| **Appendicular lean mass** | **Hand OA** | rs115912456 | G | A | 0.0577 | -0.0175 | 3.69E-34 | 0.5467 |
| **Appendicular lean mass** | **Hand OA** | rs11605297 | A | G | 0.0146 | 0.0045 | 8.03E-11 | 0.7597 |
| **Appendicular lean mass** | **Hand OA** | rs11612462 | G | T | 0.015 | 0.0155 | 2.55E-09 | 0.3467 |
| **Appendicular lean mass** | **Hand OA** | rs11629593 | G | T | -0.0109 | 0.0167 | 4.42E-08 | 0.3371 |
| **Appendicular lean mass** | **Hand OA** | rs11672848 | T | C | -0.0171 | 0.0037 | 7.73E-19 | 0.8252 |
| **Appendicular lean mass** | **Hand OA** | rs117068593 | T | C | 0.0403 | 0.022 | 8.83E-62 | 0.1775 |
| **Appendicular lean mass** | **Hand OA** | rs11721522 | G | A | 0.0106 | 0.012 | 4.03E-08 | 0.3353 |
| **Appendicular lean mass** | **Hand OA** | rs11727162 | T | C | -0.017 | 0.013 | 2.15E-19 | 0.2818 |
| **Appendicular lean mass** | **Hand OA** | rs11867855 | T | G | -0.0132 | -0.002 | 2.93E-09 | 0.889 |
| **Appendicular lean mass** | **Hand OA** | rs12051245 | C | T | 0.0299 | 0.0043 | 2.56E-40 | 0.763 |
| **Appendicular lean mass** | **Hand OA** | rs12150907 | A | G | -0.0219 | -0.0066 | 7.22E-20 | 0.6686 |
| **Appendicular lean mass** | **Hand OA** | rs12188208 | C | A | -0.0195 | -0.0131 | 1.40E-18 | 0.3807 |
| **Appendicular lean mass** | **Hand OA** | rs12347137 | C | A | -0.046 | -0.0182 | 9.80E-85 | 0.2155 |
| **Appendicular lean mass** | **Hand OA** | rs12517711 | C | T | -0.0147 | -0.0107 | 2.79E-14 | 0.4145 |
| **Appendicular lean mass** | **Hand OA** | rs12541381 | A | G | -0.0319 | -0.0121 | 2.81E-49 | 0.4145 |
| **Appendicular lean mass** | **Hand OA** | rs12563442 | C | T | 0.0122 | -0.0127 | 9.93E-09 | 0.3524 |
| **Appendicular lean mass** | **Hand OA** | rs12655296 | T | C | -0.011 | -0.0184 | 1.62E-08 | 0.1435 |
| **Appendicular lean mass** | **Hand OA** | rs12702693 | T | C | 0.0173 | -0.0093 | 6.56E-20 | 0.4449 |
| **Appendicular lean mass** | **Hand OA** | rs12724708 | T | A | 0.0243 | 0.0022 | 1.66E-35 | 0.8859 |
| **Appendicular lean mass** | **Hand OA** | rs1290786 | T | C | -0.0143 | 0.0065 | 7.14E-14 | 0.5942 |
| **Appendicular lean mass** | **Hand OA** | rs12943867 | A | G | 0.0184 | -0.0123 | 7.57E-20 | 0.4225 |
| **Appendicular lean mass** | **Hand OA** | rs13391980 | A | G | -0.0225 | 0.0128 | 7.60E-15 | 0.4733 |
| **Appendicular lean mass** | **Hand OA** | rs1341215 | A | G | 0.0229 | -0.0081 | 6.32E-17 | 0.6398 |
| **Appendicular lean mass** | **Hand OA** | rs143554698 | T | C | -0.0257 | -0.0381 | 3.39E-21 | 0.1216 |
| **Appendicular lean mass** | **Hand OA** | rs1472852 | A | C | -0.0638 | -0.0078 | 8.22E-135 | 0.6696 |
| **Appendicular lean mass** | **Hand OA** | rs14976 | T | C | 0.0144 | 0.0153 | 1.43E-12 | 0.2594 |
| **Appendicular lean mass** | **Hand OA** | rs1514134 | C | T | -0.0114 | 0.0072 | 3.57E-09 | 0.5637 |
| **Appendicular lean mass** | **Hand OA** | rs1556659 | T | C | 0.0163 | 0.001 | 7.19E-17 | 0.9414 |
| **Appendicular lean mass** | **Hand OA** | rs17197114 | C | T | 0.0177 | -0.0103 | 1.54E-12 | 0.5244 |
| **Appendicular lean mass** | **Hand OA** | rs17278379 | C | T | 0.0226 | 0.0158 | 2.40E-15 | 0.3953 |
| **Appendicular lean mass** | **Hand OA** | rs173135 | T | C | -0.0341 | -0.0145 | 3.25E-30 | 0.4504 |
| **Appendicular lean mass** | **Hand OA** | rs17478946 | G | A | -0.0192 | -0.0047 | 9.98E-21 | 0.7324 |
| **Appendicular lean mass** | **Hand OA** | rs1880318 | A | G | 0.0147 | -0.0417 | 6.86E-10 | 0.00721 |
| **Appendicular lean mass** | **Hand OA** | rs2070598 | A | G | 0.0204 | 0.0216 | 6.36E-27 | 0.07545 |
| **Appendicular lean mass** | **Hand OA** | rs2071450 | T | C | -0.0174 | -0.0087 | 8.85E-19 | 0.4857 |
| **Appendicular lean mass** | **Hand OA** | rs2089111 | G | C | -0.0172 | -0.0013 | 1.73E-15 | 0.9258 |
| **Appendicular lean mass** | **Hand OA** | rs2101017 | T | C | -0.0223 | -0.0274 | 1.44E-15 | 0.113 |
| **Appendicular lean mass** | **Hand OA** | rs2142331 | T | C | -0.0165 | -8.00E-04 | 1.38E-17 | 0.9503 |
| **Appendicular lean mass** | **Hand OA** | rs2188805 | C | A | 0.0114 | 0.0312 | 2.00E-08 | 0.01634 |
| **Appendicular lean mass** | **Hand OA** | rs2209098 | C | T | 0.024 | -0.0015 | 1.73E-32 | 0.9069 |
| **Appendicular lean mass** | **Hand OA** | rs2230033 | A | G | -0.0265 | 0.0137 | 3.49E-43 | 0.2637 |
| **Appendicular lean mass** | **Hand OA** | rs2236096 | C | T | 0.018 | 0.0272 | 1.29E-15 | 0.1854 |
| **Appendicular lean mass** | **Hand OA** | rs2237485 | A | G | 0.0191 | -0.0052 | 3.73E-17 | 0.7182 |
| **Appendicular lean mass** | **Hand OA** | rs2268718 | T | C | 0.0141 | 0.0113 | 3.24E-11 | 0.4076 |
| **Appendicular lean mass** | **Hand OA** | rs2289629 | A | G | -0.0148 | -0.0231 | 8.02E-14 | 0.06811 |
| **Appendicular lean mass** | **Hand OA** | rs2347808 | A | G | -0.0125 | 0.0164 | 5.79E-11 | 0.1787 |
| **Appendicular lean mass** | **Hand OA** | rs2569888 | A | G | 0.0133 | -0.0326 | 2.29E-09 | 0.02155 |
| **Appendicular lean mass** | **Hand OA** | rs2578565 | T | C | -0.0141 | -0.0099 | 1.37E-12 | 0.4359 |
| **Appendicular lean mass** | **Hand OA** | rs2592208 | A | C | -0.0124 | -0.0201 | 5.52E-11 | 0.09637 |
| **Appendicular lean mass** | **Hand OA** | rs2648725 | A | T | 0.0165 | 0.0144 | 8.20E-13 | 0.3654 |
| **Appendicular lean mass** | **Hand OA** | rs2663126 | A | G | -0.0139 | 0.0058 | 1.36E-11 | 0.6613 |
| **Appendicular lean mass** | **Hand OA** | rs2754255 | G | A | -0.0153 | -0.0063 | 1.05E-11 | 0.6615 |
| **Appendicular lean mass** | **Hand OA** | rs2763263 | A | T | -0.017 | -0.0062 | 1.37E-14 | 0.6535 |
| **Appendicular lean mass** | **Hand OA** | rs2788213 | A | G | 0.0123 | -0.0059 | 3.80E-09 | 0.6555 |
| **Appendicular lean mass** | **Hand OA** | rs2789365 | T | C | -0.0145 | -0.0015 | 1.14E-14 | 0.899 |
| **Appendicular lean mass** | **Hand OA** | rs28379706 | C | T | 0.0114 | 0.0089 | 4.49E-09 | 0.4756 |
| **Appendicular lean mass** | **Hand OA** | rs28485212 | T | C | -0.0188 | 0.0353 | 1.24E-12 | 0.1228 |
| **Appendicular lean mass** | **Hand OA** | rs28529055 | T | G | -0.0147 | 0.017 | 1.88E-14 | 0.3124 |
| **Appendicular lean mass** | **Hand OA** | rs28529426 | T | C | -0.0168 | 0.0129 | 5.82E-11 | 0.4501 |
| **Appendicular lean mass** | **Hand OA** | rs28678024 | G | A | -0.0119 | -0.0045 | 1.90E-08 | 0.735 |
| **Appendicular lean mass** | **Hand OA** | rs2871865 | G | C | -0.0493 | -0.0063 | 3.40E-62 | 0.751 |
| **Appendicular lean mass** | **Hand OA** | rs2923411 | C | T | 0.0127 | 0.0164 | 4.77E-11 | 0.1872 |
| **Appendicular lean mass** | **Hand OA** | rs2925155 | T | C | -0.015 | -0.0039 | 5.47E-12 | 0.7788 |
| **Appendicular lean mass** | **Hand OA** | rs2978362 | T | C | 0.0106 | -0.0213 | 2.85E-08 | 0.09242 |
| **Appendicular lean mass** | **Hand OA** | rs3116194 | A | T | -0.0295 | 0.0462 | 8.29E-21 | 0.02888 |
| **Appendicular lean mass** | **Hand OA** | rs331917 | G | A | -0.0127 | -6.00E-04 | 3.54E-11 | 0.9621 |
| **Appendicular lean mass** | **Hand OA** | rs336630 | T | C | -0.0106 | -0.0106 | 2.90E-08 | 0.4095 |
| **Appendicular lean mass** | **Hand OA** | rs34312629 | G | C | -0.017 | -0.0163 | 2.12E-15 | 0.2433 |
| **Appendicular lean mass** | **Hand OA** | rs34338597 | G | A | -0.0112 | 0.0213 | 7.88E-09 | 0.08769 |
| **Appendicular lean mass** | **Hand OA** | rs34345560 | A | G | 0.0219 | -0.0164 | 7.10E-20 | 0.292 |
| **Appendicular lean mass** | **Hand OA** | rs34517439 | A | C | 0.0421 | -0.0132 | 5.80E-48 | 0.4936 |
| **Appendicular lean mass** | **Hand OA** | rs35073631 | C | T | 0.0112 | 0.0106 | 5.92E-09 | 0.5294 |
| **Appendicular lean mass** | **Hand OA** | rs35288270 | C | T | -0.0328 | 0.0169 | 3.43E-32 | 0.4726 |
| **Appendicular lean mass** | **Hand OA** | rs35732917 | C | T | 0.0204 | 0.0225 | 2.08E-22 | 0.09061 |
| **Appendicular lean mass** | **Hand OA** | rs35756741 | T | C | -0.0378 | -0.0097 | 5.80E-31 | 0.6307 |
| **Appendicular lean mass** | **Hand OA** | rs35811052 | G | A | -0.0148 | 3.00E-04 | 8.84E-12 | 0.9842 |
| **Appendicular lean mass** | **Hand OA** | rs36000545 | G | A | -0.022 | -0.0149 | 2.56E-29 | 0.3074 |
| **Appendicular lean mass** | **Hand OA** | rs36048468 | T | C | 0.0254 | 0.0122 | 9.34E-28 | 0.4219 |
| **Appendicular lean mass** | **Hand OA** | rs36226649 | C | T | 0.0485 | 0.016 | 3.05E-37 | 0.5001 |
| **Appendicular lean mass** | **Hand OA** | rs3764002 | T | C | 0.028 | -0.0229 | 4.47E-39 | 0.1169 |
| **Appendicular lean mass** | **Hand OA** | rs3782232 | A | G | -0.0339 | -0.0026 | 2.41E-20 | 0.916 |
| **Appendicular lean mass** | **Hand OA** | rs3828729 | G | A | -0.016 | 0.0056 | 4.67E-15 | 0.6699 |
| **Appendicular lean mass** | **Hand OA** | rs40270 | C | A | 0.0151 | -0.0236 | 1.90E-11 | 0.09454 |
| **Appendicular lean mass** | **Hand OA** | rs42039 | T | C | 0.0481 | 0.0073 | 3.53E-106 | 0.6063 |
| **Appendicular lean mass** | **Hand OA** | rs4282339 | A | G | -0.0311 | -0.0178 | 6.16E-41 | 0.2396 |
| **Appendicular lean mass** | **Hand OA** | rs4287835 | C | T | 0.0147 | -0.0011 | 9.94E-15 | 0.9286 |
| **Appendicular lean mass** | **Hand OA** | rs4360494 | C | G | -0.0198 | -0.0092 | 7.88E-26 | 0.4562 |
| **Appendicular lean mass** | **Hand OA** | rs45474992 | T | C | -0.0617 | 0.0081 | 2.17E-33 | 0.8118 |
| **Appendicular lean mass** | **Hand OA** | rs45528934 | T | C | 0.0262 | -0.0352 | 1.97E-24 | 0.04193 |
| **Appendicular lean mass** | **Hand OA** | rs4640244 | G | A | -0.02 | -0.0013 | 3.81E-25 | 0.9174 |
| **Appendicular lean mass** | **Hand OA** | rs4682483 | A | G | -0.0165 | 0.0032 | 2.65E-10 | 0.8472 |
| **Appendicular lean mass** | **Hand OA** | rs4735761 | C | A | 0.0331 | 0.0092 | 3.66E-56 | 0.4936 |
| **Appendicular lean mass** | **Hand OA** | rs4752689 | A | G | 0.0205 | -0.0109 | 1.36E-26 | 0.3963 |
| **Appendicular lean mass** | **Hand OA** | rs4847378 | T | G | 0.0136 | -0.0224 | 1.64E-12 | 0.07096 |
| **Appendicular lean mass** | **Hand OA** | rs4870941 | C | G | -0.0297 | -0.0081 | 1.09E-39 | 0.5809 |
| **Appendicular lean mass** | **Hand OA** | rs4932439 | G | A | -0.0151 | -0.0112 | 1.43E-09 | 0.477 |
| **Appendicular lean mass** | **Hand OA** | rs496783 | G | A | -0.0124 | 0.0097 | 8.13E-11 | 0.4491 |
| **Appendicular lean mass** | **Hand OA** | rs4976262 | C | T | -0.0245 | 0.0021 | 4.37E-33 | 0.8678 |
| **Appendicular lean mass** | **Hand OA** | rs55758152 | A | G | 0.0145 | 0.0058 | 1.05E-12 | 0.7125 |
| **Appendicular lean mass** | **Hand OA** | rs55852614 | C | T | -0.0393 | -0.0183 | 3.29E-73 | 0.1922 |
| **Appendicular lean mass** | **Hand OA** | rs56112295 | T | C | 0.0154 | 0.0465 | 1.12E-10 | 0.01354 |
| **Appendicular lean mass** | **Hand OA** | rs568267 | T | C | 0.0122 | 0.0267 | 2.23E-08 | 0.0624 |
| **Appendicular lean mass** | **Hand OA** | rs57059662 | C | T | 0.0118 | -0.0173 | 5.63E-09 | 0.1815 |
| **Appendicular lean mass** | **Hand OA** | rs57513571 | T | C | -0.0191 | -0.035 | 7.02E-16 | 0.02156 |
| **Appendicular lean mass** | **Hand OA** | rs591668 | A | G | -0.0174 | -0.0044 | 2.00E-19 | 0.7263 |
| **Appendicular lean mass** | **Hand OA** | rs599004 | T | C | -0.0157 | -0.0075 | 6.53E-14 | 0.5836 |
| **Appendicular lean mass** | **Hand OA** | rs59950280 | A | G | -0.0254 | 7.00E-04 | 7.32E-36 | 0.9575 |
| **Appendicular lean mass** | **Hand OA** | rs59985551 | T | C | -0.0313 | 0.0041 | 2.43E-44 | 0.7789 |
| **Appendicular lean mass** | **Hand OA** | rs604723 | C | T | -0.0166 | -0.009 | 8.16E-15 | 0.51 |
| **Appendicular lean mass** | **Hand OA** | rs6142059 | C | T | 0.0116 | 0.0027 | 1.19E-09 | 0.8224 |
| **Appendicular lean mass** | **Hand OA** | rs61919240 | A | T | 0.0137 | 0.0074 | 9.77E-12 | 0.5703 |
| **Appendicular lean mass** | **Hand OA** | rs62033029 | A | G | -0.0141 | 0.0013 | 1.73E-09 | 0.9338 |
| **Appendicular lean mass** | **Hand OA** | rs62106258 | C | T | -0.0504 | -0.0177 | 6.45E-31 | 0.5643 |
| **Appendicular lean mass** | **Hand OA** | rs62143873 | A | G | -0.0115 | 0.0267 | 1.19E-09 | 0.0365 |
| **Appendicular lean mass** | **Hand OA** | rs62466110 | C | T | -0.0371 | -0.0204 | 5.74E-20 | 0.4969 |
| **Appendicular lean mass** | **Hand OA** | rs62501195 | C | A | -0.0198 | -0.0215 | 8.11E-15 | 0.1857 |
| **Appendicular lean mass** | **Hand OA** | rs62515437 | T | G | 0.0369 | -0.0022 | 8.79E-60 | 0.8757 |
| **Appendicular lean mass** | **Hand OA** | rs6582398 | T | C | 0.014 | -0.0232 | 1.14E-12 | 0.1251 |
| **Appendicular lean mass** | **Hand OA** | rs6675858 | T | C | -0.0137 | -0.0168 | 2.40E-09 | 0.2452 |
| **Appendicular lean mass** | **Hand OA** | rs6738207 | A | G | 0.0127 | 0.024 | 4.14E-11 | 0.05225 |
| **Appendicular lean mass** | **Hand OA** | rs67527161 | C | T | -0.0182 | -0.0033 | 5.79E-15 | 0.8287 |
| **Appendicular lean mass** | **Hand OA** | rs67551338 | T | C | 0.0576 | 0.0075 | 1.04E-47 | 0.7548 |
| **Appendicular lean mass** | **Hand OA** | rs67716382 | C | G | 0.0226 | -0.0147 | 1.65E-23 | 0.3171 |
| **Appendicular lean mass** | **Hand OA** | rs6789000 | T | G | 0.0121 | 0.0322 | 1.61E-09 | 0.09409 |
| **Appendicular lean mass** | **Hand OA** | rs68049170 | A | G | -0.0259 | 0.0035 | 2.69E-34 | 0.7937 |
| **Appendicular lean mass** | **Hand OA** | rs6821305 | C | A | 0.0204 | 0.0093 | 3.15E-26 | 0.4794 |
| **Appendicular lean mass** | **Hand OA** | rs684905 | T | C | -0.0118 | -0.0165 | 7.10E-10 | 0.323 |
| **Appendicular lean mass** | **Hand OA** | rs6849302 | G | A | 0.0155 | -0.0123 | 7.11E-11 | 0.4247 |
| **Appendicular lean mass** | **Hand OA** | rs6874142 | G | T | 0.0288 | 0.0286 | 5.15E-20 | 0.2431 |
| **Appendicular lean mass** | **Hand OA** | rs6977416 | A | G | 0.0457 | -0.0049 | 1.43E-113 | 0.7069 |
| **Appendicular lean mass** | **Hand OA** | rs700677 | A | C | 0.0173 | -0.0079 | 1.13E-18 | 0.5486 |
| **Appendicular lean mass** | **Hand OA** | rs7014590 | C | T | -0.0228 | 0.0229 | 4.48E-26 | 0.1047 |
| **Appendicular lean mass** | **Hand OA** | rs702886 | G | A | 0.012 | 0.0168 | 1.11E-09 | 0.1881 |
| **Appendicular lean mass** | **Hand OA** | rs7129320 | A | G | -0.0389 | 0.0111 | 7.29E-53 | 0.4878 |
| **Appendicular lean mass** | **Hand OA** | rs71414738 | T | C | 0.015 | 7.00E-04 | 1.00E-09 | 0.9653 |
| **Appendicular lean mass** | **Hand OA** | rs718603 | T | C | 0.0131 | 0.0174 | 6.68E-10 | 0.1972 |
| **Appendicular lean mass** | **Hand OA** | rs7229520 | A | G | -0.0224 | -0.0041 | 9.21E-29 | 0.7475 |
| **Appendicular lean mass** | **Hand OA** | rs72656010 | C | T | -0.0668 | -0.0187 | 7.31E-126 | 0.3013 |
| **Appendicular lean mass** | **Hand OA** | rs72721979 | G | T | -0.0229 | -0.0133 | 2.20E-17 | 0.4557 |
| **Appendicular lean mass** | **Hand OA** | rs72801843 | A | T | 0.0313 | 0.0103 | 8.83E-52 | 0.4328 |
| **Appendicular lean mass** | **Hand OA** | rs72841270 | G | T | 0.0294 | -0.0054 | 2.25E-26 | 0.766 |
| **Appendicular lean mass** | **Hand OA** | rs7301341 | C | T | -0.0255 | 0.0138 | 9.27E-37 | 0.3054 |
| **Appendicular lean mass** | **Hand OA** | rs73052033 | C | T | -0.0151 | -0.0025 | 4.79E-10 | 0.8701 |
| **Appendicular lean mass** | **Hand OA** | rs7320878 | A | G | -0.015 | 0.047 | 1.34E-14 | 0.004433 |
| **Appendicular lean mass** | **Hand OA** | rs7328187 | G | T | 0.0116 | 5.00E-04 | 1.19E-09 | 0.97 |
| **Appendicular lean mass** | **Hand OA** | rs73384223 | C | T | -0.0205 | -0.0299 | 1.27E-17 | 0.05433 |
| **Appendicular lean mass** | **Hand OA** | rs73413540 | T | C | -0.0124 | 0.0068 | 4.42E-08 | 0.6541 |
| **Appendicular lean mass** | **Hand OA** | rs73696333 | G | C | 0.0191 | -0.0129 | 3.20E-15 | 0.4566 |
| **Appendicular lean mass** | **Hand OA** | rs7418410 | T | C | 0.0155 | -0.0052 | 5.64E-16 | 0.6702 |
| **Appendicular lean mass** | **Hand OA** | rs75022676 | A | G | -0.0163 | 0.0011 | 2.84E-12 | 0.9466 |
| **Appendicular lean mass** | **Hand OA** | rs75702986 | A | G | -0.0163 | 0.0088 | 3.14E-11 | 0.5755 |
| **Appendicular lean mass** | **Hand OA** | rs7598430 | T | C | -0.016 | 0.0278 | 1.37E-17 | 0.02275 |
| **Appendicular lean mass** | **Hand OA** | rs7610055 | A | G | -0.0373 | -0.0323 | 3.55E-38 | 0.07814 |
| **Appendicular lean mass** | **Hand OA** | rs76364830 | A | G | -0.0471 | -8.00E-04 | 2.70E-33 | 0.9771 |
| **Appendicular lean mass** | **Hand OA** | rs7689420 | C | T | 0.0466 | -0.0037 | 1.50E-76 | 0.8281 |
| **Appendicular lean mass** | **Hand OA** | rs7701233 | C | T | -0.0179 | 1.00E-04 | 5.12E-21 | 0.9923 |
| **Appendicular lean mass** | **Hand OA** | rs772222 | G | A | 0.0121 | -0.0037 | 1.55E-08 | 0.7852 |
| **Appendicular lean mass** | **Hand OA** | rs7731023 | G | A | 0.0166 | 0.0093 | 3.48E-18 | 0.4715 |
| **Appendicular lean mass** | **Hand OA** | rs7768973 | A | T | -0.024 | -0.0189 | 5.44E-36 | 0.1264 |
| **Appendicular lean mass** | **Hand OA** | rs7828086 | C | T | 0.0135 | 0.0215 | 1.11E-09 | 0.1326 |
| **Appendicular lean mass** | **Hand OA** | rs78766798 | C | T | 0.0319 | 0.0389 | 2.61E-20 | 0.1137 |
| **Appendicular lean mass** | **Hand OA** | rs7952436 | T | C | -0.0453 | -0.0478 | 1.62E-39 | 0.07326 |
| **Appendicular lean mass** | **Hand OA** | rs7971536 | A | T | -0.0194 | 0.0202 | 1.06E-24 | 0.09779 |
| **Appendicular lean mass** | **Hand OA** | rs8017006 | G | A | 0.0122 | -0.0342 | 2.22E-09 | 0.06406 |
| **Appendicular lean mass** | **Hand OA** | rs8018486 | G | A | -0.0138 | 0.0137 | 1.18E-08 | 0.363 |
| **Appendicular lean mass** | **Hand OA** | rs80295797 | T | C | -0.0198 | 0.006 | 3.83E-23 | 0.647 |
| **Appendicular lean mass** | **Hand OA** | rs8084413 | A | G | -0.0127 | 2.00E-04 | 3.25E-11 | 0.9869 |
| **Appendicular lean mass** | **Hand OA** | rs861674 | T | A | 0.0128 | 0.02 | 1.36E-11 | 0.09975 |
| **Appendicular lean mass** | **Hand OA** | rs9266244 | A | G | -0.0427 | 0.003 | 1.21E-94 | 0.8762 |
| **Appendicular lean mass** | **Hand OA** | rs9343327 | T | A | 0.014 | 0.0296 | 1.09E-13 | 0.01561 |
| **Appendicular lean mass** | **Hand OA** | rs9375188 | T | C | 0.0136 | 0.013 | 6.80E-13 | 0.2853 |
| **Appendicular lean mass** | **Hand OA** | rs9385002 | T | A | -0.0147 | 0.0093 | 3.26E-11 | 0.5122 |
| **Appendicular lean mass** | **Hand OA** | rs951366 | C | T | 0.0205 | -0.0239 | 9.15E-27 | 0.05213 |
| **Appendicular lean mass** | **Hand OA** | rs9568031 | T | C | -0.0115 | 0.0118 | 3.34E-08 | 0.3705 |
| **Appendicular lean mass** | **Hand OA** | rs9636364 | A | G | 0.011 | 0.0057 | 5.09E-09 | 0.6383 |
| **Appendicular lean mass** | **Hand OA** | rs9809116 | G | A | -0.016 | -0.0107 | 1.31E-16 | 0.3882 |
| **Hand grip strength (left)** | **Hand OA** | rs10097417 | G | A | -0.0132302 | 0.0285 | 1.90E-11 | 0.07959 |
| **Hand grip strength (left)** | **Hand OA** | rs10176878 | C | T | -0.0129478 | 0.026 | 8.60E-12 | 0.09848 |
| **Hand grip strength (left)** | **Hand OA** | rs10403906 | A | G | -0.0100323 | 0.012 | 1.50E-11 | 0.3268 |
| **Hand grip strength (left)** | **Hand OA** | rs10786706 | T | C | 0.0100072 | 0.0144 | 1.70E-11 | 0.2553 |
| **Hand grip strength (left)** | **Hand OA** | rs10934857 | A | G | 0.00928675 | 0.0171 | 4.80E-08 | 0.2218 |
| **Hand grip strength (left)** | **Hand OA** | rs10988217 | G | A | -0.00920657 | 0.0151 | 1.70E-09 | 0.2219 |
| **Hand grip strength (left)** | **Hand OA** | rs11002322 | T | G | -0.00998924 | 0.0194 | 2.10E-10 | 0.1306 |
| **Hand grip strength (left)** | **Hand OA** | rs11121542 | A | G | -0.0157601 | 0.0244 | 3.00E-12 | 0.1854 |
| **Hand grip strength (left)** | **Hand OA** | rs11243202 | C | T | 0.00972237 | -0.0108 | 7.00E-11 | 0.3738 |
| **Hand grip strength (left)** | **Hand OA** | rs11642954 | A | G | -0.0117025 | 0.0095 | 4.50E-10 | 0.5319 |
| **Hand grip strength (left)** | **Hand OA** | rs12473732 | T | C | 0.0109869 | 0.0024 | 1.40E-13 | 0.8467 |
| **Hand grip strength (left)** | **Hand OA** | rs12673062 | A | G | -0.0107691 | 0.0246 | 2.60E-09 | 0.1038 |
| **Hand grip strength (left)** | **Hand OA** | rs12790261 | A | C | -0.0251882 | -0.0471 | 1.20E-20 | 0.07715 |
| **Hand grip strength (left)** | **Hand OA** | rs12889267 | G | A | -0.0137394 | 0.0037 | 4.70E-12 | 0.8303 |
| **Hand grip strength (left)** | **Hand OA** | rs12906830 | C | T | 0.0108401 | -0.002 | 9.30E-13 | 0.8735 |
| **Hand grip strength (left)** | **Hand OA** | rs13091492 | G | A | -0.00847903 | -0.0121 | 3.30E-08 | 0.3299 |
| **Hand grip strength (left)** | **Hand OA** | rs13146142 | C | T | -0.0202031 | -0.0095 | 2.30E-23 | 0.6007 |
| **Hand grip strength (left)** | **Hand OA** | rs13337177 | T | G | -0.0142799 | -0.0143 | 1.60E-13 | 0.4229 |
| **Hand grip strength (left)** | **Hand OA** | rs1556659 | T | C | 0.0162896 | 0.001 | 2.50E-26 | 0.9414 |
| **Hand grip strength (left)** | **Hand OA** | rs16870531 | T | C | 0.011198 | -0.0091 | 1.30E-10 | 0.5259 |
| **Hand grip strength (left)** | **Hand OA** | rs17282763 | C | T | 0.00893956 | -0.0051 | 4.40E-08 | 0.6955 |
| **Hand grip strength (left)** | **Hand OA** | rs17466480 | G | A | -0.0118235 | 0.0047 | 9.80E-15 | 0.7034 |
| **Hand grip strength (left)** | **Hand OA** | rs181766 | C | T | 0.00963247 | -0.0017 | 1.90E-09 | 0.894 |
| **Hand grip strength (left)** | **Hand OA** | rs1884447 | A | G | 0.00846069 | -0.0031 | 2.30E-08 | 0.8048 |
| **Hand grip strength (left)** | **Hand OA** | rs2359239 | T | C | -0.00881461 | 0.0256 | 6.90E-09 | 0.03967 |
| **Hand grip strength (left)** | **Hand OA** | rs2871865 | G | C | -0.0217979 | -0.0063 | 5.00E-21 | 0.751 |
| **Hand grip strength (left)** | **Hand OA** | rs2871960 | C | A | 0.0120879 | -0.0186 | 5.50E-16 | 0.127 |
| **Hand grip strength (left)** | **Hand OA** | rs2974438 | A | G | -0.0100735 | -0.0183 | 3.30E-08 | 0.2255 |
| **Hand grip strength (left)** | **Hand OA** | rs35175534 | C | A | -0.0163733 | -0.0021 | 3.60E-12 | 0.9647 |
| **Hand grip strength (left)** | **Hand OA** | rs3814877 | T | G | 0.0105385 | 0.0376 | 3.40E-12 | 0.002248 |
| **Hand grip strength (left)** | **Hand OA** | rs4121165 | A | G | -0.0114169 | -0.0045 | 3.40E-10 | 0.7682 |
| **Hand grip strength (left)** | **Hand OA** | rs4575361 | T | A | -0.0108028 | -0.0113 | 1.60E-11 | 0.3846 |
| **Hand grip strength (left)** | **Hand OA** | rs4621706 | T | C | -0.0117068 | -0.016 | 6.00E-15 | 0.1943 |
| **Hand grip strength (left)** | **Hand OA** | rs4713506 | A | G | -0.0157268 | -0.0265 | 2.00E-20 | 0.07952 |
| **Hand grip strength (left)** | **Hand OA** | rs4962700 | G | C | 0.00904307 | -0.0269 | 3.20E-08 | 0.1021 |
| **Hand grip strength (left)** | **Hand OA** | rs56338231 | G | A | -0.010847 | -0.0022 | 1.70E-10 | 0.8722 |
| **Hand grip strength (left)** | **Hand OA** | rs59116179 | T | C | 0.00857385 | 0.0169 | 2.40E-08 | 0.1991 |
| **Hand grip strength (left)** | **Hand OA** | rs61286123 | C | T | -0.0101041 | 0.0143 | 1.20E-08 | 0.3211 |
| **Hand grip strength (left)** | **Hand OA** | rs62081464 | T | C | -0.0098827 | 0.0261 | 2.80E-08 | 0.06685 |
| **Hand grip strength (left)** | **Hand OA** | rs6689375 | T | A | -0.0160169 | 0.0212 | 5.30E-17 | 0.1753 |
| **Hand grip strength (left)** | **Hand OA** | rs7197751 | T | G | -0.00946321 | -0.0025 | 1.40E-09 | 0.8482 |
| **Hand grip strength (left)** | **Hand OA** | rs73307079 | C | T | 0.0111025 | -0.004 | 1.40E-09 | 0.7875 |
| **Hand grip strength (left)** | **Hand OA** | rs755547 | A | G | 0.0165156 | -8.00E-04 | 3.20E-18 | 0.9603 |
| **Hand grip strength (left)** | **Hand OA** | rs772014 | G | A | -0.0106178 | -0.0243 | 2.70E-12 | 0.05859 |
| **Hand grip strength (left)** | **Hand OA** | rs7856625 | T | C | -0.0111297 | 0.0335 | 2.50E-13 | 0.007036 |
| **Hand grip strength (left)** | **Hand OA** | rs7963801 | C | T | -0.0104348 | -0.0236 | 4.60E-12 | 0.06906 |
| **Hand grip strength (left)** | **Hand OA** | rs821100 | G | A | -0.0101977 | 0.0326 | 1.50E-09 | 0.01936 |
| **Hand grip strength (left)** | **Hand OA** | rs9371201 | T | C | -0.00933847 | 0.0149 | 3.00E-09 | 0.2451 |
| **Hand grip strength (left)** | **Hand OA** | rs9371881 | A | G | 0.00947946 | -0.0167 | 9.60E-10 | 0.2087 |
| **Hand grip strength (left)** | **Hand OA** | rs9388769 | A | G | -0.0140821 | -0.0112 | 5.10E-19 | 0.3878 |
| **Hand grip strength (left)** | **Hand OA** | rs9611273 | T | C | 0.0107888 | -0.0284 | 4.50E-10 | 0.0475 |
| **Hand grip strength (left)** | **Hand OA** | rs9944324 | G | A | -0.00855272 | -0.0037 | 1.20E-08 | 0.762 |
| **Hand grip strength (right)** | **Hand OA** | rs10278546 | C | A | 0.0107539 | -0.0266 | 1.10E-08 | 0.08208 |
| **Hand grip strength (right)** | **Hand OA** | rs1043515 | G | A | 0.0138281 | -0.0151 | 2.80E-20 | 0.2124 |
| **Hand grip strength (right)** | **Hand OA** | rs1047891 | A | C | 0.0096867 | 0.0213 | 1.30E-09 | 0.1692 |
| **Hand grip strength (right)** | **Hand OA** | rs10770125 | G | A | 0.00844132 | 0.0055 | 1.40E-08 | 0.6509 |
| **Hand grip strength (right)** | **Hand OA** | rs10784502 | T | C | -0.0111247 | -0.0102 | 7.10E-14 | 0.4027 |
| **Hand grip strength (right)** | **Hand OA** | rs10799428 | T | C | -0.0143588 | 0.0247 | 4.60E-14 | 0.1143 |
| **Hand grip strength (right)** | **Hand OA** | rs11039348 | A | G | -0.00976855 | -0.0183 | 3.90E-10 | 0.1616 |
| **Hand grip strength (right)** | **Hand OA** | rs11243202 | C | T | 0.0116378 | -0.0108 | 6.50E-15 | 0.3738 |
| **Hand grip strength (right)** | **Hand OA** | rs1125 | A | G | -0.0100304 | 0.0167 | 1.90E-10 | 0.1909 |
| **Hand grip strength (right)** | **Hand OA** | rs113315602 | C | A | -0.0212911 | -0.117 | 1.40E-15 | 0.05299 |
| **Hand grip strength (right)** | **Hand OA** | rs113835839 | T | C | -0.00986353 | 0.0305 | 1.10E-08 | 0.02949 |
| **Hand grip strength (right)** | **Hand OA** | rs11642954 | A | G | -0.0131918 | 0.0095 | 2.20E-12 | 0.5319 |
| **Hand grip strength (right)** | **Hand OA** | rs12412806 | A | G | -0.00909582 | 0.0042 | 3.00E-08 | 0.7526 |
| **Hand grip strength (right)** | **Hand OA** | rs12452505 | G | C | -0.0144313 | 0.0166 | 1.40E-11 | 0.3774 |
| **Hand grip strength (right)** | **Hand OA** | rs12790261 | A | C | -0.0263714 | -0.0471 | 2.00E-22 | 0.07715 |
| **Hand grip strength (right)** | **Hand OA** | rs12823922 | G | A | -0.0114203 | 0.0057 | 1.60E-10 | 0.699 |
| **Hand grip strength (right)** | **Hand OA** | rs12889267 | G | A | -0.0122874 | 0.0037 | 6.70E-10 | 0.8303 |
| **Hand grip strength (right)** | **Hand OA** | rs13146142 | C | T | -0.0207968 | -0.0095 | 1.40E-24 | 0.6007 |
| **Hand grip strength (right)** | **Hand OA** | rs13169333 | C | T | 0.00929083 | -0.019 | 4.30E-08 | 0.1641 |
| **Hand grip strength (right)** | **Hand OA** | rs1442883 | A | C | -0.0106189 | 0.0266 | 5.80E-10 | 0.0604 |
| **Hand grip strength (right)** | **Hand OA** | rs1550115 | T | C | 0.0152836 | -0.0091 | 4.50E-19 | 0.5201 |
| **Hand grip strength (right)** | **Hand OA** | rs1556659 | T | C | 0.0175345 | 0.001 | 3.80E-30 | 0.9414 |
| **Hand grip strength (right)** | **Hand OA** | rs1885690 | A | C | -0.00839964 | 0.0162 | 2.80E-08 | 0.2115 |
| **Hand grip strength (right)** | **Hand OA** | rs1952256 | G | A | 0.00976791 | -0.014 | 4.10E-10 | 0.2673 |
| **Hand grip strength (right)** | **Hand OA** | rs2194747 | G | A | 0.00985126 | 0.0014 | 1.90E-09 | 0.9179 |
| **Hand grip strength (right)** | **Hand OA** | rs2226685 | C | T | 0.0103333 | -0.0048 | 3.10E-09 | 0.7466 |
| **Hand grip strength (right)** | **Hand OA** | rs246181 | T | C | 0.00970432 | 0.0084 | 3.70E-10 | 0.5007 |
| **Hand grip strength (right)** | **Hand OA** | rs248831 | A | G | 0.00988989 | 0.01 | 8.40E-09 | 0.4786 |
| **Hand grip strength (right)** | **Hand OA** | rs2854152 | G | A | 0.010991 | 0.0184 | 6.00E-12 | 0.1584 |
| **Hand grip strength (right)** | **Hand OA** | rs2871865 | G | C | -0.0236863 | -0.0063 | 1.80E-24 | 0.751 |
| **Hand grip strength (right)** | **Hand OA** | rs35175534 | C | A | -0.0191822 | -0.0021 | 4.20E-16 | 0.9647 |
| **Hand grip strength (right)** | **Hand OA** | rs35833641 | G | A | 0.00922306 | 0.0087 | 8.50E-09 | 0.5084 |
| **Hand grip strength (right)** | **Hand OA** | rs3848369 | T | C | -0.00953776 | -0.0016 | 4.70E-10 | 0.8969 |
| **Hand grip strength (right)** | **Hand OA** | rs4121165 | A | G | -0.0119792 | -0.0045 | 4.80E-11 | 0.7682 |
| **Hand grip strength (right)** | **Hand OA** | rs4549685 | T | C | 0.00971494 | -0.0015 | 8.10E-10 | 0.9058 |
| **Hand grip strength (right)** | **Hand OA** | rs4751671 | A | G | 0.0082332 | -0.0104 | 4.10E-08 | 0.4175 |
| **Hand grip strength (right)** | **Hand OA** | rs4752689 | A | G | 0.0087531 | -0.0109 | 6.40E-09 | 0.3963 |
| **Hand grip strength (right)** | **Hand OA** | rs4868110 | T | A | -0.00971069 | -0.0182 | 1.00E-09 | 0.1587 |
| **Hand grip strength (right)** | **Hand OA** | rs4962700 | G | C | 0.00932733 | -0.0269 | 1.20E-08 | 0.1021 |
| **Hand grip strength (right)** | **Hand OA** | rs56074046 | A | G | -0.00903502 | -0.0124 | 4.40E-09 | 0.3228 |
| **Hand grip strength (right)** | **Hand OA** | rs56365901 | G | A | -0.0142618 | 0.013 | 1.80E-15 | 0.3974 |
| **Hand grip strength (right)** | **Hand OA** | rs600038 | C | T | -0.0103761 | 0.02 | 1.50E-08 | 0.1875 |
| **Hand grip strength (right)** | **Hand OA** | rs62037412 | A | G | 0.00932966 | -0.0099 | 1.90E-09 | 0.4407 |
| **Hand grip strength (right)** | **Hand OA** | rs645144 | C | T | -0.00868793 | -0.0055 | 4.30E-08 | 0.6753 |
| **Hand grip strength (right)** | **Hand OA** | rs6473015 | C | A | 0.00959961 | 0.0096 | 5.50E-09 | 0.4728 |
| **Hand grip strength (right)** | **Hand OA** | rs6693567 | T | C | -0.00974656 | -0.0144 | 6.30E-09 | 0.2869 |
| **Hand grip strength (right)** | **Hand OA** | rs6693965 | T | G | -0.016251 | 0.0208 | 3.20E-13 | 0.2497 |
| **Hand grip strength (right)** | **Hand OA** | rs6792762 | A | G | -0.00911683 | 0.0145 | 1.80E-09 | 0.2394 |
| **Hand grip strength (right)** | **Hand OA** | rs721101 | C | T | 0.00946697 | -0.017 | 1.60E-08 | 0.2216 |
| **Hand grip strength (right)** | **Hand OA** | rs7266065 | A | G | 0.00993881 | -0.0017 | 4.60E-10 | 0.8971 |
| **Hand grip strength (right)** | **Hand OA** | rs7301953 | A | G | -0.0115397 | -0.0126 | 6.40E-13 | 0.3348 |
| **Hand grip strength (right)** | **Hand OA** | rs7790322 | T | C | -0.00860301 | 0.0039 | 1.20E-08 | 0.7492 |
| **Hand grip strength (right)** | **Hand OA** | rs7871404 | G | A | 0.0119345 | -0.0093 | 3.40E-10 | 0.5429 |
| **Hand grip strength (right)** | **Hand OA** | rs7963801 | C | T | -0.0112734 | -0.0236 | 8.40E-14 | 0.06906 |
| **Hand grip strength (right)** | **Hand OA** | rs8055199 | A | G | -0.00896262 | -0.0023 | 1.20E-08 | 0.8617 |
| **Hand grip strength (right)** | **Hand OA** | rs935728 | T | C | 0.00955337 | -0.0039 | 1.80E-09 | 0.7622 |
| **Hand grip strength (right)** | **Hand OA** | rs9639938 | G | C | 0.00873031 | 8.00E-04 | 5.20E-09 | 0.9447 |
| **Hand grip strength (right)** | **Hand OA** | rs9652468 | A | G | -0.0125308 | 0.0231 | 3.30E-13 | 0.09313 |
| **Hand grip strength (right)** | **Hand OA** | rs9757079 | T | C | 0.00980223 | -0.0033 | 8.40E-10 | 0.8013 |
| **Hand grip strength (right)** | **Hand OA** | rs9853018 | T | C | 0.0101977 | -0.0172 | 8.80E-12 | 0.1564 |
| **Usual walking pace** | **Hand OA** | rs10828258 | G | A | -0.00932641 | -0.0058 |  | 0.6541 |
| **Usual walking pace** | **Hand OA** | rs10862220 | G | T | 0.00840767 | -0.0119 | 4.80E-10 | 0.3539 |
| **Usual walking pace** | **Hand OA** | rs11039324 | A | G | -0.0102105 | -0.0127 | 2.20E-15 | 0.3157 |
| **Usual walking pace** | **Hand OA** | rs11150623 | T | G | -0.00968813 | 0.0097 | 2.40E-13 | 0.4498 |
| **Usual walking pace** | **Hand OA** | rs113825410 | G | A | -0.00881182 | 0.0162 | 6.40E-09 | 0.26 |
| **Usual walking pace** | **Hand OA** | rs11682482 | G | T | 0.00801894 | 0.0412 | 3.30E-09 | 0.001559 |
| **Usual walking pace** | **Hand OA** | rs11732213 | C | T | 0.0091614 | -0.0057 | 9.40E-09 | 0.7088 |
| **Usual walking pace** | **Hand OA** | rs11761141 | G | T | -0.00789936 | -0.0202 | 5.00E-09 | 0.1145 |
| **Usual walking pace** | **Hand OA** | rs11848096 | C | T | -0.00750831 | -0.0278 | 9.20E-09 | 0.02568 |
| **Usual walking pace** | **Hand OA** | rs12461902 | A | G | -0.00801134 | 0.0012 | 3.60E-09 | 0.9287 |
| **Usual walking pace** | **Hand OA** | rs205262 | G | A | -0.00870949 | 0.0118 | 1.00E-09 | 0.3912 |
| **Usual walking pace** | **Hand OA** | rs2170670 | A | G | -0.00708769 | 5.00E-04 | 4.70E-08 | 0.9703 |
| **Usual walking pace** | **Hand OA** | rs2280406 | A | G | -0.00996145 | 0.0124 | 3.40E-15 | 0.3062 |
| **Usual walking pace** | **Hand OA** | rs2439823 | G | A | -0.0072789 | -0.0063 | 1.10E-08 | 0.6224 |
| **Usual walking pace** | **Hand OA** | rs2602731 | G | A | -0.00766675 | -0.0014 | 2.10E-08 | 0.914 |
| **Usual walking pace** | **Hand OA** | rs273512 | T | C | -0.00966507 | 0.0025 | 6.90E-14 | 0.8376 |
| **Usual walking pace** | **Hand OA** | rs35711462 | G | A | -0.0071659 | 0.0198 | 1.70E-08 | 0.1023 |
| **Usual walking pace** | **Hand OA** | rs4109292 | A | G | 0.00735336 | -0.0188 | 6.60E-09 | 0.1365 |
| **Appendicular lean mass** | **Hip OA** | rs10112506 | G | A | -0.012 | -0.0193 | 5.76E-10 | 0.03885 |
| **Appendicular lean mass** | **Hip OA** | rs10203320 | C | T | 0.0138 | 0.0235 | 7.86E-12 | 0.01634 |
| **Appendicular lean mass** | **Hip OA** | rs1047891 | A | C | 0.0233 | 0.0121 | 5.70E-31 | 0.2457 |
| **Appendicular lean mass** | **Hip OA** | rs1063582 | G | T | -0.0185 | -1.00E-04 | 1.12E-16 | 0.9895 |
| **Appendicular lean mass** | **Hip OA** | rs10776560 | T | C | -0.0157 | -0.0153 | 7.88E-17 | 0.08929 |
| **Appendicular lean mass** | **Hip OA** | rs10822117 | G | A | -0.0176 | 0.0103 | 4.24E-15 | 0.3334 |
| **Appendicular lean mass** | **Hip OA** | rs10845408 | T | C | 0.0255 | 0.0104 | 3.25E-38 | 0.2697 |
| **Appendicular lean mass** | **Hip OA** | rs10858246 | C | G | -0.0188 | -0.0016 | 2.35E-20 | 0.8677 |
| **Appendicular lean mass** | **Hip OA** | rs10864899 | G | A | -0.0112 | -0.0107 | 3.85E-09 | 0.3189 |
| **Appendicular lean mass** | **Hip OA** | rs11014285 | A | G | 0.0342 | 0.0034 | 2.89E-40 | 0.7877 |
| **Appendicular lean mass** | **Hip OA** | rs11121615 | T | C | -0.0202 | 0.0262 | 3.32E-23 | 0.01245 |
| **Appendicular lean mass** | **Hip OA** | rs11178643 | T | A | 0.0109 | -0.0028 | 4.44E-08 | 0.8419 |
| **Appendicular lean mass** | **Hip OA** | rs11191208 | A | G | 0.0147 | 0.0062 | 3.69E-10 | 0.5907 |
| **Appendicular lean mass** | **Hip OA** | rs11198591 | A | G | 0.0148 | 4.00E-04 | 4.91E-14 | 0.9682 |
| **Appendicular lean mass** | **Hip OA** | rs11210892 | A | G | 0.0118 | 8.00E-04 | 3.57E-09 | 0.9355 |
| **Appendicular lean mass** | **Hip OA** | rs11217863 | A | G | -0.0268 | 0.0184 | 1.06E-19 | 0.1845 |
| **Appendicular lean mass** | **Hip OA** | rs11243202 | C | T | 0.0302 | 0.0089 | 2.83E-57 | 0.3261 |
| **Appendicular lean mass** | **Hip OA** | rs112537273 | C | T | -0.0212 | 0.014 | 3.34E-21 | 0.1935 |
| **Appendicular lean mass** | **Hip OA** | rs11260035 | A | G | 0.015 | 0.0142 | 1.86E-12 | 0.1609 |
| **Appendicular lean mass** | **Hip OA** | rs113671109 | C | T | -0.015 | -0.0076 | 4.23E-11 | 0.485 |
| **Appendicular lean mass** | **Hip OA** | rs115010283 | C | G | 0.034 | -0.0141 | 2.35E-63 | 0.3101 |
| **Appendicular lean mass** | **Hip OA** | rs11562101 | T | A | 0.0115 | -0.0128 | 9.39E-09 | 0.3569 |
| **Appendicular lean mass** | **Hip OA** | rs11580040 | G | A | 0.0325 | -0.0171 | 6.76E-21 | 0.3201 |
| **Appendicular lean mass** | **Hip OA** | rs11590254 | T | A | 0.0186 | 0.0103 | 4.34E-20 | 0.2872 |
| **Appendicular lean mass** | **Hip OA** | rs115912456 | G | A | 0.0577 | -0.0391 | 3.69E-34 | 0.08106 |
| **Appendicular lean mass** | **Hip OA** | rs11605297 | A | G | 0.0146 | 9.00E-04 | 8.03E-11 | 0.932 |
| **Appendicular lean mass** | **Hip OA** | rs11612462 | G | T | 0.015 | -0.0156 | 2.55E-09 | 0.2002 |
| **Appendicular lean mass** | **Hip OA** | rs11629593 | G | T | -0.0109 | -0.0073 | 4.42E-08 | 0.5378 |
| **Appendicular lean mass** | **Hip OA** | rs11672848 | T | C | -0.0171 | 0.0086 | 7.73E-19 | 0.423 |
| **Appendicular lean mass** | **Hip OA** | rs117068593 | T | C | 0.0403 | 0.003 | 8.83E-62 | 0.8113 |
| **Appendicular lean mass** | **Hip OA** | rs11721522 | G | A | 0.0106 | 0.0121 | 4.03E-08 | 0.1878 |
| **Appendicular lean mass** | **Hip OA** | rs11727162 | T | C | -0.017 | -0.006 | 2.15E-19 | 0.5054 |
| **Appendicular lean mass** | **Hip OA** | rs11867855 | T | G | -0.0132 | 0.0085 | 2.93E-09 | 0.4189 |
| **Appendicular lean mass** | **Hip OA** | rs12051245 | C | T | 0.0299 | 0.0104 | 2.56E-40 | 0.3339 |
| **Appendicular lean mass** | **Hip OA** | rs12150907 | A | G | -0.0219 | 0.0122 | 7.22E-20 | 0.2909 |
| **Appendicular lean mass** | **Hip OA** | rs12517711 | C | T | -0.0147 | -0.0243 | 2.79E-14 | 0.01004 |
| **Appendicular lean mass** | **Hip OA** | rs12541381 | A | G | -0.0319 | 0.0036 | 2.81E-49 | 0.7301 |
| **Appendicular lean mass** | **Hip OA** | rs12563442 | C | T | 0.0122 | 0.0083 | 9.93E-09 | 0.4103 |
| **Appendicular lean mass** | **Hip OA** | rs12655296 | T | C | -0.011 | -1.00E-04 | 1.62E-08 | 0.9943 |
| **Appendicular lean mass** | **Hip OA** | rs12702693 | T | C | 0.0173 | 0.0176 | 6.56E-20 | 0.0523 |
| **Appendicular lean mass** | **Hip OA** | rs12724708 | T | A | 0.0243 | -0.0317 | 1.66E-35 | 0.001942 |
| **Appendicular lean mass** | **Hip OA** | rs1290786 | T | C | -0.0143 | -0.0061 | 7.14E-14 | 0.5052 |
| **Appendicular lean mass** | **Hip OA** | rs12943867 | A | G | 0.0184 | -0.0265 | 7.57E-20 | 0.01193 |
| **Appendicular lean mass** | **Hip OA** | rs13391980 | A | G | -0.0225 | -0.0204 | 7.60E-15 | 0.1383 |
| **Appendicular lean mass** | **Hip OA** | rs143554698 | T | C | -0.0257 | -0.0122 | 3.39E-21 | 0.4343 |
| **Appendicular lean mass** | **Hip OA** | rs1472852 | A | C | -0.0638 | 0.0066 | 8.22E-135 | 0.6098 |
| **Appendicular lean mass** | **Hip OA** | rs1478575 | A | T | 0.0312 | -0.0139 | 5.20E-54 | 0.1456 |
| **Appendicular lean mass** | **Hip OA** | rs14976 | T | C | 0.0144 | 0.0074 | 1.43E-12 | 0.4567 |
| **Appendicular lean mass** | **Hip OA** | rs1514134 | C | T | -0.0114 | -0.0208 | 3.57E-09 | 0.02356 |
| **Appendicular lean mass** | **Hip OA** | rs1556659 | T | C | 0.0163 | -0.0042 | 7.19E-17 | 0.6633 |
| **Appendicular lean mass** | **Hip OA** | rs17197114 | C | T | 0.0177 | 0.0064 | 1.54E-12 | 0.6151 |
| **Appendicular lean mass** | **Hip OA** | rs17278379 | C | T | 0.0226 | 0.0337 | 2.40E-15 | 0.01518 |
| **Appendicular lean mass** | **Hip OA** | rs173135 | T | C | -0.0341 | 0.0042 | 3.25E-30 | 0.7728 |
| **Appendicular lean mass** | **Hip OA** | rs17478946 | G | A | -0.0192 | -0.0216 | 9.98E-21 | 0.03103 |
| **Appendicular lean mass** | **Hip OA** | rs1880318 | A | G | 0.0147 | -0.0097 | 6.86E-10 | 0.4015 |
| **Appendicular lean mass** | **Hip OA** | rs2070598 | A | G | 0.0204 | 0.0122 | 6.36E-27 | 0.1763 |
| **Appendicular lean mass** | **Hip OA** | rs2071450 | T | C | -0.0174 | -0.0012 | 8.85E-19 | 0.9019 |
| **Appendicular lean mass** | **Hip OA** | rs2089111 | G | C | -0.0172 | -0.0029 | 1.73E-15 | 0.7815 |
| **Appendicular lean mass** | **Hip OA** | rs2101017 | T | C | -0.0223 | 0.0041 | 1.44E-15 | 0.7515 |
| **Appendicular lean mass** | **Hip OA** | rs2142331 | T | C | -0.0165 | -0.0099 | 1.38E-17 | 0.2886 |
| **Appendicular lean mass** | **Hip OA** | rs2188805 | C | A | 0.0114 | 0.0158 | 2.00E-08 | 0.1087 |
| **Appendicular lean mass** | **Hip OA** | rs2209098 | C | T | 0.024 | -0.0071 | 1.73E-32 | 0.4611 |
| **Appendicular lean mass** | **Hip OA** | rs2230033 | A | G | -0.0265 | 6.00E-04 | 3.49E-43 | 0.9429 |
| **Appendicular lean mass** | **Hip OA** | rs2236096 | C | T | 0.018 | 0.0027 | 1.29E-15 | 0.8444 |
| **Appendicular lean mass** | **Hip OA** | rs2237485 | A | G | 0.0191 | -0.0206 | 3.73E-17 | 0.05303 |
| **Appendicular lean mass** | **Hip OA** | rs2268718 | T | C | 0.0141 | -0.0115 | 3.24E-11 | 0.2549 |
| **Appendicular lean mass** | **Hip OA** | rs2289629 | A | G | -0.0148 | 0.004 | 8.02E-14 | 0.6721 |
| **Appendicular lean mass** | **Hip OA** | rs2347808 | A | G | -0.0125 | -0.0129 | 5.79E-11 | 0.1597 |
| **Appendicular lean mass** | **Hip OA** | rs244711 | T | C | 0.0279 | 0.0162 | 1.54E-37 | 0.1399 |
| **Appendicular lean mass** | **Hip OA** | rs2569888 | A | G | 0.0133 | 0.0073 | 2.29E-09 | 0.4922 |
| **Appendicular lean mass** | **Hip OA** | rs2578565 | T | C | -0.0141 | -0.0054 | 1.37E-12 | 0.5663 |
| **Appendicular lean mass** | **Hip OA** | rs2592208 | A | C | -0.0124 | -0.0026 | 5.52E-11 | 0.7748 |
| **Appendicular lean mass** | **Hip OA** | rs2648725 | A | T | 0.0165 | 0.0013 | 8.20E-13 | 0.9102 |
| **Appendicular lean mass** | **Hip OA** | rs2663126 | A | G | -0.0139 | -0.0069 | 1.36E-11 | 0.4872 |
| **Appendicular lean mass** | **Hip OA** | rs2754255 | G | A | -0.0153 | 0.0149 | 1.05E-11 | 0.1663 |
| **Appendicular lean mass** | **Hip OA** | rs2763263 | A | T | -0.017 | 0.0111 | 1.37E-14 | 0.2826 |
| **Appendicular lean mass** | **Hip OA** | rs2788213 | A | G | 0.0123 | 0.0086 | 3.80E-09 | 0.3832 |
| **Appendicular lean mass** | **Hip OA** | rs2789365 | T | C | -0.0145 | 0.0098 | 1.14E-14 | 0.2747 |
| **Appendicular lean mass** | **Hip OA** | rs28379706 | C | T | 0.0114 | -7.00E-04 | 4.49E-09 | 0.9419 |
| **Appendicular lean mass** | **Hip OA** | rs28485212 | T | C | -0.0188 | 0.0083 | 1.24E-12 | 0.5672 |
| **Appendicular lean mass** | **Hip OA** | rs28529055 | T | G | -0.0147 | 0.0113 | 1.88E-14 | 0.3251 |
| **Appendicular lean mass** | **Hip OA** | rs28529426 | T | C | -0.0168 | 0.0085 | 5.82E-11 | 0.4917 |
| **Appendicular lean mass** | **Hip OA** | rs28592876 | A | G | 0.03 | -0.0187 | 9.09E-38 | 0.09263 |
| **Appendicular lean mass** | **Hip OA** | rs28678024 | G | A | -0.0119 | -0.0203 | 1.90E-08 | 0.04417 |
| **Appendicular lean mass** | **Hip OA** | rs2871865 | G | C | -0.0493 | 0.0026 | 3.40E-62 | 0.8635 |
| **Appendicular lean mass** | **Hip OA** | rs2871960 | C | A | 0.0469 | 0.002 | 2.17E-135 | 0.8231 |
| **Appendicular lean mass** | **Hip OA** | rs2923411 | C | T | 0.0127 | -0.0058 | 4.77E-11 | 0.5256 |
| **Appendicular lean mass** | **Hip OA** | rs2925155 | T | C | -0.015 | -0.0132 | 5.47E-12 | 0.2137 |
| **Appendicular lean mass** | **Hip OA** | rs2978362 | T | C | 0.0106 | 7.00E-04 | 2.85E-08 | 0.9437 |
| **Appendicular lean mass** | **Hip OA** | rs3116194 | A | T | -0.0295 | 0.0153 | 8.29E-21 | 0.3239 |
| **Appendicular lean mass** | **Hip OA** | rs331917 | G | A | -0.0127 | 0.013 | 3.54E-11 | 0.1529 |
| **Appendicular lean mass** | **Hip OA** | rs336630 | T | C | -0.0106 | -0.0136 | 2.90E-08 | 0.1492 |
| **Appendicular lean mass** | **Hip OA** | rs34312629 | G | C | -0.017 | -0.0142 | 2.12E-15 | 0.1683 |
| **Appendicular lean mass** | **Hip OA** | rs34338597 | G | A | -0.0112 | -0.0107 | 7.88E-09 | 0.246 |
| **Appendicular lean mass** | **Hip OA** | rs34345560 | A | G | 0.0219 | 0.0053 | 7.10E-20 | 0.6397 |
| **Appendicular lean mass** | **Hip OA** | rs34517439 | A | C | 0.0421 | 1.00E-04 | 5.80E-48 | 0.9969 |
| **Appendicular lean mass** | **Hip OA** | rs35073631 | C | T | 0.0112 | 0.0035 | 5.92E-09 | 0.7616 |
| **Appendicular lean mass** | **Hip OA** | rs35288270 | C | T | -0.0328 | -0.0027 | 3.43E-32 | 0.8585 |
| **Appendicular lean mass** | **Hip OA** | rs35732917 | C | T | 0.0204 | -0.004 | 2.08E-22 | 0.6853 |
| **Appendicular lean mass** | **Hip OA** | rs35756741 | T | C | -0.0378 | 0.0151 | 5.80E-31 | 0.3214 |
| **Appendicular lean mass** | **Hip OA** | rs35811052 | G | A | -0.0148 | 0.0031 | 8.84E-12 | 0.7693 |
| **Appendicular lean mass** | **Hip OA** | rs36000545 | G | A | -0.022 | -0.0078 | 2.56E-29 | 0.431 |
| **Appendicular lean mass** | **Hip OA** | rs36048468 | T | C | 0.0254 | -0.0024 | 9.34E-28 | 0.8313 |
| **Appendicular lean mass** | **Hip OA** | rs36226649 | C | T | 0.0485 | 0.0074 | 3.05E-37 | 0.6864 |
| **Appendicular lean mass** | **Hip OA** | rs3764002 | T | C | 0.028 | -0.0278 | 4.47E-39 | 0.007213 |
| **Appendicular lean mass** | **Hip OA** | rs3782232 | A | G | -0.0339 | -0.0224 | 2.41E-20 | 0.2142 |
| **Appendicular lean mass** | **Hip OA** | rs3828729 | G | A | -0.016 | -0.0261 | 4.67E-15 | 0.007873 |
| **Appendicular lean mass** | **Hip OA** | rs40270 | C | A | 0.0151 | 0.0111 | 1.90E-11 | 0.2923 |
| **Appendicular lean mass** | **Hip OA** | rs4282339 | A | G | -0.0311 | -0.0047 | 6.16E-41 | 0.6738 |
| **Appendicular lean mass** | **Hip OA** | rs4287835 | C | T | 0.0147 | 0.0089 | 9.94E-15 | 0.3246 |
| **Appendicular lean mass** | **Hip OA** | rs4360494 | C | G | -0.0198 | -0.0166 | 7.88E-26 | 0.07399 |
| **Appendicular lean mass** | **Hip OA** | rs45474992 | T | C | -0.0617 | -0.0324 | 2.17E-33 | 0.1886 |
| **Appendicular lean mass** | **Hip OA** | rs45528934 | T | C | 0.0262 | 0.0048 | 1.97E-24 | 0.7059 |
| **Appendicular lean mass** | **Hip OA** | rs4640244 | G | A | -0.02 | 8.00E-04 | 3.81E-25 | 0.9324 |
| **Appendicular lean mass** | **Hip OA** | rs4682483 | A | G | -0.0165 | 0.0085 | 2.65E-10 | 0.4841 |
| **Appendicular lean mass** | **Hip OA** | rs4752689 | A | G | 0.0205 | -0.0186 | 1.36E-26 | 0.04546 |
| **Appendicular lean mass** | **Hip OA** | rs4847378 | T | G | 0.0136 | -0.0256 | 1.64E-12 | 0.005613 |
| **Appendicular lean mass** | **Hip OA** | rs4870941 | C | G | -0.0297 | 0.0025 | 1.09E-39 | 0.8161 |
| **Appendicular lean mass** | **Hip OA** | rs4932439 | G | A | -0.0151 | -0.0015 | 1.43E-09 | 0.8944 |
| **Appendicular lean mass** | **Hip OA** | rs496783 | G | A | -0.0124 | -0.0033 | 8.13E-11 | 0.7212 |
| **Appendicular lean mass** | **Hip OA** | rs4976262 | C | T | -0.0245 | 0.0054 | 4.37E-33 | 0.573 |
| **Appendicular lean mass** | **Hip OA** | rs55758152 | A | G | 0.0145 | 0.0153 | 1.05E-12 | 0.1495 |
| **Appendicular lean mass** | **Hip OA** | rs55852614 | C | T | -0.0393 | 0.0117 | 3.29E-73 | 0.2605 |
| **Appendicular lean mass** | **Hip OA** | rs56112295 | T | C | 0.0154 | 0.0107 | 1.12E-10 | 0.4213 |
| **Appendicular lean mass** | **Hip OA** | rs568267 | T | C | 0.0122 | -0.0022 | 2.23E-08 | 0.8349 |
| **Appendicular lean mass** | **Hip OA** | rs57059662 | C | T | 0.0118 | -0.0193 | 5.63E-09 | 0.04545 |
| **Appendicular lean mass** | **Hip OA** | rs57513571 | T | C | -0.0191 | -0.0247 | 7.02E-16 | 0.02975 |
| **Appendicular lean mass** | **Hip OA** | rs591668 | A | G | -0.0174 | -0.0065 | 2.00E-19 | 0.4835 |
| **Appendicular lean mass** | **Hip OA** | rs599004 | T | C | -0.0157 | -0.0063 | 6.53E-14 | 0.5374 |
| **Appendicular lean mass** | **Hip OA** | rs59950280 | A | G | -0.0254 | -0.0194 | 7.32E-36 | 0.04969 |
| **Appendicular lean mass** | **Hip OA** | rs59985551 | T | C | -0.0313 | -0.0296 | 2.43E-44 | 0.005458 |
| **Appendicular lean mass** | **Hip OA** | rs604723 | C | T | -0.0166 | -0.0308 | 8.16E-15 | 0.002293 |
| **Appendicular lean mass** | **Hip OA** | rs6142059 | C | T | 0.0116 | 0.0098 | 1.19E-09 | 0.278 |
| **Appendicular lean mass** | **Hip OA** | rs61919240 | A | T | 0.0137 | -0.0155 | 9.77E-12 | 0.1062 |
| **Appendicular lean mass** | **Hip OA** | rs62033029 | A | G | -0.0141 | -0.0131 | 1.73E-09 | 0.2517 |
| **Appendicular lean mass** | **Hip OA** | rs62106258 | C | T | -0.0504 | -0.0722 | 6.45E-31 | 0.001654 |
| **Appendicular lean mass** | **Hip OA** | rs62143873 | A | G | -0.0115 | 0.0249 | 1.19E-09 | 0.007153 |
| **Appendicular lean mass** | **Hip OA** | rs62466110 | C | T | -0.0371 | -0.0322 | 5.74E-20 | 0.1136 |
| **Appendicular lean mass** | **Hip OA** | rs62501195 | C | A | -0.0198 | -0.0236 | 8.11E-15 | 0.05052 |
| **Appendicular lean mass** | **Hip OA** | rs62515437 | T | G | 0.0369 | 0.0047 | 8.79E-60 | 0.6657 |
| **Appendicular lean mass** | **Hip OA** | rs6505216 | T | G | -0.0498 | -0.0312 | 1.83E-101 | 0.02225 |
| **Appendicular lean mass** | **Hip OA** | rs6582398 | T | C | 0.014 | 0.0242 | 1.14E-12 | 0.01657 |
| **Appendicular lean mass** | **Hip OA** | rs6675858 | T | C | -0.0137 | -0.0114 | 2.40E-09 | 0.294 |
| **Appendicular lean mass** | **Hip OA** | rs6738207 | A | G | 0.0127 | 0.006 | 4.14E-11 | 0.5089 |
| **Appendicular lean mass** | **Hip OA** | rs67527161 | C | T | -0.0182 | -0.0181 | 5.79E-15 | 0.1072 |
| **Appendicular lean mass** | **Hip OA** | rs67551338 | T | C | 0.0576 | 0.0131 | 1.04E-47 | 0.4786 |
| **Appendicular lean mass** | **Hip OA** | rs67716382 | C | G | 0.0226 | 0.0034 | 1.65E-23 | 0.7544 |
| **Appendicular lean mass** | **Hip OA** | rs6789000 | T | G | 0.0121 | -0.0036 | 1.61E-09 | 0.7933 |
| **Appendicular lean mass** | **Hip OA** | rs68049170 | A | G | -0.0259 | 0.0038 | 2.69E-34 | 0.7022 |
| **Appendicular lean mass** | **Hip OA** | rs6821305 | C | A | 0.0204 | -0.0045 | 3.15E-26 | 0.6481 |
| **Appendicular lean mass** | **Hip OA** | rs684905 | T | C | -0.0118 | -9.00E-04 | 7.10E-10 | 0.9332 |
| **Appendicular lean mass** | **Hip OA** | rs6849302 | G | A | 0.0155 | -0.0053 | 7.11E-11 | 0.6395 |
| **Appendicular lean mass** | **Hip OA** | rs6874142 | G | T | 0.0288 | 0.02 | 5.15E-20 | 0.2334 |
| **Appendicular lean mass** | **Hip OA** | rs700677 | A | C | 0.0173 | -0.017 | 1.13E-18 | 0.0801 |
| **Appendicular lean mass** | **Hip OA** | rs7014590 | C | T | -0.0228 | 0.0158 | 4.48E-26 | 0.1265 |
| **Appendicular lean mass** | **Hip OA** | rs702886 | G | A | 0.012 | 0.0216 | 1.11E-09 | 0.02253 |
| **Appendicular lean mass** | **Hip OA** | rs7129320 | A | G | -0.0389 | -0.0206 | 7.29E-53 | 0.09691 |
| **Appendicular lean mass** | **Hip OA** | rs71414738 | T | C | 0.015 | -0.0073 | 1.00E-09 | 0.543 |
| **Appendicular lean mass** | **Hip OA** | rs718603 | T | C | 0.0131 | 0.0088 | 6.68E-10 | 0.3758 |
| **Appendicular lean mass** | **Hip OA** | rs7229520 | A | G | -0.0224 | 0.0019 | 9.21E-29 | 0.8412 |
| **Appendicular lean mass** | **Hip OA** | rs72656010 | C | T | -0.0668 | -0.0031 | 7.31E-126 | 0.816 |
| **Appendicular lean mass** | **Hip OA** | rs72721979 | G | T | -0.0229 | -0.011 | 2.20E-17 | 0.409 |
| **Appendicular lean mass** | **Hip OA** | rs72894003 | T | C | -0.0423 | 0.067 | 1.90E-28 | 0.00101 |
| **Appendicular lean mass** | **Hip OA** | rs73052033 | C | T | -0.0151 | -0.0215 | 4.79E-10 | 0.06571 |
| **Appendicular lean mass** | **Hip OA** | rs7320878 | A | G | -0.015 | -0.017 | 1.34E-14 | 0.1122 |
| **Appendicular lean mass** | **Hip OA** | rs7328187 | G | T | 0.0116 | 0.0138 | 1.19E-09 | 0.1256 |
| **Appendicular lean mass** | **Hip OA** | rs73384223 | C | T | -0.0205 | 0.0027 | 1.27E-17 | 0.816 |
| **Appendicular lean mass** | **Hip OA** | rs73413540 | T | C | -0.0124 | -0.0127 | 4.42E-08 | 0.2581 |
| **Appendicular lean mass** | **Hip OA** | rs73696333 | G | C | 0.0191 | -0.0195 | 3.20E-15 | 0.1084 |
| **Appendicular lean mass** | **Hip OA** | rs7418410 | T | C | 0.0155 | -0.0066 | 5.64E-16 | 0.4719 |
| **Appendicular lean mass** | **Hip OA** | rs75022676 | A | G | -0.0163 | 0.0082 | 2.84E-12 | 0.4685 |
| **Appendicular lean mass** | **Hip OA** | rs75702986 | A | G | -0.0163 | 0.0101 | 3.14E-11 | 0.3886 |
| **Appendicular lean mass** | **Hip OA** | rs7598430 | T | C | -0.016 | -0.0077 | 1.37E-17 | 0.3919 |
| **Appendicular lean mass** | **Hip OA** | rs7610055 | A | G | -0.0373 | 0.0095 | 3.55E-38 | 0.4795 |
| **Appendicular lean mass** | **Hip OA** | rs76364830 | A | G | -0.0471 | -0.0215 | 2.70E-33 | 0.2857 |
| **Appendicular lean mass** | **Hip OA** | rs7689420 | C | T | 0.0466 | 0.0194 | 1.50E-76 | 0.1148 |
| **Appendicular lean mass** | **Hip OA** | rs7701233 | C | T | -0.0179 | -0.0081 | 5.12E-21 | 0.4088 |
| **Appendicular lean mass** | **Hip OA** | rs772222 | G | A | 0.0121 | 0.0041 | 1.55E-08 | 0.6912 |
| **Appendicular lean mass** | **Hip OA** | rs7731023 | G | A | 0.0166 | -0.0054 | 3.48E-18 | 0.5609 |
| **Appendicular lean mass** | **Hip OA** | rs78766798 | C | T | 0.0319 | -0.0013 | 2.61E-20 | 0.9428 |
| **Appendicular lean mass** | **Hip OA** | rs7971536 | A | T | -0.0194 | -0.0074 | 1.06E-24 | 0.4096 |
| **Appendicular lean mass** | **Hip OA** | rs8017006 | G | A | 0.0122 | 0.0092 | 2.22E-09 | 0.4723 |
| **Appendicular lean mass** | **Hip OA** | rs8018486 | G | A | -0.0138 | -0.0025 | 1.18E-08 | 0.8243 |
| **Appendicular lean mass** | **Hip OA** | rs8019890 | A | C | 0.025 | 0.009 | 1.96E-38 | 0.3352 |
| **Appendicular lean mass** | **Hip OA** | rs80295797 | T | C | -0.0198 | 0.0168 | 3.83E-23 | 0.08397 |
| **Appendicular lean mass** | **Hip OA** | rs8084413 | A | G | -0.0127 | 0.0117 | 3.25E-11 | 0.2104 |
| **Appendicular lean mass** | **Hip OA** | rs861674 | T | A | 0.0128 | 0.0198 | 1.36E-11 | 0.0285 |
| **Appendicular lean mass** | **Hip OA** | rs900399 | G | A | 0.0164 | 9.00E-04 | 1.35E-17 | 0.9244 |
| **Appendicular lean mass** | **Hip OA** | rs905938 | C | T | 0.0394 | 0 | 8.43E-77 | 0.999 |
| **Appendicular lean mass** | **Hip OA** | rs9266244 | A | G | -0.0427 | -0.0298 | 1.21E-94 | 0.0191 |
| **Appendicular lean mass** | **Hip OA** | rs9343327 | T | A | 0.014 | 0.0293 | 1.09E-13 | 0.001183 |
| **Appendicular lean mass** | **Hip OA** | rs9375188 | T | C | 0.0136 | 0.0087 | 6.80E-13 | 0.3316 |
| **Appendicular lean mass** | **Hip OA** | rs9385002 | T | A | -0.0147 | 0.01 | 3.26E-11 | 0.3433 |
| **Appendicular lean mass** | **Hip OA** | rs9391254 | T | C | 0.0166 | 0.0255 | 2.10E-16 | 0.01123 |
| **Appendicular lean mass** | **Hip OA** | rs951366 | C | T | 0.0205 | -0.0094 | 9.15E-27 | 0.3049 |
| **Appendicular lean mass** | **Hip OA** | rs9568031 | T | C | -0.0115 | -0.0132 | 3.34E-08 | 0.1818 |
| **Appendicular lean mass** | **Hip OA** | rs9634212 | A | C | 0.0471 | -0.0029 | 8.59E-95 | 0.7904 |
| **Appendicular lean mass** | **Hip OA** | rs9636364 | A | G | 0.011 | 0.0113 | 5.09E-09 | 0.2124 |
| **Appendicular lean mass** | **Hip OA** | rs9809116 | G | A | -0.016 | -0.0071 | 1.31E-16 | 0.4404 |
| **Appendicular lean mass** | **Hip OA** | rs9894577 | A | G | -0.031 | 0.0191 | 1.40E-52 | 0.04851 |
| **Hand grip strength (left)** | **Hip OA** | rs10097417 | G | A | -0.0132302 | -0.0098 | 1.90E-11 | 0.4117 |
| **Hand grip strength (left)** | **Hip OA** | rs10176878 | C | T | -0.0129478 | 0.0127 | 8.60E-12 | 0.2831 |
| **Hand grip strength (left)** | **Hip OA** | rs10403906 | A | G | -0.0100323 | -0.0015 | 1.50E-11 | 0.8708 |
| **Hand grip strength (left)** | **Hip OA** | rs10786706 | T | C | 0.0100072 | -0.0066 | 1.70E-11 | 0.4701 |
| **Hand grip strength (left)** | **Hip OA** | rs10934857 | A | G | 0.00928675 | 0.0122 | 4.80E-08 | 0.2404 |
| **Hand grip strength (left)** | **Hip OA** | rs10988217 | G | A | -0.00920657 | -0.0086 | 1.70E-09 | 0.3573 |
| **Hand grip strength (left)** | **Hip OA** | rs11002322 | T | G | -0.00998924 | 0.0056 | 2.10E-10 | 0.5583 |
| **Hand grip strength (left)** | **Hip OA** | rs11121542 | A | G | -0.0157601 | -0.0096 | 3.00E-12 | 0.4832 |
| **Hand grip strength (left)** | **Hip OA** | rs11243202 | C | T | 0.00972237 | 0.0089 | 7.00E-11 | 0.3261 |
| **Hand grip strength (left)** | **Hip OA** | rs11642954 | A | G | -0.0117025 | 0.001 | 4.50E-10 | 0.9303 |
| **Hand grip strength (left)** | **Hip OA** | rs12473732 | T | C | 0.0109869 | -0.0068 | 1.40E-13 | 0.4596 |
| **Hand grip strength (left)** | **Hip OA** | rs12673062 | A | G | -0.0107691 | 0.0086 | 2.60E-09 | 0.4368 |
| **Hand grip strength (left)** | **Hip OA** | rs12889267 | G | A | -0.0137394 | -0.0141 | 4.70E-12 | 0.258 |
| **Hand grip strength (left)** | **Hip OA** | rs12906830 | C | T | 0.0108401 | -0.0096 | 9.30E-13 | 0.2953 |
| **Hand grip strength (left)** | **Hip OA** | rs13091492 | G | A | -0.00847903 | -0.0025 | 3.30E-08 | 0.7866 |
| **Hand grip strength (left)** | **Hip OA** | rs13146142 | C | T | -0.0202031 | 0.0075 | 2.30E-23 | 0.5631 |
| **Hand grip strength (left)** | **Hip OA** | rs13337177 | T | G | -0.0142799 | -0.0038 | 1.60E-13 | 0.7672 |
| **Hand grip strength (left)** | **Hip OA** | rs1556659 | T | C | 0.0162896 | -0.0042 | 2.50E-26 | 0.6633 |
| **Hand grip strength (left)** | **Hip OA** | rs16870531 | T | C | 0.011198 | 0.003 | 1.30E-10 | 0.7817 |
| **Hand grip strength (left)** | **Hip OA** | rs17282763 | C | T | 0.00893956 | -0.0042 | 4.40E-08 | 0.6692 |
| **Hand grip strength (left)** | **Hip OA** | rs1884447 | A | G | 0.00846069 | -0.0114 | 2.30E-08 | 0.2208 |
| **Hand grip strength (left)** | **Hip OA** | rs2359239 | T | C | -0.00881461 | 0.0062 | 6.90E-09 | 0.5025 |
| **Hand grip strength (left)** | **Hip OA** | rs2871865 | G | C | -0.0217979 | 0.0026 | 5.00E-21 | 0.8635 |
| **Hand grip strength (left)** | **Hip OA** | rs2871960 | C | A | 0.0120879 | 0.002 | 5.50E-16 | 0.8231 |
| **Hand grip strength (left)** | **Hip OA** | rs2974438 | A | G | -0.0100735 | -0.0038 | 3.30E-08 | 0.7342 |
| **Hand grip strength (left)** | **Hip OA** | rs35175534 | C | A | -0.0163733 | -0.0197 | 3.60E-12 | 0.389 |
| **Hand grip strength (left)** | **Hip OA** | rs4121165 | A | G | -0.0114169 | 0.0073 | 3.40E-10 | 0.5139 |
| **Hand grip strength (left)** | **Hip OA** | rs4575361 | T | A | -0.0108028 | -0.0091 | 1.60E-11 | 0.3506 |
| **Hand grip strength (left)** | **Hip OA** | rs4713506 | A | G | -0.0157268 | -0.0158 | 2.00E-20 | 0.1696 |
| **Hand grip strength (left)** | **Hip OA** | rs4962700 | G | C | 0.00904307 | 0.0096 | 3.20E-08 | 0.3705 |
| **Hand grip strength (left)** | **Hip OA** | rs59116179 | T | C | 0.00857385 | 0.0061 | 2.40E-08 | 0.5229 |
| **Hand grip strength (left)** | **Hip OA** | rs61286123 | C | T | -0.0101041 | -0.0049 | 1.20E-08 | 0.6467 |
| **Hand grip strength (left)** | **Hip OA** | rs62081464 | T | C | -0.0098827 | -0.0197 | 2.80E-08 | 0.0661 |
| **Hand grip strength (left)** | **Hip OA** | rs6689375 | T | A | -0.0160169 | 0.0011 | 5.30E-17 | 0.9267 |
| **Hand grip strength (left)** | **Hip OA** | rs7197751 | T | G | -0.00946321 | -0.0137 | 1.40E-09 | 0.1509 |
| **Hand grip strength (left)** | **Hip OA** | rs73307079 | C | T | 0.0111025 | -0.0104 | 1.40E-09 | 0.3345 |
| **Hand grip strength (left)** | **Hip OA** | rs755547 | A | G | 0.0165156 | 0.0036 | 3.20E-18 | 0.7594 |
| **Hand grip strength (left)** | **Hip OA** | rs772014 | G | A | -0.0106178 | -0.0068 | 2.70E-12 | 0.4702 |
| **Hand grip strength (left)** | **Hip OA** | rs7963801 | C | T | -0.0104348 | -0.017 | 4.60E-12 | 0.07075 |
| **Hand grip strength (left)** | **Hip OA** | rs821100 | G | A | -0.0101977 | 0.0154 | 1.50E-09 | 0.1324 |
| **Hand grip strength (left)** | **Hip OA** | rs9371201 | T | C | -0.00933847 | 0.0038 | 3.00E-09 | 0.6888 |
| **Hand grip strength (left)** | **Hip OA** | rs9371881 | A | G | 0.00947946 | 0.0038 | 9.60E-10 | 0.6987 |
| **Hand grip strength (left)** | **Hip OA** | rs9611273 | T | C | 0.0107888 | 0.003 | 4.50E-10 | 0.7749 |
| **Hand grip strength (left)** | **Hip OA** | rs9944324 | G | A | -0.00855272 | -0.0109 | 1.20E-08 | 0.2328 |
| **Hand grip strength (right)** | **Hip OA** | rs10278546 | C | A | 0.0107539 | -0.0076 | 1.10E-08 | 0.5031 |
| **Hand grip strength (right)** | **Hip OA** | rs1043515 | G | A | 0.0138281 | 0.0031 | 2.80E-20 | 0.7319 |
| **Hand grip strength (right)** | **Hip OA** | rs1047891 | A | C | 0.0096867 | 0.0121 | 1.30E-09 | 0.2457 |
| **Hand grip strength (right)** | **Hip OA** | rs10784502 | T | C | -0.0111247 | -0.0031 | 7.10E-14 | 0.7269 |
| **Hand grip strength (right)** | **Hip OA** | rs10798483 | A | G | 0.0145083 | 0.0113 | 2.80E-22 | 0.2178 |
| **Hand grip strength (right)** | **Hip OA** | rs10799428 | T | C | -0.0143588 | -4.00E-04 | 4.60E-14 | 0.9745 |
| **Hand grip strength (right)** | **Hip OA** | rs11243202 | C | T | 0.0116378 | 0.0089 | 6.50E-15 | 0.3261 |
| **Hand grip strength (right)** | **Hip OA** | rs1125 | A | G | -0.0100304 | 0.0018 | 1.90E-10 | 0.8471 |
| **Hand grip strength (right)** | **Hip OA** | rs113315602 | C | A | -0.0212911 | -0.0056 | 1.40E-15 | 0.8236 |
| **Hand grip strength (right)** | **Hip OA** | rs113835839 | T | C | -0.00986353 | 0.0024 | 1.10E-08 | 0.8199 |
| **Hand grip strength (right)** | **Hip OA** | rs11642954 | A | G | -0.0131918 | 0.001 | 2.20E-12 | 0.9303 |
| **Hand grip strength (right)** | **Hip OA** | rs12412806 | A | G | -0.00909582 | -0.0057 | 3.00E-08 | 0.5624 |
| **Hand grip strength (right)** | **Hip OA** | rs12452505 | G | C | -0.0144313 | -0.0026 | 1.40E-11 | 0.8522 |
| **Hand grip strength (right)** | **Hip OA** | rs12823922 | G | A | -0.0114203 | 0.0085 | 1.60E-10 | 0.4316 |
| **Hand grip strength (right)** | **Hip OA** | rs12889267 | G | A | -0.0122874 | -0.0141 | 6.70E-10 | 0.258 |
| **Hand grip strength (right)** | **Hip OA** | rs13146142 | C | T | -0.0207968 | 0.0075 | 1.40E-24 | 0.5631 |
| **Hand grip strength (right)** | **Hip OA** | rs13169333 | C | T | 0.00929083 | -0.0101 | 4.30E-08 | 0.3202 |
| **Hand grip strength (right)** | **Hip OA** | rs1442883 | A | C | -0.0106189 | 0.01 | 5.80E-10 | 0.3436 |
| **Hand grip strength (right)** | **Hip OA** | rs1550115 | T | C | 0.0152836 | -0.0107 | 4.50E-19 | 0.3036 |
| **Hand grip strength (right)** | **Hip OA** | rs1556659 | T | C | 0.0175345 | -0.0042 | 3.80E-30 | 0.6633 |
| **Hand grip strength (right)** | **Hip OA** | rs2194747 | G | A | 0.00985126 | 0.0032 | 1.90E-09 | 0.7486 |
| **Hand grip strength (right)** | **Hip OA** | rs248831 | A | G | 0.00988989 | -0.0126 | 8.40E-09 | 0.2307 |
| **Hand grip strength (right)** | **Hip OA** | rs2854152 | G | A | 0.010991 | 0.009 | 6.00E-12 | 0.355 |
| **Hand grip strength (right)** | **Hip OA** | rs2871865 | G | C | -0.0236863 | 0.0026 | 1.80E-24 | 0.8635 |
| **Hand grip strength (right)** | **Hip OA** | rs35175534 | C | A | -0.0191822 | -0.0197 | 4.20E-16 | 0.389 |
| **Hand grip strength (right)** | **Hip OA** | rs35833641 | G | A | 0.00922306 | -0.0102 | 8.50E-09 | 0.2932 |
| **Hand grip strength (right)** | **Hip OA** | rs3848369 | T | C | -0.00953776 | -0.0031 | 4.70E-10 | 0.7397 |
| **Hand grip strength (right)** | **Hip OA** | rs4121165 | A | G | -0.0119792 | 0.0073 | 4.80E-11 | 0.5139 |
| **Hand grip strength (right)** | **Hip OA** | rs4549685 | T | C | 0.00971494 | 0.0144 | 8.10E-10 | 0.1309 |
| **Hand grip strength (right)** | **Hip OA** | rs4751671 | A | G | 0.0082332 | 0.0028 | 4.10E-08 | 0.7619 |
| **Hand grip strength (right)** | **Hip OA** | rs4752689 | A | G | 0.0087531 | -0.0186 | 6.40E-09 | 0.04546 |
| **Hand grip strength (right)** | **Hip OA** | rs4868110 | T | A | -0.00971069 | -0.0154 | 1.00E-09 | 0.1075 |
| **Hand grip strength (right)** | **Hip OA** | rs4962700 | G | C | 0.00932733 | 0.0096 | 1.20E-08 | 0.3705 |
| **Hand grip strength (right)** | **Hip OA** | rs600038 | C | T | -0.0103761 | -0.0077 | 1.50E-08 | 0.4861 |
| **Hand grip strength (right)** | **Hip OA** | rs62037412 | A | G | 0.00932966 | 9.00E-04 | 1.90E-09 | 0.9255 |
| **Hand grip strength (right)** | **Hip OA** | rs645144 | C | T | -0.00868793 | -0.0091 | 4.30E-08 | 0.351 |
| **Hand grip strength (right)** | **Hip OA** | rs6473015 | C | A | 0.00959961 | 0.0042 | 5.50E-09 | 0.674 |
| **Hand grip strength (right)** | **Hip OA** | rs6693965 | T | G | -0.016251 | -0.0122 | 3.20E-13 | 0.3665 |
| **Hand grip strength (right)** | **Hip OA** | rs6792762 | A | G | -0.00911683 | 4.00E-04 | 1.80E-09 | 0.9652 |
| **Hand grip strength (right)** | **Hip OA** | rs6870324 | G | C | -0.00998568 | -0.0031 | 3.00E-09 | 0.7638 |
| **Hand grip strength (right)** | **Hip OA** | rs721101 | C | T | 0.00946697 | 0.0025 | 1.60E-08 | 0.8122 |
| **Hand grip strength (right)** | **Hip OA** | rs7266065 | A | G | 0.00993881 | -9.00E-04 | 4.60E-10 | 0.9221 |
| **Hand grip strength (right)** | **Hip OA** | rs7301953 | A | G | -0.0115397 | -0.0088 | 6.40E-13 | 0.3636 |
| **Hand grip strength (right)** | **Hip OA** | rs7790322 | T | C | -0.00860301 | 0.0109 | 1.20E-08 | 0.2291 |
| **Hand grip strength (right)** | **Hip OA** | rs7871404 | G | A | 0.0119345 | 0.0144 | 3.40E-10 | 0.2082 |
| **Hand grip strength (right)** | **Hip OA** | rs7963801 | C | T | -0.0112734 | -0.017 | 8.40E-14 | 0.07075 |
| **Hand grip strength (right)** | **Hip OA** | rs9322822 | T | C | 0.0110799 | 0.0161 | 3.50E-12 | 0.09321 |
| **Hand grip strength (right)** | **Hip OA** | rs935728 | T | C | 0.00955337 | 0.0095 | 1.80E-09 | 0.3197 |
| **Hand grip strength (right)** | **Hip OA** | rs9639938 | G | C | 0.00873031 | 0.0014 | 5.20E-09 | 0.8751 |
| **Hand grip strength (right)** | **Hip OA** | rs9757079 | T | C | 0.00980223 | -0.0104 | 8.40E-10 | 0.2866 |
| **Hand grip strength (right)** | **Hip OA** | rs9853018 | T | C | 0.0101977 | 0.0021 | 8.80E-12 | 0.8187 |
| **Usual walking pace** | **Hip OA** | rs10828258 | G | A | -0.00932641 | 0.0155 | 6.70E-12 | 0.1178 |
| **Usual walking pace** | **Hip OA** | rs10862220 | G | T | 0.00840767 | -0.0069 | 4.80E-10 | 0.4695 |
| **Usual walking pace** | **Hip OA** | rs11150623 | T | G | -0.00968813 | -0.0024 | 2.40E-13 | 0.7997 |
| **Usual walking pace** | **Hip OA** | rs113825410 | G | A | -0.00881182 | 0.0265 | 6.40E-09 | 0.01366 |
| **Usual walking pace** | **Hip OA** | rs11682482 | G | T | 0.00801894 | -0.001 | 3.30E-09 | 0.9139 |
| **Usual walking pace** | **Hip OA** | rs11761141 | G | T | -0.00789936 | -0.0094 | 5.00E-09 | 0.3217 |
| **Usual walking pace** | **Hip OA** | rs11848096 | C | T | -0.00750831 | 0.0088 | 9.20E-09 | 0.3452 |
| **Usual walking pace** | **Hip OA** | rs12461902 | A | G | -0.00801134 | 0.0018 | 3.60E-09 | 0.8515 |
| **Usual walking pace** | **Hip OA** | rs205262 | G | A | -0.00870949 | 0.0164 | 1.00E-09 | 0.1083 |
| **Usual walking pace** | **Hip OA** | rs2170670 | A | G | -0.00708769 | 3.00E-04 | 4.70E-08 | 0.9721 |
| **Usual walking pace** | **Hip OA** | rs2280406 | A | G | -0.00996145 | 0.0105 | 3.40E-15 | 0.2426 |
| **Usual walking pace** | **Hip OA** | rs2439823 | G | A | -0.0072789 | -0.0078 | 1.10E-08 | 0.399 |
| **Usual walking pace** | **Hip OA** | rs2602731 | G | A | -0.00766675 | 0.0151 | 2.10E-08 | 0.122 |
| **Usual walking pace** | **Hip OA** | rs273512 | T | C | -0.00966507 | 0.0047 | 6.90E-14 | 0.6103 |
| **Usual walking pace** | **Hip OA** | rs35711462 | G | A | -0.0071659 | 0.0142 | 1.70E-08 | 0.1126 |
| **Usual walking pace** | **Hip OA** | rs4109292 | A | G | 0.00735336 | -0.002 | 6.60E-09 | 0.8252 |
| **Appendicular lean mass** | **Knee OA** | rs10112506 | G | A | -0.012 | -0.0161 | 5.76E-10 | 0.02768 |
| **Appendicular lean mass** | **Knee OA** | rs10203320 | C | T | 0.0138 | 0.0266 | 7.86E-12 | 5.38E-04 |
| **Appendicular lean mass** | **Knee OA** | rs10471339 | G | C | -0.011 | 0.0157 | 1.45E-08 | 0.0319 |
| **Appendicular lean mass** | **Knee OA** | rs1047891 | A | C | 0.0233 | 0.0471 | 5.70E-31 | 2.74E-08 |
| **Appendicular lean mass** | **Knee OA** | rs1063582 | G | T | -0.0185 | -0.0171 | 1.12E-16 | 0.04094 |
| **Appendicular lean mass** | **Knee OA** | rs10776560 | T | C | -0.0157 | -0.0054 | 7.88E-17 | 0.445 |
| **Appendicular lean mass** | **Knee OA** | rs10822117 | G | A | -0.0176 | 0.0163 | 4.24E-15 | 0.05157 |
| **Appendicular lean mass** | **Knee OA** | rs10845408 | T | C | 0.0255 | 0.0173 | 3.25E-38 | 0.01939 |
| **Appendicular lean mass** | **Knee OA** | rs10858246 | C | G | -0.0188 | 0.0119 | 2.35E-20 | 0.121 |
| **Appendicular lean mass** | **Knee OA** | rs10864899 | G | A | -0.0112 | -7.00E-04 | 3.85E-09 | 0.9402 |
| **Appendicular lean mass** | **Knee OA** | rs11014285 | A | G | 0.0342 | 0.0179 | 2.89E-40 | 0.06929 |
| **Appendicular lean mass** | **Knee OA** | rs11121615 | T | C | -0.0202 | -6.00E-04 | 3.32E-23 | 0.943 |
| **Appendicular lean mass** | **Knee OA** | rs111365325 | T | C | -0.0271 | -0.0207 | 1.12E-33 | 0.01449 |
| **Appendicular lean mass** | **Knee OA** | rs11178643 | T | A | 0.0109 | -0.009 | 4.44E-08 | 0.4201 |
| **Appendicular lean mass** | **Knee OA** | rs11191208 | A | G | 0.0147 | 0.009 | 3.69E-10 | 0.3277 |
| **Appendicular lean mass** | **Knee OA** | rs11198591 | A | G | 0.0148 | 0.0059 | 4.91E-14 | 0.4353 |
| **Appendicular lean mass** | **Knee OA** | rs11210892 | A | G | 0.0118 | -0.005 | 3.57E-09 | 0.5055 |
| **Appendicular lean mass** | **Knee OA** | rs11217863 | A | G | -0.0268 | 0.0096 | 1.06E-19 | 0.3753 |
| **Appendicular lean mass** | **Knee OA** | rs112537273 | C | T | -0.0212 | 0.0012 | 3.34E-21 | 0.8875 |
| **Appendicular lean mass** | **Knee OA** | rs11260035 | A | G | 0.015 | 0.0051 | 1.86E-12 | 0.522 |
| **Appendicular lean mass** | **Knee OA** | rs113671109 | C | T | -0.015 | -0.0061 | 4.23E-11 | 0.4752 |
| **Appendicular lean mass** | **Knee OA** | rs115010283 | C | G | 0.034 | -0.0308 | 2.35E-63 | 0.005403 |
| **Appendicular lean mass** | **Knee OA** | rs11562101 | T | A | 0.0115 | -0.0197 | 9.39E-09 | 0.07686 |
| **Appendicular lean mass** | **Knee OA** | rs11580040 | G | A | 0.0325 | 0.0144 | 6.76E-21 | 0.2778 |
| **Appendicular lean mass** | **Knee OA** | rs11590254 | T | A | 0.0186 | -0.0046 | 4.34E-20 | 0.5427 |
| **Appendicular lean mass** | **Knee OA** | rs115912456 | G | A | 0.0577 | -0.0272 | 3.69E-34 | 0.1233 |
| **Appendicular lean mass** | **Knee OA** | rs11605297 | A | G | 0.0146 | -5.00E-04 | 8.03E-11 | 0.9488 |
| **Appendicular lean mass** | **Knee OA** | rs11612462 | G | T | 0.015 | 0.0109 | 2.55E-09 | 0.246 |
| **Appendicular lean mass** | **Knee OA** | rs11629593 | G | T | -0.0109 | 0.0021 | 4.42E-08 | 0.8355 |
| **Appendicular lean mass** | **Knee OA** | rs11672848 | T | C | -0.0171 | 0.0118 | 7.73E-19 | 0.1888 |
| **Appendicular lean mass** | **Knee OA** | rs117068593 | T | C | 0.0403 | -0.0189 | 8.83E-62 | 0.05077 |
| **Appendicular lean mass** | **Knee OA** | rs11721522 | G | A | 0.0106 | -0.0023 | 4.03E-08 | 0.7507 |
| **Appendicular lean mass** | **Knee OA** | rs11727162 | T | C | -0.017 | -0.0066 | 2.15E-19 | 0.3487 |
| **Appendicular lean mass** | **Knee OA** | rs11867855 | T | G | -0.0132 | -7.00E-04 | 2.93E-09 | 0.9308 |
| **Appendicular lean mass** | **Knee OA** | rs12051245 | C | T | 0.0299 | -0.0106 | 2.56E-40 | 0.2095 |
| **Appendicular lean mass** | **Knee OA** | rs12150907 | A | G | -0.0219 | -0.0162 | 7.22E-20 | 0.07551 |
| **Appendicular lean mass** | **Knee OA** | rs12188208 | C | A | -0.0195 | -0.0132 | 1.40E-18 | 0.1188 |
| **Appendicular lean mass** | **Knee OA** | rs12347137 | C | A | -0.046 | -0.0243 | 9.80E-85 | 0.005513 |
| **Appendicular lean mass** | **Knee OA** | rs12517711 | C | T | -0.0147 | -0.0104 | 2.79E-14 | 0.1689 |
| **Appendicular lean mass** | **Knee OA** | rs12541381 | A | G | -0.0319 | -0.0169 | 2.81E-49 | 0.04576 |
| **Appendicular lean mass** | **Knee OA** | rs12563442 | C | T | 0.0122 | -0.0179 | 9.93E-09 | 0.02397 |
| **Appendicular lean mass** | **Knee OA** | rs12655296 | T | C | -0.011 | -0.0039 | 1.62E-08 | 0.5981 |
| **Appendicular lean mass** | **Knee OA** | rs12702693 | T | C | 0.0173 | -0.0063 | 6.56E-20 | 0.3712 |
| **Appendicular lean mass** | **Knee OA** | rs12724708 | T | A | 0.0243 | -0.0161 | 1.66E-35 | 0.06098 |
| **Appendicular lean mass** | **Knee OA** | rs1290786 | T | C | -0.0143 | -8.00E-04 | 7.14E-14 | 0.9102 |
| **Appendicular lean mass** | **Knee OA** | rs12943867 | A | G | 0.0184 | 0.0049 | 7.57E-20 | 0.571 |
| **Appendicular lean mass** | **Knee OA** | rs13391980 | A | G | -0.0225 | 0.0024 | 7.60E-15 | 0.8243 |
| **Appendicular lean mass** | **Knee OA** | rs1341215 | A | G | 0.0229 | 0.0063 | 6.32E-17 | 0.5347 |
| **Appendicular lean mass** | **Knee OA** | rs143554698 | T | C | -0.0257 | -0.0366 | 3.39E-21 | 0.004668 |
| **Appendicular lean mass** | **Knee OA** | rs1472852 | A | C | -0.0638 | 0.0032 | 8.22E-135 | 0.7481 |
| **Appendicular lean mass** | **Knee OA** | rs1478575 | A | T | 0.0312 | -0.0095 | 5.20E-54 | 0.2068 |
| **Appendicular lean mass** | **Knee OA** | rs14976 | T | C | 0.0144 | -2.00E-04 | 1.43E-12 | 0.9833 |
| **Appendicular lean mass** | **Knee OA** | rs1514134 | C | T | -0.0114 | -0.0062 | 3.57E-09 | 0.3878 |
| **Appendicular lean mass** | **Knee OA** | rs1556659 | T | C | 0.0163 | 0.0235 | 7.19E-17 | 0.002164 |
| **Appendicular lean mass** | **Knee OA** | rs17197114 | C | T | 0.0177 | 0.0187 | 1.54E-12 | 0.05544 |
| **Appendicular lean mass** | **Knee OA** | rs17278379 | C | T | 0.0226 | 0.0136 | 2.40E-15 | 0.2147 |
| **Appendicular lean mass** | **Knee OA** | rs173135 | T | C | -0.0341 | -0.0014 | 3.25E-30 | 0.9033 |
| **Appendicular lean mass** | **Knee OA** | rs17478946 | G | A | -0.0192 | -0.0082 | 9.98E-21 | 0.2998 |
| **Appendicular lean mass** | **Knee OA** | rs1880318 | A | G | 0.0147 | -0.0063 | 6.86E-10 | 0.4812 |
| **Appendicular lean mass** | **Knee OA** | rs2070598 | A | G | 0.0204 | 0.0085 | 6.36E-27 | 0.229 |
| **Appendicular lean mass** | **Knee OA** | rs2071450 | T | C | -0.0174 | 0.0044 | 8.85E-19 | 0.5501 |
| **Appendicular lean mass** | **Knee OA** | rs2089111 | G | C | -0.0172 | -0.0268 | 1.73E-15 | 0.001067 |
| **Appendicular lean mass** | **Knee OA** | rs2101017 | T | C | -0.0223 | 0.0038 | 1.44E-15 | 0.7093 |
| **Appendicular lean mass** | **Knee OA** | rs2142331 | T | C | -0.0165 | -0.0147 | 1.38E-17 | 0.04187 |
| **Appendicular lean mass** | **Knee OA** | rs2188805 | C | A | 0.0114 | 0.0027 | 2.00E-08 | 0.7266 |
| **Appendicular lean mass** | **Knee OA** | rs2209098 | C | T | 0.024 | 0.0125 | 1.73E-32 | 0.09848 |
| **Appendicular lean mass** | **Knee OA** | rs2230033 | A | G | -0.0265 | 0.0025 | 3.49E-43 | 0.7237 |
| **Appendicular lean mass** | **Knee OA** | rs2236096 | C | T | 0.018 | 0.0177 | 1.29E-15 | 0.1216 |
| **Appendicular lean mass** | **Knee OA** | rs2237485 | A | G | 0.0191 | -0.0048 | 3.73E-17 | 0.5616 |
| **Appendicular lean mass** | **Knee OA** | rs2268718 | T | C | 0.0141 | 0.0102 | 3.24E-11 | 0.1988 |
| **Appendicular lean mass** | **Knee OA** | rs2289629 | A | G | -0.0148 | -2.00E-04 | 8.02E-14 | 0.9837 |
| **Appendicular lean mass** | **Knee OA** | rs2347808 | A | G | -0.0125 | -0.0055 | 5.79E-11 | 0.4379 |
| **Appendicular lean mass** | **Knee OA** | rs244711 | T | C | 0.0279 | 0.0122 | 1.54E-37 | 0.1746 |
| **Appendicular lean mass** | **Knee OA** | rs2569888 | A | G | 0.0133 | -0.018 | 2.29E-09 | 0.03251 |
| **Appendicular lean mass** | **Knee OA** | rs2578565 | T | C | -0.0141 | -0.0094 | 1.37E-12 | 0.203 |
| **Appendicular lean mass** | **Knee OA** | rs2592208 | A | C | -0.0124 | -0.002 | 5.52E-11 | 0.7718 |
| **Appendicular lean mass** | **Knee OA** | rs2648725 | A | T | 0.0165 | 0.0095 | 8.20E-13 | 0.3081 |
| **Appendicular lean mass** | **Knee OA** | rs2663126 | A | G | -0.0139 | 0.005 | 1.36E-11 | 0.5221 |
| **Appendicular lean mass** | **Knee OA** | rs2754255 | G | A | -0.0153 | 0.0034 | 1.05E-11 | 0.6873 |
| **Appendicular lean mass** | **Knee OA** | rs2763263 | A | T | -0.017 | -0.0014 | 1.37E-14 | 0.8633 |
| **Appendicular lean mass** | **Knee OA** | rs2788213 | A | G | 0.0123 | 0.0056 | 3.80E-09 | 0.4661 |
| **Appendicular lean mass** | **Knee OA** | rs2789365 | T | C | -0.0145 | -0.0121 | 1.14E-14 | 0.08687 |
| **Appendicular lean mass** | **Knee OA** | rs28379706 | C | T | 0.0114 | 0.0151 | 4.49E-09 | 0.03902 |
| **Appendicular lean mass** | **Knee OA** | rs28485212 | T | C | -0.0188 | 0.0128 | 1.24E-12 | 0.286 |
| **Appendicular lean mass** | **Knee OA** | rs28529055 | T | G | -0.0147 | 0.0042 | 1.88E-14 | 0.6636 |
| **Appendicular lean mass** | **Knee OA** | rs28529426 | T | C | -0.0168 | -0.0232 | 5.82E-11 | 0.01816 |
| **Appendicular lean mass** | **Knee OA** | rs28678024 | G | A | -0.0119 | -0.0162 | 1.90E-08 | 0.04092 |
| **Appendicular lean mass** | **Knee OA** | rs2871865 | G | C | -0.0493 | 0.0289 | 3.40E-62 | 0.0119 |
| **Appendicular lean mass** | **Knee OA** | rs2871960 | C | A | 0.0469 | 0.008 | 2.17E-135 | 0.2613 |
| **Appendicular lean mass** | **Knee OA** | rs2923411 | C | T | 0.0127 | 0.0198 | 4.77E-11 | 0.005834 |
| **Appendicular lean mass** | **Knee OA** | rs2925155 | T | C | -0.015 | -0.0195 | 5.47E-12 | 0.01679 |
| **Appendicular lean mass** | **Knee OA** | rs2978362 | T | C | 0.0106 | 0.0062 | 2.85E-08 | 0.3962 |
| **Appendicular lean mass** | **Knee OA** | rs3116194 | A | T | -0.0295 | 0.0386 | 8.29E-21 | 0.001576 |
| **Appendicular lean mass** | **Knee OA** | rs331917 | G | A | -0.0127 | -0.0015 | 3.54E-11 | 0.8349 |
| **Appendicular lean mass** | **Knee OA** | rs336630 | T | C | -0.0106 | -0.0088 | 2.90E-08 | 0.2427 |
| **Appendicular lean mass** | **Knee OA** | rs34312629 | G | C | -0.017 | 0.0026 | 2.12E-15 | 0.7505 |
| **Appendicular lean mass** | **Knee OA** | rs34338597 | G | A | -0.0112 | -0.0076 | 7.88E-09 | 0.2965 |
| **Appendicular lean mass** | **Knee OA** | rs34345560 | A | G | 0.0219 | 0.0031 | 7.10E-20 | 0.7291 |
| **Appendicular lean mass** | **Knee OA** | rs34517439 | A | C | 0.0421 | 0.0299 | 5.80E-48 | 0.008292 |
| **Appendicular lean mass** | **Knee OA** | rs35073631 | C | T | 0.0112 | 0.0096 | 5.92E-09 | 0.3175 |
| **Appendicular lean mass** | **Knee OA** | rs35288270 | C | T | -0.0328 | 0.0206 | 3.43E-32 | 0.1119 |
| **Appendicular lean mass** | **Knee OA** | rs35732917 | C | T | 0.0204 | 0.0028 | 2.08E-22 | 0.7255 |
| **Appendicular lean mass** | **Knee OA** | rs35756741 | T | C | -0.0378 | -0.0064 | 5.80E-31 | 0.602 |
| **Appendicular lean mass** | **Knee OA** | rs35811052 | G | A | -0.0148 | -0.0058 | 8.84E-12 | 0.4754 |
| **Appendicular lean mass** | **Knee OA** | rs36000545 | G | A | -0.022 | -0.0128 | 2.56E-29 | 0.08565 |
| **Appendicular lean mass** | **Knee OA** | rs36048468 | T | C | 0.0254 | -1.00E-04 | 9.34E-28 | 0.9948 |
| **Appendicular lean mass** | **Knee OA** | rs36226649 | C | T | 0.0485 | 0.0233 | 3.05E-37 | 0.1055 |
| **Appendicular lean mass** | **Knee OA** | rs3782232 | A | G | -0.0339 | -0.0177 | 2.41E-20 | 0.2095 |
| **Appendicular lean mass** | **Knee OA** | rs3828729 | G | A | -0.016 | 0.003 | 4.67E-15 | 0.6933 |
| **Appendicular lean mass** | **Knee OA** | rs40270 | C | A | 0.0151 | 0.0121 | 1.90E-11 | 0.1426 |
| **Appendicular lean mass** | **Knee OA** | rs42039 | T | C | 0.0481 | 0.0051 | 3.53E-106 | 0.5346 |
| **Appendicular lean mass** | **Knee OA** | rs4282339 | A | G | -0.0311 | -3.00E-04 | 6.16E-41 | 0.9767 |
| **Appendicular lean mass** | **Knee OA** | rs4287835 | C | T | 0.0147 | -0.0071 | 9.94E-15 | 0.3159 |
| **Appendicular lean mass** | **Knee OA** | rs4360494 | C | G | -0.0198 | 0.0088 | 7.88E-26 | 0.2244 |
| **Appendicular lean mass** | **Knee OA** | rs45474992 | T | C | -0.0617 | 0.0118 | 2.17E-33 | 0.5503 |
| **Appendicular lean mass** | **Knee OA** | rs45528934 | T | C | 0.0262 | -0.0181 | 1.97E-24 | 0.07011 |
| **Appendicular lean mass** | **Knee OA** | rs4640244 | G | A | -0.02 | 0.0017 | 3.81E-25 | 0.8088 |
| **Appendicular lean mass** | **Knee OA** | rs4682483 | A | G | -0.0165 | -0.0162 | 2.65E-10 | 0.08846 |
| **Appendicular lean mass** | **Knee OA** | rs4735761 | C | A | 0.0331 | 0.0135 | 3.66E-56 | 0.08413 |
| **Appendicular lean mass** | **Knee OA** | rs4752689 | A | G | 0.0205 | 0.0105 | 1.36E-26 | 0.1592 |
| **Appendicular lean mass** | **Knee OA** | rs4752829 | A | G | 0.0262 | 0.0293 | 5.90E-36 | 3.22E-04 |
| **Appendicular lean mass** | **Knee OA** | rs4847378 | T | G | 0.0136 | 0.0019 | 1.64E-12 | 0.792 |
| **Appendicular lean mass** | **Knee OA** | rs4870941 | C | G | -0.0297 | -0.0099 | 1.09E-39 | 0.2397 |
| **Appendicular lean mass** | **Knee OA** | rs4932439 | G | A | -0.0151 | -0.0044 | 1.43E-09 | 0.6267 |
| **Appendicular lean mass** | **Knee OA** | rs496783 | G | A | -0.0124 | -0.0126 | 8.13E-11 | 0.09175 |
| **Appendicular lean mass** | **Knee OA** | rs4976262 | C | T | -0.0245 | 0.0223 | 4.37E-33 | 0.003035 |
| **Appendicular lean mass** | **Knee OA** | rs55758152 | A | G | 0.0145 | 0.004 | 1.05E-12 | 0.6576 |
| **Appendicular lean mass** | **Knee OA** | rs55852614 | C | T | -0.0393 | -0.0095 | 3.29E-73 | 0.2484 |
| **Appendicular lean mass** | **Knee OA** | rs56112295 | T | C | 0.0154 | -0.0216 | 1.12E-10 | 0.05136 |
| **Appendicular lean mass** | **Knee OA** | rs568267 | T | C | 0.0122 | 0.0056 | 2.23E-08 | 0.4955 |
| **Appendicular lean mass** | **Knee OA** | rs57059662 | C | T | 0.0118 | 0.002 | 5.63E-09 | 0.7863 |
| **Appendicular lean mass** | **Knee OA** | rs57513571 | T | C | -0.0191 | -0.0269 | 7.02E-16 | 0.002332 |
| **Appendicular lean mass** | **Knee OA** | rs591668 | A | G | -0.0174 | 0.0059 | 2.00E-19 | 0.4169 |
| **Appendicular lean mass** | **Knee OA** | rs599004 | T | C | -0.0157 | -0.0119 | 6.53E-14 | 0.1338 |
| **Appendicular lean mass** | **Knee OA** | rs59950280 | A | G | -0.0254 | -0.0105 | 7.32E-36 | 0.1817 |
| **Appendicular lean mass** | **Knee OA** | rs59985551 | T | C | -0.0313 | -0.0281 | 2.43E-44 | 7.11E-04 |
| **Appendicular lean mass** | **Knee OA** | rs604723 | C | T | -0.0166 | -0.0046 | 8.16E-15 | 0.5574 |
| **Appendicular lean mass** | **Knee OA** | rs6142059 | C | T | 0.0116 | -0.0035 | 1.19E-09 | 0.6168 |
| **Appendicular lean mass** | **Knee OA** | rs61919240 | A | T | 0.0137 | 0.0021 | 9.77E-12 | 0.787 |
| **Appendicular lean mass** | **Knee OA** | rs62033029 | A | G | -0.0141 | 0.01 | 1.73E-09 | 0.2577 |
| **Appendicular lean mass** | **Knee OA** | rs62106258 | C | T | -0.0504 | -0.0839 | 6.45E-31 | 3.88E-06 |
| **Appendicular lean mass** | **Knee OA** | rs62143873 | A | G | -0.0115 | 0.0014 | 1.19E-09 | 0.8515 |
| **Appendicular lean mass** | **Knee OA** | rs62466110 | C | T | -0.0371 | -0.0097 | 5.74E-20 | 0.5741 |
| **Appendicular lean mass** | **Knee OA** | rs62501195 | C | A | -0.0198 | -0.0158 | 8.11E-15 | 0.09896 |
| **Appendicular lean mass** | **Knee OA** | rs62515437 | T | G | 0.0369 | 0.0139 | 8.79E-60 | 0.09993 |
| **Appendicular lean mass** | **Knee OA** | rs6582398 | T | C | 0.014 | 0.0106 | 1.14E-12 | 0.2116 |
| **Appendicular lean mass** | **Knee OA** | rs6675858 | T | C | -0.0137 | -0.0017 | 2.40E-09 | 0.8388 |
| **Appendicular lean mass** | **Knee OA** | rs6738207 | A | G | 0.0127 | 9.00E-04 | 4.14E-11 | 0.8952 |
| **Appendicular lean mass** | **Knee OA** | rs67527161 | C | T | -0.0182 | 0.0043 | 5.79E-15 | 0.6233 |
| **Appendicular lean mass** | **Knee OA** | rs67551338 | T | C | 0.0576 | 0.0187 | 1.04E-47 | 0.203 |
| **Appendicular lean mass** | **Knee OA** | rs67716382 | C | G | 0.0226 | -0.0125 | 1.65E-23 | 0.1443 |
| **Appendicular lean mass** | **Knee OA** | rs6789000 | T | G | 0.0121 | 0.0178 | 1.61E-09 | 0.1066 |
| **Appendicular lean mass** | **Knee OA** | rs68049170 | A | G | -0.0259 | 0.0033 | 2.69E-34 | 0.6722 |
| **Appendicular lean mass** | **Knee OA** | rs6821305 | C | A | 0.0204 | 0.0151 | 3.15E-26 | 0.05602 |
| **Appendicular lean mass** | **Knee OA** | rs684905 | T | C | -0.0118 | -5.00E-04 | 7.10E-10 | 0.9598 |
| **Appendicular lean mass** | **Knee OA** | rs6849302 | G | A | 0.0155 | -0.0026 | 7.11E-11 | 0.7693 |
| **Appendicular lean mass** | **Knee OA** | rs6874142 | G | T | 0.0288 | 0.0296 | 5.15E-20 | 0.03673 |
| **Appendicular lean mass** | **Knee OA** | rs700677 | A | C | 0.0173 | 0.0193 | 1.13E-18 | 0.01251 |
| **Appendicular lean mass** | **Knee OA** | rs7014590 | C | T | -0.0228 | 0.0061 | 4.48E-26 | 0.447 |
| **Appendicular lean mass** | **Knee OA** | rs702886 | G | A | 0.012 | 0.0061 | 1.11E-09 | 0.409 |
| **Appendicular lean mass** | **Knee OA** | rs7129320 | A | G | -0.0389 | -0.0212 | 7.29E-53 | 0.02909 |
| **Appendicular lean mass** | **Knee OA** | rs71414738 | T | C | 0.015 | 1.00E-04 | 1.00E-09 | 0.9926 |
| **Appendicular lean mass** | **Knee OA** | rs718603 | T | C | 0.0131 | -0.0015 | 6.68E-10 | 0.8447 |
| **Appendicular lean mass** | **Knee OA** | rs7229520 | A | G | -0.0224 | -0.0028 | 9.21E-29 | 0.7089 |
| **Appendicular lean mass** | **Knee OA** | rs72656010 | C | T | -0.0668 | -0.0315 | 7.31E-126 | 0.002584 |
| **Appendicular lean mass** | **Knee OA** | rs72721979 | G | T | -0.0229 | 0.03 | 2.20E-17 | 0.003888 |
| **Appendicular lean mass** | **Knee OA** | rs72801843 | A | T | 0.0313 | 0.002 | 8.83E-52 | 0.7946 |
| **Appendicular lean mass** | **Knee OA** | rs72841270 | G | T | 0.0294 | 0.0084 | 2.25E-26 | 0.4171 |
| **Appendicular lean mass** | **Knee OA** | rs72894003 | T | C | -0.0423 | 0.0027 | 1.90E-28 | 0.8713 |
| **Appendicular lean mass** | **Knee OA** | rs7301341 | C | T | -0.0255 | 0.0181 | 9.27E-37 | 0.02006 |
| **Appendicular lean mass** | **Knee OA** | rs73052033 | C | T | -0.0151 | -0.0321 | 4.79E-10 | 5.20E-04 |
| **Appendicular lean mass** | **Knee OA** | rs7320878 | A | G | -0.015 | 8.00E-04 | 1.34E-14 | 0.9267 |
| **Appendicular lean mass** | **Knee OA** | rs7328187 | G | T | 0.0116 | 0.0105 | 1.19E-09 | 0.1393 |
| **Appendicular lean mass** | **Knee OA** | rs73384223 | C | T | -0.0205 | -0.0132 | 1.27E-17 | 0.1458 |
| **Appendicular lean mass** | **Knee OA** | rs73413540 | T | C | -0.0124 | -0.0078 | 4.42E-08 | 0.3742 |
| **Appendicular lean mass** | **Knee OA** | rs73696333 | G | C | 0.0191 | 0.0137 | 3.20E-15 | 0.16 |
| **Appendicular lean mass** | **Knee OA** | rs7418410 | T | C | 0.0155 | -0.0215 | 5.64E-16 | 0.002581 |
| **Appendicular lean mass** | **Knee OA** | rs75022676 | A | G | -0.0163 | -0.0017 | 2.84E-12 | 0.8483 |
| **Appendicular lean mass** | **Knee OA** | rs75702986 | A | G | -0.0163 | 0.0015 | 3.14E-11 | 0.8746 |
| **Appendicular lean mass** | **Knee OA** | rs7598430 | T | C | -0.016 | -0.0212 | 1.37E-17 | 0.002629 |
| **Appendicular lean mass** | **Knee OA** | rs7610055 | A | G | -0.0373 | 0.0093 | 3.55E-38 | 0.3781 |
| **Appendicular lean mass** | **Knee OA** | rs76364830 | A | G | -0.0471 | 8.00E-04 | 2.70E-33 | 0.9615 |
| **Appendicular lean mass** | **Knee OA** | rs7689420 | C | T | 0.0466 | 0.0152 | 1.50E-76 | 0.109 |
| **Appendicular lean mass** | **Knee OA** | rs7701233 | C | T | -0.0179 | -0.0145 | 5.12E-21 | 0.08011 |
| **Appendicular lean mass** | **Knee OA** | rs772222 | G | A | 0.0121 | 0.0132 | 1.55E-08 | 0.09893 |
| **Appendicular lean mass** | **Knee OA** | rs7731023 | G | A | 0.0166 | 0.0015 | 3.48E-18 | 0.8394 |
| **Appendicular lean mass** | **Knee OA** | rs7768973 | A | T | -0.024 | -0.0051 | 5.44E-36 | 0.4721 |
| **Appendicular lean mass** | **Knee OA** | rs7828086 | C | T | 0.0135 | 0.0036 | 1.11E-09 | 0.6634 |
| **Appendicular lean mass** | **Knee OA** | rs78766798 | C | T | 0.0319 | 7.00E-04 | 2.61E-20 | 0.9612 |
| **Appendicular lean mass** | **Knee OA** | rs7952436 | T | C | -0.0453 | -0.0444 | 1.62E-39 | 0.00343 |
| **Appendicular lean mass** | **Knee OA** | rs7971536 | A | T | -0.0194 | 9.00E-04 | 1.06E-24 | 0.8972 |
| **Appendicular lean mass** | **Knee OA** | rs8017006 | G | A | 0.0122 | 0.0193 | 2.22E-09 | 0.06404 |
| **Appendicular lean mass** | **Knee OA** | rs8018486 | G | A | -0.0138 | 0.0136 | 1.18E-08 | 0.1243 |
| **Appendicular lean mass** | **Knee OA** | rs8019890 | A | C | 0.025 | -0.0188 | 1.96E-38 | 0.01163 |
| **Appendicular lean mass** | **Knee OA** | rs80295797 | T | C | -0.0198 | -0.0104 | 3.83E-23 | 0.1717 |
| **Appendicular lean mass** | **Knee OA** | rs8084413 | A | G | -0.0127 | 0.0122 | 3.25E-11 | 0.1017 |
| **Appendicular lean mass** | **Knee OA** | rs861674 | T | A | 0.0128 | 0.0213 | 1.36E-11 | 0.002624 |
| **Appendicular lean mass** | **Knee OA** | rs900399 | G | A | 0.0164 | -4.00E-04 | 1.35E-17 | 0.9549 |
| **Appendicular lean mass** | **Knee OA** | rs905938 | C | T | 0.0394 | 0.0289 | 8.43E-77 | 3.85E-04 |
| **Appendicular lean mass** | **Knee OA** | rs9266244 | A | G | -0.0427 | -0.0368 | 1.21E-94 | 3.77E-04 |
| **Appendicular lean mass** | **Knee OA** | rs9343327 | T | A | 0.014 | 0.0115 | 1.09E-13 | 0.1046 |
| **Appendicular lean mass** | **Knee OA** | rs9375188 | T | C | 0.0136 | 0.0052 | 6.80E-13 | 0.4594 |
| **Appendicular lean mass** | **Knee OA** | rs9385002 | T | A | -0.0147 | -0.0023 | 3.26E-11 | 0.7812 |
| **Appendicular lean mass** | **Knee OA** | rs9391254 | T | C | 0.0166 | -0.0044 | 2.10E-16 | 0.5751 |
| **Appendicular lean mass** | **Knee OA** | rs951366 | C | T | 0.0205 | -0.0074 | 9.15E-27 | 0.3036 |
| **Appendicular lean mass** | **Knee OA** | rs9568031 | T | C | -0.0115 | 0.0025 | 3.34E-08 | 0.7431 |
| **Appendicular lean mass** | **Knee OA** | rs9634212 | A | C | 0.0471 | 0.0272 | 8.59E-95 | 0.001255 |
| **Appendicular lean mass** | **Knee OA** | rs9636364 | A | G | 0.011 | 0.0188 | 5.09E-09 | 0.007744 |
| **Appendicular lean mass** | **Knee OA** | rs9809116 | G | A | -0.016 | -0.0015 | 1.31E-16 | 0.8406 |
| **Appendicular lean mass** | **Knee OA** | rs9894577 | A | G | -0.031 | 0.0124 | 1.40E-52 | 0.102 |
| **Hand grip strength (left)** | **Knee OA** | rs10097417 | G | A | -0.0132302 | 0.0148 | 1.90E-11 | 0.1127 |
| **Hand grip strength (left)** | **Knee OA** | rs10176878 | C | T | -0.0129478 | 0.005 | 8.60E-12 | 0.5886 |
| **Hand grip strength (left)** | **Knee OA** | rs10403906 | A | G | -0.0100323 | 0.0016 | 1.50E-11 | 0.8236 |
| **Hand grip strength (left)** | **Knee OA** | rs10786706 | T | C | 0.0100072 | -0.0037 | 1.70E-11 | 0.6091 |
| **Hand grip strength (left)** | **Knee OA** | rs10846071 | T | C | -0.016641 | -0.0194 | 5.30E-28 | 0.007442 |
| **Hand grip strength (left)** | **Knee OA** | rs10934857 | A | G | 0.00928675 | -4.00E-04 | 4.80E-08 | 0.9642 |
| **Hand grip strength (left)** | **Knee OA** | rs10988217 | G | A | -0.00920657 | 0.0111 | 1.70E-09 | 0.127 |
| **Hand grip strength (left)** | **Knee OA** | rs11002322 | T | G | -0.00998924 | -0.0118 | 2.10E-10 | 0.1132 |
| **Hand grip strength (left)** | **Knee OA** | rs11121542 | A | G | -0.0157601 | 0.0032 | 3.00E-12 | 0.7687 |
| **Hand grip strength (left)** | **Knee OA** | rs11642954 | A | G | -0.0117025 | 0.0042 | 4.50E-10 | 0.6361 |
| **Hand grip strength (left)** | **Knee OA** | rs12473732 | T | C | 0.0109869 | -0.0047 | 1.40E-13 | 0.5267 |
| **Hand grip strength (left)** | **Knee OA** | rs12673062 | A | G | -0.0107691 | -0.0086 | 2.60E-09 | 0.3186 |
| **Hand grip strength (left)** | **Knee OA** | rs12790261 | A | C | -0.0251882 | -0.0414 | 1.20E-20 | 0.006286 |
| **Hand grip strength (left)** | **Knee OA** | rs12889267 | G | A | -0.0137394 | 0.0127 | 4.70E-12 | 0.2045 |
| **Hand grip strength (left)** | **Knee OA** | rs12906830 | C | T | 0.0108401 | -0.0033 | 9.30E-13 | 0.6481 |
| **Hand grip strength (left)** | **Knee OA** | rs13091492 | G | A | -0.00847903 | 0.0105 | 3.30E-08 | 0.1489 |
| **Hand grip strength (left)** | **Knee OA** | rs13146142 | C | T | -0.0202031 | 0.0023 | 2.30E-23 | 0.8228 |
| **Hand grip strength (left)** | **Knee OA** | rs13337177 | T | G | -0.0142799 | 0.0065 | 1.60E-13 | 0.5217 |
| **Hand grip strength (left)** | **Knee OA** | rs1556659 | T | C | 0.0162896 | 0.0235 | 2.50E-26 | 0.002164 |
| **Hand grip strength (left)** | **Knee OA** | rs16870531 | T | C | 0.011198 | 0 | 1.30E-10 | 0.9965 |
| **Hand grip strength (left)** | **Knee OA** | rs17282763 | C | T | 0.00893956 | -0.0103 | 4.40E-08 | 0.184 |
| **Hand grip strength (left)** | **Knee OA** | rs17466480 | G | A | -0.0118235 | -0.0244 | 9.80E-15 | 8.58E-04 |
| **Hand grip strength (left)** | **Knee OA** | rs181766 | C | T | 0.00963247 | 3.00E-04 | 1.90E-09 | 0.97 |
| **Hand grip strength (left)** | **Knee OA** | rs1884447 | A | G | 0.00846069 | -0.0151 | 2.30E-08 | 0.03777 |
| **Hand grip strength (left)** | **Knee OA** | rs2359239 | T | C | -0.00881461 | -0.0064 | 6.90E-09 | 0.3713 |
| **Hand grip strength (left)** | **Knee OA** | rs2871865 | G | C | -0.0217979 | 0.0289 | 5.00E-21 | 0.0119 |
| **Hand grip strength (left)** | **Knee OA** | rs2871960 | C | A | 0.0120879 | 0.008 | 5.50E-16 | 0.2613 |
| **Hand grip strength (left)** | **Knee OA** | rs2974438 | A | G | -0.0100735 | 0.0018 | 3.30E-08 | 0.8388 |
| **Hand grip strength (left)** | **Knee OA** | rs35175534 | C | A | -0.0163733 | -0.0069 | 3.60E-12 | 0.7024 |
| **Hand grip strength (left)** | **Knee OA** | rs4121165 | A | G | -0.0114169 | -0.013 | 3.40E-10 | 0.1341 |
| **Hand grip strength (left)** | **Knee OA** | rs4575361 | T | A | -0.0108028 | 0.0094 | 1.60E-11 | 0.2145 |
| **Hand grip strength (left)** | **Knee OA** | rs4621706 | T | C | -0.0117068 | -1.00E-04 | 6.00E-15 | 0.9933 |
| **Hand grip strength (left)** | **Knee OA** | rs4713506 | A | G | -0.0157268 | -0.0158 | 2.00E-20 | 0.07106 |
| **Hand grip strength (left)** | **Knee OA** | rs4962700 | G | C | 0.00904307 | 0.0189 | 3.20E-08 | 0.03737 |
| **Hand grip strength (left)** | **Knee OA** | rs56338231 | G | A | -0.010847 | -0.0177 | 1.70E-10 | 0.0296 |
| **Hand grip strength (left)** | **Knee OA** | rs59116179 | T | C | 0.00857385 | 0.0042 | 2.40E-08 | 0.5804 |
| **Hand grip strength (left)** | **Knee OA** | rs61286123 | C | T | -0.0101041 | 2.00E-04 | 1.20E-08 | 0.9824 |
| **Hand grip strength (left)** | **Knee OA** | rs62081464 | T | C | -0.0098827 | 0.0145 | 2.80E-08 | 0.08597 |
| **Hand grip strength (left)** | **Knee OA** | rs6689375 | T | A | -0.0160169 | -4.00E-04 | 5.30E-17 | 0.9683 |
| **Hand grip strength (left)** | **Knee OA** | rs7197751 | T | G | -0.00946321 | 0.0012 | 1.40E-09 | 0.8692 |
| **Hand grip strength (left)** | **Knee OA** | rs73307079 | C | T | 0.0111025 | 0.0141 | 1.40E-09 | 0.09293 |
| **Hand grip strength (left)** | **Knee OA** | rs755547 | A | G | 0.0165156 | 0.0165 | 3.20E-18 | 0.06884 |
| **Hand grip strength (left)** | **Knee OA** | rs772014 | G | A | -0.0106178 | -0.0029 | 2.70E-12 | 0.7012 |
| **Hand grip strength (left)** | **Knee OA** | rs7856625 | T | C | -0.0111297 | 0.0099 | 2.50E-13 | 0.169 |
| **Hand grip strength (left)** | **Knee OA** | rs7963801 | C | T | -0.0104348 | -0.0081 | 4.60E-12 | 0.2837 |
| **Hand grip strength (left)** | **Knee OA** | rs821100 | G | A | -0.0101977 | 0.0084 | 1.50E-09 | 0.2929 |
| **Hand grip strength (left)** | **Knee OA** | rs9371201 | T | C | -0.00933847 | 0.003 | 3.00E-09 | 0.688 |
| **Hand grip strength (left)** | **Knee OA** | rs9371881 | A | G | 0.00947946 | -0.008 | 9.60E-10 | 0.3045 |
| **Hand grip strength (left)** | **Knee OA** | rs9388769 | A | G | -0.0140821 | -0.01 | 5.10E-19 | 0.1838 |
| **Hand grip strength (left)** | **Knee OA** | rs9611273 | T | C | 0.0107888 | -0.0087 | 4.50E-10 | 0.2994 |
| **Hand grip strength (left)** | **Knee OA** | rs9944324 | G | A | -0.00855272 | 0.0016 | 1.20E-08 | 0.8213 |
| **Hand grip strength (right)** | **Knee OA** | rs10278546 | C | A | 0.0107539 | -0.0129 | 1.10E-08 | 0.1473 |
| **Hand grip strength (right)** | **Knee OA** | rs1043515 | G | A | 0.0138281 | -0.0039 | 2.80E-20 | 0.5833 |
| **Hand grip strength (right)** | **Knee OA** | rs10770125 | G | A | 0.00844132 | -0.003 | 1.40E-08 | 0.6748 |
| **Hand grip strength (right)** | **Knee OA** | rs10798483 | A | G | 0.0145083 | 0.0098 | 2.80E-22 | 0.169 |
| **Hand grip strength (right)** | **Knee OA** | rs10799428 | T | C | -0.0143588 | 0.0023 | 4.60E-14 | 0.797 |
| **Hand grip strength (right)** | **Knee OA** | rs10846071 | T | C | -0.0156575 | -0.0194 | 6.50E-25 | 0.007442 |
| **Hand grip strength (right)** | **Knee OA** | rs11039348 | A | G | -0.00976855 | -0.0103 | 3.90E-10 | 0.1753 |
| **Hand grip strength (right)** | **Knee OA** | rs11243202 | C | T | 0.0116378 | -0.0324 | 6.50E-15 | 5.20E-06 |
| **Hand grip strength (right)** | **Knee OA** | rs1125 | A | G | -0.0100304 | 0.0012 | 1.90E-10 | 0.8752 |
| **Hand grip strength (right)** | **Knee OA** | rs113315602 | C | A | -0.0212911 | 0.0245 | 1.40E-15 | 0.1965 |
| **Hand grip strength (right)** | **Knee OA** | rs113835839 | T | C | -0.00986353 | -0.001 | 1.10E-08 | 0.9071 |
| **Hand grip strength (right)** | **Knee OA** | rs11642954 | A | G | -0.0131918 | 0.0042 | 2.20E-12 | 0.6361 |
| **Hand grip strength (right)** | **Knee OA** | rs12412806 | A | G | -0.00909582 | -0.0077 | 3.00E-08 | 0.3201 |
| **Hand grip strength (right)** | **Knee OA** | rs12452505 | G | C | -0.0144313 | 1.00E-04 | 1.40E-11 | 0.9942 |
| **Hand grip strength (right)** | **Knee OA** | rs12790261 | A | C | -0.0263714 | -0.0414 | 2.00E-22 | 0.006286 |
| **Hand grip strength (right)** | **Knee OA** | rs12823922 | G | A | -0.0114203 | 0.0212 | 1.60E-10 | 0.01323 |
| **Hand grip strength (right)** | **Knee OA** | rs12889267 | G | A | -0.0122874 | 0.0127 | 6.70E-10 | 0.2045 |
| **Hand grip strength (right)** | **Knee OA** | rs13146142 | C | T | -0.0207968 | 0.0023 | 1.40E-24 | 0.8228 |
| **Hand grip strength (right)** | **Knee OA** | rs13169333 | C | T | 0.00929083 | -0.0074 | 4.30E-08 | 0.3499 |
| **Hand grip strength (right)** | **Knee OA** | rs1442883 | A | C | -0.0106189 | -0.0017 | 5.80E-10 | 0.8391 |
| **Hand grip strength (right)** | **Knee OA** | rs1550115 | T | C | 0.0152836 | 0.0032 | 4.50E-19 | 0.6942 |
| **Hand grip strength (right)** | **Knee OA** | rs1556659 | T | C | 0.0175345 | 0.0235 | 3.80E-30 | 0.002164 |
| **Hand grip strength (right)** | **Knee OA** | rs1885690 | A | C | -0.00839964 | 9.00E-04 | 2.80E-08 | 0.9005 |
| **Hand grip strength (right)** | **Knee OA** | rs1952256 | G | A | 0.00976791 | -0.0203 | 4.10E-10 | 0.006159 |
| **Hand grip strength (right)** | **Knee OA** | rs2194747 | G | A | 0.00985126 | 0.0135 | 1.90E-09 | 0.08355 |
| **Hand grip strength (right)** | **Knee OA** | rs2226685 | C | T | 0.0103333 | -0.0045 | 3.10E-09 | 0.5985 |
| **Hand grip strength (right)** | **Knee OA** | rs246181 | T | C | 0.00970432 | -0.0011 | 3.70E-10 | 0.8824 |
| **Hand grip strength (right)** | **Knee OA** | rs248831 | A | G | 0.00988989 | -0.0234 | 8.40E-09 | 0.004426 |
| **Hand grip strength (right)** | **Knee OA** | rs2854152 | G | A | 0.010991 | 0.0015 | 6.00E-12 | 0.8478 |
| **Hand grip strength (right)** | **Knee OA** | rs2871865 | G | C | -0.0236863 | 0.0289 | 1.80E-24 | 0.0119 |
| **Hand grip strength (right)** | **Knee OA** | rs35175534 | C | A | -0.0191822 | -0.0069 | 4.20E-16 | 0.7024 |
| **Hand grip strength (right)** | **Knee OA** | rs35833641 | G | A | 0.00922306 | -0.0176 | 8.50E-09 | 0.02102 |
| **Hand grip strength (right)** | **Knee OA** | rs3848369 | T | C | -0.00953776 | -0.0115 | 4.70E-10 | 0.1135 |
| **Hand grip strength (right)** | **Knee OA** | rs4121165 | A | G | -0.0119792 | -0.013 | 4.80E-11 | 0.1341 |
| **Hand grip strength (right)** | **Knee OA** | rs4549685 | T | C | 0.00971494 | 0.0029 | 8.10E-10 | 0.7 |
| **Hand grip strength (right)** | **Knee OA** | rs4751671 | A | G | 0.0082332 | 0.0118 | 4.10E-08 | 0.1129 |
| **Hand grip strength (right)** | **Knee OA** | rs4752689 | A | G | 0.0087531 | 0.0105 | 6.40E-09 | 0.1592 |
| **Hand grip strength (right)** | **Knee OA** | rs4868110 | T | A | -0.00971069 | -0.0052 | 1.00E-09 | 0.4921 |
| **Hand grip strength (right)** | **Knee OA** | rs4962700 | G | C | 0.00932733 | 0.0189 | 1.20E-08 | 0.03737 |
| **Hand grip strength (right)** | **Knee OA** | rs56074046 | A | G | -0.00903502 | 0.0012 | 4.40E-09 | 0.8733 |
| **Hand grip strength (right)** | **Knee OA** | rs56365901 | G | A | -0.0142618 | -0.0078 | 1.80E-15 | 0.3788 |
| **Hand grip strength (right)** | **Knee OA** | rs600038 | C | T | -0.0103761 | 0.018 | 1.50E-08 | 0.03512 |
| **Hand grip strength (right)** | **Knee OA** | rs62037412 | A | G | 0.00932966 | 0.0021 | 1.90E-09 | 0.7725 |
| **Hand grip strength (right)** | **Knee OA** | rs645144 | C | T | -0.00868793 | -9.00E-04 | 4.30E-08 | 0.9088 |
| **Hand grip strength (right)** | **Knee OA** | rs6473015 | C | A | 0.00959961 | 0.0135 | 5.50E-09 | 0.08262 |
| **Hand grip strength (right)** | **Knee OA** | rs6693567 | T | C | -0.00974656 | -0.011 | 6.30E-09 | 0.1656 |
| **Hand grip strength (right)** | **Knee OA** | rs6693965 | T | G | -0.016251 | 0.0035 | 3.20E-13 | 0.741 |
| **Hand grip strength (right)** | **Knee OA** | rs6792762 | A | G | -0.00911683 | 0.013 | 1.80E-09 | 0.07102 |
| **Hand grip strength (right)** | **Knee OA** | rs721101 | C | T | 0.00946697 | -0.0127 | 1.60E-08 | 0.1309 |
| **Hand grip strength (right)** | **Knee OA** | rs7266065 | A | G | 0.00993881 | -0.0088 | 4.60E-10 | 0.2439 |
| **Hand grip strength (right)** | **Knee OA** | rs7301953 | A | G | -0.0115397 | 0.0099 | 6.40E-13 | 0.1948 |
| **Hand grip strength (right)** | **Knee OA** | rs7790322 | T | C | -0.00860301 | -0.0144 | 1.20E-08 | 0.04332 |
| **Hand grip strength (right)** | **Knee OA** | rs7871404 | G | A | 0.0119345 | 0.0019 | 3.40E-10 | 0.838 |
| **Hand grip strength (right)** | **Knee OA** | rs7963801 | C | T | -0.0112734 | -0.0081 | 8.40E-14 | 0.2837 |
| **Hand grip strength (right)** | **Knee OA** | rs8055199 | A | G | -0.00896262 | 0.0036 | 1.20E-08 | 0.6383 |
| **Hand grip strength (right)** | **Knee OA** | rs9322822 | T | C | 0.0110799 | -0.0082 | 3.50E-12 | 0.28 |
| **Hand grip strength (right)** | **Knee OA** | rs935728 | T | C | 0.00955337 | -3.00E-04 | 1.80E-09 | 0.9644 |
| **Hand grip strength (right)** | **Knee OA** | rs9639938 | G | C | 0.00873031 | -8.00E-04 | 5.20E-09 | 0.9066 |
| **Hand grip strength (right)** | **Knee OA** | rs9652468 | A | G | -0.0125308 | 0.0088 | 3.30E-13 | 0.2714 |
| **Hand grip strength (right)** | **Knee OA** | rs9757079 | T | C | 0.00980223 | 0 | 8.40E-10 | 0.9949 |
| **Hand grip strength (right)** | **Knee OA** | rs9853018 | T | C | 0.0101977 | 0.0057 | 8.80E-12 | 0.4188 |
| **Usual walking pace**  **Usual walking pace**  **Usual walking pace**  **Usual walking pace**  **Usual walking pace**  **Usual walking pace**  **Usual walking pace**  **Usual walking pace**  **Usual walking pace**  **Usual walking pace**  **Usual walking pace**  **Usual walking pace**  **Usual walking pace**  **Usual walking pace**  **Usual walking pace**  **Usual walking pace**  **Usual walking pace**  **Usual walking pace** | **Knee OA**  **Knee OA**  **Knee OA**  **Knee OA**  **Knee OA**  **Knee OA**  **Knee OA**  **Knee OA**  **Knee OA**  **Knee OA**  **Knee OA**  **Knee OA**  **Knee OA**  **Knee OA**  **Knee OA**  **Knee OA**  **Knee OA**  **Knee OA** | rs10828258 | G | A | -0.00932641 | 0.0185 | 6.70E-12 | 0.01637 |
|  |  | rs10862220 | G | T | 0.00840767 | 0.003 | 4.80E-10 | 0.6885 |
|  |  | rs11039324 | A | G | -0.0102105 | -0.0093 | 2.20E-15 | 0.202 |
|  |  | rs11150623 | T | G | -0.00968813 | -0.0018 | 2.40E-13 | 0.8107 |
|  |  | rs113825410 | G | A | -0.00881182 | 0.0329 | 6.40E-09 | 8.50E-05 |
|  |  | rs11682482 | G | T | 0.00801894 | 0.0028 | 3.30E-09 | 0.7098 |
|  |  | rs11732213 | C | T | 0.0091614 | -0.041 | 9.40E-09 | 4.16E-06 |
|  |  | rs11761141 | G | T | -0.00789936 | -8.00E-04 | 5.00E-09 | 0.9197 |
|  |  | rs11848096 | C | T | -0.00750831 | -0.0044 | 9.20E-09 | 0.5447 |
|  |  | rs12461902 | A | G | -0.00801134 | -0.0022 | 3.60E-09 | 0.7716 |
|  |  | rs205262 | G | A | -0.00870949 | 0.0396 | 1.00E-09 | 6.55E-07 |
|  |  | rs2170670 | A | G | -0.00708769 | 0.0132 | 4.70E-08 | 0.06732 |
|  |  | rs2280406 | A | G | -0.00996145 | 0.0209 | 3.40E-15 | 0.003176 |
|  |  | rs2439823 | G | A | -0.0072789 | 1.00E-04 | 1.10E-08 | 0.9877 |
|  |  | rs2602731 | G | A | -0.00766675 | 0.0111 | 2.10E-08 | 0.1508 |
|  |  | rs273512 | T | C | -0.00966507 | 0.0175 | 6.90E-14 | 0.01479 |
|  |  | rs35711462 | G | A | -0.0071659 | 0.0135 | 1.70E-08 | 0.05541 |
|  |  | rs4109292 | A | G | 0.00735336 | -0.0144 | 6.60E-09 | 0.05019 |

Abbreviations: IVs: instrumental variables; MR: Mendelian randomization；OA: osteoarthritis.
