# Supplementary material for: Genetic causality and site-specific relationship between sarcopenia and osteoarthritis: a bidirectional Mendelian randomization study
Source: Front Genet. 2024 Jan 8;14:1340245. doi: 10.3389/fgene.2023.1340245 (PMC10804883; doi:10.3389/fgene.2023.1340245)
Supplement: Supplementary file 3 [file Table3.DOCX]

**Supplementary Table 3. IVs of osteoarthritis for MR analyses of sarcopenia-related traits on osteoarthritis.**

| **Exposure** | **Outcome** | **SNP** | **Effect allele** | **Other allele** | **GWAS Beta** | | **GWAS P** | |
| --- | --- | --- | --- | --- | --- | --- | --- | --- |
|  |  |  |  |  | **Exposure** | **Outcome** | **Exposure** | **Outcome** |
| **All OA** | **Appendicular lean mass** | rs1149620 | T | A | 0.0264 | 5.00E-04 | 2.23E-08 | 0.8105 |
| **All OA** | **Appendicular lean mass** | rs11726937 | G | A | 0.0313 | -0.0022 | 3.48E-09 | 0.3131 |
| **All OA** | **Appendicular lean mass** | rs11749736 | T | C | -0.0293 | 0.0135 | 1.51E-08 | 2.83E-10 |
| **All OA** | **Appendicular lean mass** | rs2061027 | A | G | 0.0257 | -0.0025 | 1.51E-08 | 0.1887 |
| **All OA** | **Appendicular lean mass** | rs2460451 | C | A | -0.0255 | 0.0051 | 3.51E-08 | 0.00858697 |
| **All OA** | **Appendicular lean mass** | rs2820444 | G | A | -0.028 | 0.0161 | 1.33E-08 | 3.20E-15 |
| **All OA** | **Appendicular lean mass** | rs3731695 | T | C | -0.0258 | 0.0066 | 1.53E-08 | 4.68E-04 |
| **All OA** | **Appendicular lean mass** | rs4098282 | G | A | -0.0404 | 0.0049 | 1.39E-10 | 0.0546802 |
| **All OA** | **Appendicular lean mass** | rs4858241 | T | G | 0.0269 | 9.00E-04 | 9.93E-09 | 0.6436 |
| **All OA** | **Appendicular lean mass** | rs6942634 | G | A | 0.0272 | -0.0089 | 2.81E-09 | 3.19E-06 |
| **All OA** | **Appendicular lean mass** | rs696618 | T | G | 0.0309 | -0.0142 | 2.71E-08 | 1.15E-13 |
| **All OA** | **Appendicular lean mass** | rs9445214 | G | T | 0.025 | -0.006 | 4.08E-08 | 0.00160901 |
| **All OA** | **Hand grip strength (left)** | rs10831476 | A | C | 0.0334 | 0.00123773 | 7.77E-09 | 0.52 |
| **All OA** | **Hand grip strength (left)** | rs1149620 | T | A | 0.0264 | -2.19E-04 | 2.23E-08 | 0.88 |
| **All OA** | **Hand grip strength (left)** | rs11726937 | G | A | 0.0313 | -0.00188924 | 3.48E-09 | 0.28 |
| **All OA** | **Hand grip strength (left)** | rs11730727 | T | C | -0.029 | -0.00253267 | 9.78E-10 | 0.1 |
| **All OA** | **Hand grip strength (left)** | rs11749736 | T | C | -0.0293 | 0.00712023 | 1.51E-08 | 2.50E-05 |
| **All OA** | **Hand grip strength (left)** | rs1467246 | C | T | 0.0256 | 0.00246558 | 1.77E-08 | 0.0980009 |
| **All OA** | **Hand grip strength (left)** | rs1913707 | A | G | 0.0328 | -0.0038007 | 1.39E-12 | 0.0129999 |
| **All OA** | **Hand grip strength (left)** | rs1975163 | T | C | 0.0316 | 4.92E-05 | 9.69E-11 | 0.98 |
| **All OA** | **Hand grip strength (left)** | rs2061027 | A | G | 0.0257 | -0.00419273 | 1.51E-08 | 0.00479999 |
| **All OA** | **Hand grip strength (left)** | rs216175 | A | C | 0.0423 | 0.00126897 | 2.74E-12 | 0.52 |
| **All OA** | **Hand grip strength (left)** | rs2460451 | C | A | -0.0255 | 0.0033574 | 3.51E-08 | 0.0259998 |
| **All OA** | **Hand grip strength (left)** | rs2820444 | G | A | -0.028 | 0.00392786 | 1.33E-08 | 0.015 |
| **All OA** | **Hand grip strength (left)** | rs2856821 | T | C | 0.0321 | -0.00182184 | 4.32E-08 | 0.31 |
| **All OA** | **Hand grip strength (left)** | rs3731695 | T | C | -0.0258 | 0.00355239 | 1.53E-08 | 0.017 |
| **All OA** | **Hand grip strength (left)** | rs4098282 | G | A | -0.0404 | 0.00506633 | 1.39E-10 | 0.012 |
| **All OA** | **Hand grip strength (left)** | rs4858241 | T | G | 0.0269 | -0.00344117 | 9.93E-09 | 0.025 |
| **All OA** | **Hand grip strength (left)** | rs4979340 | T | C | -0.0312 | 0.00294068 | 2.19E-09 | 0.0810009 |
| **All OA** | **Hand grip strength (left)** | rs62262106 | A | G | -0.0296 | 0.00268611 | 2.07E-08 | 0.12 |
| **All OA** | **Hand grip strength (left)** | rs6942634 | G | A | 0.0272 | -0.00338258 | 2.81E-09 | 0.0239999 |
| **All OA** | **Hand grip strength (left)** | rs8050136 | C | A | -0.0257 | -0.00185647 | 2.75E-08 | 0.22 |
| **All OA** | **Hand grip strength (left)** | rs9445214 | G | T | 0.025 | -4.62E-04 | 4.08E-08 | 0.760001 |
| **All OA** | **Hand grip strength (right)** | rs10831476 | A | C | 0.0334 | 9.63E-04 | 7.77E-09 | 0.61 |
| **All OA** | **Hand grip strength (right)** | rs1149620 | T | A | 0.0264 | 6.91E-04 | 2.23E-08 | 0.649999 |
| **All OA** | **Hand grip strength (right)** | rs11726937 | G | A | 0.0313 | -0.00244444 | 3.48E-09 | 0.16 |
| **All OA** | **Hand grip strength (right)** | rs11730727 | T | C | -0.029 | -0.00239384 | 9.78E-10 | 0.13 |
| **All OA** | **Hand grip strength (right)** | rs1467246 | C | T | 0.0256 | 0.00253734 | 1.77E-08 | 0.089 |
| **All OA** | **Hand grip strength (right)** | rs1913707 | A | G | 0.0328 | -0.00475114 | 1.39E-12 | 0.00179999 |
| **All OA** | **Hand grip strength (right)** | rs1975163 | T | C | 0.0316 | 0.0019844 | 9.69E-11 | 0.21 |
| **All OA** | **Hand grip strength (right)** | rs2061027 | A | G | 0.0257 | -0.00441165 | 1.51E-08 | 0.00299999 |
| **All OA** | **Hand grip strength (right)** | rs216175 | A | C | 0.0423 | 0.00101156 | 2.74E-12 | 0.61 |
| **All OA** | **Hand grip strength (right)** | rs2460451 | C | A | -0.0255 | 0.00296256 | 3.51E-08 | 0.05 |
| **All OA** | **Hand grip strength (right)** | rs2820444 | G | A | -0.028 | 0.00495599 | 1.33E-08 | 0.00230001 |
| **All OA** | **Hand grip strength (right)** | rs2856821 | T | C | 0.0321 | -0.00245616 | 4.32E-08 | 0.18 |
| **All OA** | **Hand grip strength (right)** | rs3731695 | T | C | -0.0258 | 0.00418694 | 1.53E-08 | 0.005 |
| **All OA** | **Hand grip strength (right)** | rs4098282 | G | A | -0.0404 | 0.0048847 | 1.39E-10 | 0.016 |
| **All OA** | **Hand grip strength (right)** | rs4858241 | T | G | 0.0269 | -8.70E-04 | 9.93E-09 | 0.57 |
| **All OA** | **Hand grip strength (right)** | rs4979340 | T | C | -0.0312 | 0.00458271 | 2.19E-09 | 0.00649995 |
| **All OA** | **Hand grip strength (right)** | rs62262106 | A | G | -0.0296 | 2.08E-04 | 2.07E-08 | 0.9 |
| **All OA** | **Hand grip strength (right)** | rs6942634 | G | A | 0.0272 | -0.00268716 | 2.81E-09 | 0.0729995 |
| **All OA** | **Hand grip strength (right)** | rs8050136 | C | A | -0.0257 | -4.01E-04 | 2.75E-08 | 0.79 |
| **All OA** | **Hand grip strength (right)** | rs9445214 | G | T | 0.025 | -8.07E-04 | 4.08E-08 | 0.59 |
| **All OA** | **Usual walking pace** | rs10831476 | A | C | 0.0334 | 7.33E-04 | 7.77E-09 | 0.649999 |
| **All OA** | **Usual walking pace** | rs1149620 | T | A | 0.0264 | 0.00297175 | 2.23E-08 | 0.021 |
| **All OA** | **Usual walking pace** | rs11726937 | G | A | 0.0313 | -0.00431462 | 3.48E-09 | 0.00359998 |
| **All OA** | **Usual walking pace** | rs11730727 | T | C | -0.029 | 0.00602961 | 9.78E-10 | 5.80E-06 |
| **All OA** | **Usual walking pace** | rs11749736 | T | C | -0.0293 | 0.00331088 | 1.51E-08 | 0.0219999 |
| **All OA** | **Usual walking pace** | rs1467246 | C | T | 0.0256 | -7.22E-04 | 1.77E-08 | 0.57 |
| **All OA** | **Usual walking pace** | rs1498507 | A | C | 0.0282 | 0.00201991 | 1.25E-09 | 0.11 |
| **All OA** | **Usual walking pace** | rs1913707 | A | G | 0.0328 | -0.00372257 | 1.39E-12 | 0.00409996 |
| **All OA** | **Usual walking pace** | rs1975163 | T | C | 0.0316 | -3.21E-04 | 9.69E-11 | 0.81 |
| **All OA** | **Usual walking pace** | rs2061027 | A | G | 0.0257 | -0.00374857 | 1.51E-08 | 0.00309999 |
| **All OA** | **Usual walking pace** | rs2460451 | C | A | -0.0255 | 0.00276955 | 3.51E-08 | 0.0309999 |
| **All OA** | **Usual walking pace** | rs2820444 | G | A | -0.028 | 0.00383647 | 1.33E-08 | 0.00560003 |
| **All OA** | **Usual walking pace** | rs2856821 | T | C | 0.0321 | -0.00367382 | 4.32E-08 | 0.017 |
| **All OA** | **Usual walking pace** | rs3731695 | T | C | -0.0258 | 0.00288079 | 1.53E-08 | 0.0230001 |
| **All OA** | **Usual walking pace** | rs3771501 | A | G | 0.0357 | -0.00578957 | 4.05E-15 | 5.10E-06 |
| **All OA** | **Usual walking pace** | rs4098282 | G | A | -0.0404 | 5.32E-04 | 1.39E-10 | 0.760001 |
| **All OA** | **Usual walking pace** | rs4858241 | T | G | 0.0269 | -0.00437371 | 9.93E-09 | 8.60E-04 |
| **All OA** | **Usual walking pace** | rs4979340 | T | C | -0.0312 | 3.97E-06 | 2.19E-09 | 1 |
| **All OA** | **Usual walking pace** | rs62262106 | A | G | -0.0296 | 0.00699871 | 2.07E-08 | 1.50E-06 |
| **All OA** | **Usual walking pace** | rs6942634 | G | A | 0.0272 | 6.22E-04 | 2.81E-09 | 0.630001 |
| **All OA** | **Usual walking pace** | rs8050136 | C | A | -0.0257 | 0.00974087 | 2.75E-08 | 5.30E-14 |
| **All OA** | **Usual walking pace** | rs9445214 | G | T | 0.025 | -2.79E-04 | 4.08E-08 | 0.83 |
| **All OA** | **Usual walking pace** | rs981819 | C | T | 0.0329 | -0.00688475 | 2.27E-12 | 1.50E-07 |
| **Hand OA** | **Appendicular lean mass** | rs12939002 | C | T | -0.0745 | -0.0079 | 4.90E-08 | 2.79E-04 |
| **Hand OA** | **Appendicular lean mass** | rs28538668 | T | C | -0.0753 | 0.0128 | 2.38E-08 | 1.30E-09 |
| **Hand OA** | **Appendicular lean mass** | rs3771501 | A | G | 0.0777 | 0.0017 | 1.91E-10 | 0.3648 |
| **Hand OA** | **Appendicular lean mass** | rs3993110 | A | C | 0.0823 | -6.00E-04 | 3.75E-11 | 0.7493 |
| **Hand OA** | **Appendicular lean mass** | rs6511707 | G | A | 0.0725 | 0.0209 | 2.13E-08 | 2.16E-26 |
| **Hand OA** | **Appendicular lean mass** | rs7294636 | G | A | -0.0706 | 0.01 | 1.59E-08 | 2.79E-07 |
| **Hand OA** | **Appendicular lean mass** | rs7748189 | G | A | -0.0799 | -0.0014 | 6.13E-09 | 0.5057 |
| **Hand OA** | **Hand grip strength (left)** | rs11071365 | G | A | -0.1036 | 0.00911008 | 7.78E-17 | 3.10E-09 |
| **Hand OA** | **Hand grip strength (left)** | rs12939002 | C | T | -0.0745 | 0.00181122 | 4.90E-08 | 0.29 |
| **Hand OA** | **Hand grip strength (left)** | rs28538668 | T | C | -0.0753 | 0.00660121 | 2.38E-08 | 7.40E-05 |
| **Hand OA** | **Hand grip strength (left)** | rs3771501 | A | G | 0.0777 | -0.0128794 | 1.91E-10 | 5.10E-18 |
| **Hand OA** | **Hand grip strength (left)** | rs3993110 | A | C | 0.0823 | -0.00883017 | 3.75E-11 | 4.90E-09 |
| **Hand OA** | **Hand grip strength (left)** | rs6511707 | G | A | 0.0725 | -0.00145761 | 2.13E-08 | 0.34 |
| **Hand OA** | **Hand grip strength (left)** | rs7294636 | G | A | -0.0706 | 0.0173054 | 1.59E-08 | 1.10E-29 |
| **Hand OA** | **Hand grip strength (left)** | rs7748189 | G | A | -0.0799 | 0.00573356 | 6.13E-09 | 5.60E-04 |
| **Hand OA** | **Hand grip strength (right)** | rs11071365 | G | A | -0.1036 | 0.00948964 | 7.78E-17 | 7.10E-10 |
| **Hand OA** | **Hand grip strength (right)** | rs12939002 | C | T | -0.0745 | 0.00253259 | 4.90E-08 | 0.14 |
| **Hand OA** | **Hand grip strength (right)** | rs28538668 | T | C | -0.0753 | 0.00909598 | 2.38E-08 | 5.00E-08 |
| **Hand OA** | **Hand grip strength (right)** | rs3771501 | A | G | 0.0777 | -0.0138851 | 1.91E-10 | 1.20E-20 |
| **Hand OA** | **Hand grip strength (right)** | rs3993110 | A | C | 0.0823 | -0.00846922 | 3.75E-11 | 2.10E-08 |
| **Hand OA** | **Hand grip strength (right)** | rs6511707 | G | A | 0.0725 | 3.59E-04 | 2.13E-08 | 0.82 |
| **Hand OA** | **Hand grip strength (right)** | rs7294636 | G | A | -0.0706 | 0.0161308 | 1.59E-08 | 6.00E-26 |
| **Hand OA** | **Hand grip strength (right)** | rs7748189 | G | A | -0.0799 | 0.00624847 | 6.13E-09 | 1.70E-04 |
| **Hand OA** | **Usual walking pace** | rs11071365 | G | A | -0.1036 | 0.00161324 | 7.78E-17 | 0.22 |
| **Hand OA** | **Usual walking pace** | rs12939002 | C | T | -0.0745 | -0.00374469 | 4.90E-08 | 0.00980009 |
| **Hand OA** | **Usual walking pace** | rs28538668 | T | C | -0.0753 | 0.00326209 | 2.38E-08 | 0.0219999 |
| **Hand OA** | **Usual walking pace** | rs3771501 | A | G | 0.0777 | -0.00578957 | 1.91E-10 | 5.10E-06 |
| **Hand OA** | **Usual walking pace** | rs3993110 | A | C | 0.0823 | -8.04E-04 | 3.75E-11 | 0.53 |
| **Hand OA** | **Usual walking pace** | rs6511707 | G | A | 0.0725 | -0.00140854 | 2.13E-08 | 0.28 |
| **Hand OA** | **Usual walking pace** | rs7294636 | G | A | -0.0706 | 0.0012942 | 1.59E-08 | 0.32 |
| **Hand OA** | **Usual walking pace** | rs7748189 | G | A | -0.0799 | 0.00241394 | 6.13E-09 | 0.089 |
| **Hip OA** | **Appendicular lean mass** | rs10465114 | G | A | -0.0625 | 0.0098 | 9.04E-09 | 1.44E-05 |
| **Hip OA** | **Appendicular lean mass** | rs10831477 | T | G | 0.0704 | 0.0059 | 1.20E-09 | 0.01555 |
| **Hip OA** | **Appendicular lean mass** | rs10940168 | G | A | 0.0534 | 0.0104 | 7.74E-09 | 7.58E-08 |
| **Hip OA** | **Appendicular lean mass** | rs11049206 | G | C | -0.1143 | -0.0125 | 3.67E-24 | 2.09E-07 |
| **Hip OA** | **Appendicular lean mass** | rs12046389 | A | C | -0.077 | 0.0053 | 3.14E-17 | 0.00494299 |
| **Hip OA** | **Appendicular lean mass** | rs12074699 | C | A | -0.0627 | -0.0019 | 6.50E-11 | 0.3446 |
| **Hip OA** | **Appendicular lean mass** | rs12209223 | C | A | -0.1398 | -0.0192 | 1.88E-22 | 9.92E-10 |
| **Hip OA** | **Appendicular lean mass** | rs1467246 | C | T | 0.0553 | 0.0126 | 8.87E-10 | 2.76E-11 |
| **Hip OA** | **Appendicular lean mass** | rs1498507 | A | C | 0.0734 | 0.0123 | 1.46E-15 | 8.27E-11 |
| **Hip OA** | **Appendicular lean mass** | rs1913707 | A | G | 0.0677 | 0.0048 | 1.82E-13 | 0.01256 |
| **Hip OA** | **Appendicular lean mass** | rs2716212 | A | G | -0.0554 | -0.0154 | 1.86E-09 | 3.20E-15 |
| **Hip OA** | **Appendicular lean mass** | rs3771501 | A | G | 0.0649 | 0.0017 | 6.59E-13 | 0.3648 |
| **Hip OA** | **Appendicular lean mass** | rs4148949 | T | C | -0.0541 | 0.0064 | 4.15E-09 | 9.80E-04 |
| **Hip OA** | **Appendicular lean mass** | rs62578126 | C | T | 0.0626 | 0.0129 | 4.29E-11 | 3.96E-11 |
| **Hip OA** | **Appendicular lean mass** | rs6792369 | C | G | -0.0675 | 4.00E-04 | 3.04E-13 | 0.8501 |
| **Hip OA** | **Appendicular lean mass** | rs67924081 | A | G | 0.0642 | 0.001 | 7.80E-10 | 0.6442 |
| **Hip OA** | **Appendicular lean mass** | rs6908606 | A | G | -0.0688 | 0.0141 | 3.86E-12 | 7.39E-12 |
| **Hip OA** | **Appendicular lean mass** | rs76622165 | C | T | -0.0608 | -0.0019 | 3.56E-08 | 0.4362 |
| **Hip OA** | **Appendicular lean mass** | rs7875152 | C | A | 0.0935 | -0.0019 | 1.07E-12 | 0.481599 |
| **Hip OA** | **Appendicular lean mass** | rs798756 | T | C | -0.0683 | -0.0013 | 2.24E-09 | 0.5764 |
| **Hip OA** | **Appendicular lean mass** | rs8135498 | C | G | -0.0617 | 0.0117 | 8.46E-10 | 3.16E-08 |
| **Hip OA** | **Appendicular lean mass** | rs9835230 | G | A | -0.0638 | -0.0032 | 1.34E-09 | 0.1512 |
| **Hip OA** | **Hand grip strength (left)** | rs10465114 | G | A | -0.0625 | -0.00175183 | 9.04E-09 | 0.32 |
| **Hip OA** | **Hand grip strength (left)** | rs10808583 | G | A | 0.0821 | 0.0031496 | 5.15E-13 | 0.089 |
| **Hip OA** | **Hand grip strength (left)** | rs10831477 | T | G | 0.0704 | 0.0013575 | 1.20E-09 | 0.48 |
| **Hip OA** | **Hand grip strength (left)** | rs10940168 | G | A | 0.0534 | 0.00716986 | 7.74E-09 | 2.40E-06 |
| **Hip OA** | **Hand grip strength (left)** | rs11049206 | G | C | -0.1143 | -0.00339375 | 3.67E-24 | 0.0749998 |
| **Hip OA** | **Hand grip strength (left)** | rs12046389 | A | C | -0.077 | -2.55E-04 | 3.14E-17 | 0.87 |
| **Hip OA** | **Hand grip strength (left)** | rs12074699 | C | A | -0.0627 | -0.00150332 | 6.50E-11 | 0.35 |
| **Hip OA** | **Hand grip strength (left)** | rs12209223 | C | A | -0.1398 | 0.00343052 | 1.88E-22 | 0.16 |
| **Hip OA** | **Hand grip strength (left)** | rs1407243 | C | T | -0.0729 | -0.010345 | 1.71E-15 | 8.90E-12 |
| **Hip OA** | **Hand grip strength (left)** | rs1467246 | C | T | 0.0553 | 0.00246558 | 8.87E-10 | 0.0980009 |
| **Hip OA** | **Hand grip strength (left)** | rs1498507 | A | C | 0.0734 | 0.00723913 | 1.46E-15 | 1.10E-06 |
| **Hip OA** | **Hand grip strength (left)** | rs1913707 | A | G | 0.0677 | -0.0038007 | 1.82E-13 | 0.0129999 |
| **Hip OA** | **Hand grip strength (left)** | rs1926872 | T | C | 0.0707 | -0.00862147 | 5.78E-14 | 3.20E-08 |
| **Hip OA** | **Hand grip strength (left)** | rs1982499 | C | A | 0.0635 | -0.00221307 | 1.95E-11 | 0.16 |
| **Hip OA** | **Hand grip strength (left)** | rs2716212 | A | G | -0.0554 | -0.00390172 | 1.86E-09 | 0.0109999 |
| **Hip OA** | **Hand grip strength (left)** | rs3771501 | A | G | 0.0649 | -0.0128794 | 6.59E-13 | 5.10E-18 |
| **Hip OA** | **Hand grip strength (left)** | rs4073717 | G | T | 0.0672 | 0.00795717 | 2.54E-09 | 1.70E-05 |
| **Hip OA** | **Hand grip strength (left)** | rs4148949 | T | C | -0.0541 | -0.00116306 | 4.15E-09 | 0.44 |
| **Hip OA** | **Hand grip strength (left)** | rs62578126 | C | T | 0.0626 | 0.00269012 | 4.29E-11 | 0.08 |
| **Hip OA** | **Hand grip strength (left)** | rs6792369 | C | G | -0.0675 | 0.00435936 | 3.04E-13 | 0.00470002 |
| **Hip OA** | **Hand grip strength (left)** | rs67924081 | A | G | 0.0642 | 2.55E-04 | 7.80E-10 | 0.88 |
| **Hip OA** | **Hand grip strength (left)** | rs6908606 | A | G | -0.0688 | 0.00177049 | 3.86E-12 | 0.27 |
| **Hip OA** | **Hand grip strength (left)** | rs76622165 | C | T | -0.0608 | -0.00378049 | 3.56E-08 | 0.0449997 |
| **Hip OA** | **Hand grip strength (left)** | rs7875152 | C | A | 0.0935 | 5.45E-04 | 1.07E-12 | 0.8 |
| **Hip OA** | **Hand grip strength (left)** | rs798756 | T | C | -0.0683 | -9.88E-05 | 2.24E-09 | 0.96 |
| **Hip OA** | **Hand grip strength (left)** | rs8135498 | C | G | -0.0617 | -0.00576278 | 8.46E-10 | 4.50E-04 |
| **Hip OA** | **Hand grip strength (left)** | rs9835230 | G | A | -0.0638 | -2.23E-04 | 1.34E-09 | 0.9 |
| **Hip OA** | **Hand grip strength (left)** | rs9940278 | C | T | -0.0543 | -0.00145333 | 1.77E-09 | 0.33 |
| **Hip OA** | **Hand grip strength (right)** | rs10465114 | G | A | -0.0625 | -0.00200381 | 9.04E-09 | 0.26 |
| **Hip OA** | **Hand grip strength (right)** | rs10808583 | G | A | 0.0821 | 0.00367153 | 5.15E-13 | 0.0470002 |
| **Hip OA** | **Hand grip strength (right)** | rs10831477 | T | G | 0.0704 | 0.00106114 | 1.20E-09 | 0.58 |
| **Hip OA** | **Hand grip strength (right)** | rs10940168 | G | A | 0.0534 | 0.0076115 | 7.74E-09 | 5.60E-07 |
| **Hip OA** | **Hand grip strength (right)** | rs11049206 | G | C | -0.1143 | -0.00288439 | 3.67E-24 | 0.13 |
| **Hip OA** | **Hand grip strength (right)** | rs12046389 | A | C | -0.077 | 2.45E-04 | 3.14E-17 | 0.87 |
| **Hip OA** | **Hand grip strength (right)** | rs12074699 | C | A | -0.0627 | -0.00240747 | 6.50E-11 | 0.13 |
| **Hip OA** | **Hand grip strength (right)** | rs12209223 | C | A | -0.1398 | 0.00469451 | 1.88E-22 | 0.0580003 |
| **Hip OA** | **Hand grip strength (right)** | rs1407243 | C | T | -0.0729 | -0.0111261 | 1.71E-15 | 2.30E-13 |
| **Hip OA** | **Hand grip strength (right)** | rs1467246 | C | T | 0.0553 | 0.00253734 | 8.87E-10 | 0.089 |
| **Hip OA** | **Hand grip strength (right)** | rs1498507 | A | C | 0.0734 | 0.00725717 | 1.46E-15 | 1.10E-06 |
| **Hip OA** | **Hand grip strength (right)** | rs1913707 | A | G | 0.0677 | -0.00475114 | 1.82E-13 | 0.00179999 |
| **Hip OA** | **Hand grip strength (right)** | rs1926872 | T | C | 0.0707 | -0.00964073 | 5.78E-14 | 6.50E-10 |
| **Hip OA** | **Hand grip strength (right)** | rs1982499 | C | A | 0.0635 | -0.00313371 | 1.95E-11 | 0.0449997 |
| **Hip OA** | **Hand grip strength (right)** | rs2716212 | A | G | -0.0554 | -0.0042636 | 1.86E-09 | 0.00539995 |
| **Hip OA** | **Hand grip strength (right)** | rs3771501 | A | G | 0.0649 | -0.0138851 | 6.59E-13 | 1.20E-20 |
| **Hip OA** | **Hand grip strength (right)** | rs4073717 | G | T | 0.0672 | 0.00746651 | 2.54E-09 | 5.70E-05 |
| **Hip OA** | **Hand grip strength (right)** | rs4148949 | T | C | -0.0541 | -8.50E-04 | 4.15E-09 | 0.58 |
| **Hip OA** | **Hand grip strength (right)** | rs62578126 | C | T | 0.0626 | 0.00359547 | 4.29E-11 | 0.0189998 |
| **Hip OA** | **Hand grip strength (right)** | rs6792369 | C | G | -0.0675 | 0.00387268 | 3.04E-13 | 0.012 |
| **Hip OA** | **Hand grip strength (right)** | rs67924081 | A | G | 0.0642 | -0.0010273 | 7.80E-10 | 0.54 |
| **Hip OA** | **Hand grip strength (right)** | rs6908606 | A | G | -0.0688 | 6.36E-04 | 3.86E-12 | 0.69 |
| **Hip OA** | **Hand grip strength (right)** | rs76622165 | C | T | -0.0608 | -0.00601377 | 3.56E-08 | 0.0015 |
| **Hip OA** | **Hand grip strength (right)** | rs7875152 | C | A | 0.0935 | 0.00148101 | 1.07E-12 | 0.49 |
| **Hip OA** | **Hand grip strength (right)** | rs798756 | T | C | -0.0683 | 3.72E-04 | 2.24E-09 | 0.84 |
| **Hip OA** | **Hand grip strength (right)** | rs8135498 | C | G | -0.0617 | -0.00520304 | 8.46E-10 | 0.0016 |
| **Hip OA** | **Hand grip strength (right)** | rs9835230 | G | A | -0.0638 | 0.00123832 | 1.34E-09 | 0.48 |
| **Hip OA** | **Hand grip strength (right)** | rs9940278 | C | T | -0.0543 | -3.18E-05 | 1.77E-09 | 0.98 |
| **Hip OA** | **Usual walking pace** | rs10465114 | G | A | -0.0625 | 0.00152606 | 9.04E-09 | 0.31 |
| **Hip OA** | **Usual walking pace** | rs10808583 | G | A | 0.0821 | -0.00215405 | 5.15E-13 | 0.17 |
| **Hip OA** | **Usual walking pace** | rs10831477 | T | G | 0.0704 | 7.88E-04 | 1.20E-09 | 0.630001 |
| **Hip OA** | **Usual walking pace** | rs10940168 | G | A | 0.0534 | 0.00163477 | 7.74E-09 | 0.21 |
| **Hip OA** | **Usual walking pace** | rs12046389 | A | C | -0.077 | 0.00172204 | 3.14E-17 | 0.18 |
| **Hip OA** | **Usual walking pace** | rs12074699 | C | A | -0.0627 | 1.66E-05 | 6.50E-11 | 0.99 |
| **Hip OA** | **Usual walking pace** | rs12209223 | C | A | -0.1398 | 0.00234742 | 1.88E-22 | 0.27 |
| **Hip OA** | **Usual walking pace** | rs1407243 | C | T | -0.0729 | 0.00259899 | 1.71E-15 | 0.0439997 |
| **Hip OA** | **Usual walking pace** | rs1467246 | C | T | 0.0553 | -7.22E-04 | 8.87E-10 | 0.57 |
| **Hip OA** | **Usual walking pace** | rs1913707 | A | G | 0.0677 | -0.00372257 | 1.82E-13 | 0.00409996 |
| **Hip OA** | **Usual walking pace** | rs1926872 | T | C | 0.0707 | -0.00365756 | 5.78E-14 | 0.00589997 |
| **Hip OA** | **Usual walking pace** | rs1982499 | C | A | 0.0635 | -0.00462978 | 1.95E-11 | 5.20E-04 |
| **Hip OA** | **Usual walking pace** | rs2716212 | A | G | -0.0554 | -5.57E-04 | 1.86E-09 | 0.67 |
| **Hip OA** | **Usual walking pace** | rs4073717 | G | T | 0.0672 | 2.18E-04 | 2.54E-09 | 0.89 |
| **Hip OA** | **Usual walking pace** | rs4148949 | T | C | -0.0541 | 0.00478233 | 4.15E-09 | 2.20E-04 |
| **Hip OA** | **Usual walking pace** | rs6792369 | C | G | -0.0675 | 0.00405861 | 3.04E-13 | 0.002 |
| **Hip OA** | **Usual walking pace** | rs67924081 | A | G | 0.0642 | 0.00221578 | 7.80E-10 | 0.12 |
| **Hip OA** | **Usual walking pace** | rs6908606 | A | G | -0.0688 | 0.00172434 | 3.86E-12 | 0.21 |
| **Hip OA** | **Usual walking pace** | rs76622165 | C | T | -0.0608 | 0.00215432 | 3.56E-08 | 0.18 |
| **Hip OA** | **Usual walking pace** | rs7875152 | C | A | 0.0935 | -0.0051151 | 1.07E-12 | 0.00479999 |
| **Hip OA** | **Usual walking pace** | rs8135498 | C | G | -0.0617 | 9.22E-04 | 8.46E-10 | 0.51 |
| **Hip OA** | **Usual walking pace** | rs9835230 | G | A | -0.0638 | -2.93E-04 | 1.34E-09 | 0.84 |
| **Knee OA** | **Appendicular lean mass** | rs10842226 | G | A | -0.0467 | -0.0056 | 3.63E-09 | 0.00358204 |
| **Knee OA** | **Appendicular lean mass** | rs10974438 | A | C | 0.0434 | -0.0067 | 4.89E-09 | 7.67E-04 |
| **Knee OA** | **Appendicular lean mass** | rs11057203 | G | T | -0.0665 | 0.0226 | 3.98E-09 | 7.71E-25 |
| **Knee OA** | **Appendicular lean mass** | rs11704274 | A | G | 0.0512 | 0.0049 | 3.13E-09 | 0.02756 |
| **Knee OA** | **Appendicular lean mass** | rs1563350 | G | A | 0.0444 | -0.0209 | 4.12E-09 | 9.79E-26 |
| **Knee OA** | **Appendicular lean mass** | rs2061027 | A | G | 0.0414 | -0.0025 | 4.49E-09 | 0.1887 |
| **Knee OA** | **Appendicular lean mass** | rs2066928 | A | G | -0.0412 | 1.00E-04 | 1.20E-08 | 0.9415 |
| **Knee OA** | **Appendicular lean mass** | rs34195470 | A | G | -0.0523 | 0.0142 | 3.13E-13 | 1.21E-13 |
| **Knee OA** | **Appendicular lean mass** | rs3764002 | C | T | 0.0508 | -0.028 | 8.90E-10 | 4.47E-39 |
| **Knee OA** | **Appendicular lean mass** | rs3823277 | T | C | -0.0398 | 0.0079 | 4.70E-08 | 5.38E-05 |
| **Knee OA** | **Appendicular lean mass** | rs4380013 | G | A | -0.0562 | 0.0055 | 8.73E-10 | 0.0199701 |
| **Knee OA** | **Appendicular lean mass** | rs4912848 | C | T | -0.0453 | 0.0127 | 9.81E-09 | 2.18E-09 |
| **Knee OA** | **Appendicular lean mass** | rs58973023 | A | T | 0.0555 | -0.0077 | 4.72E-10 | 4.55E-05 |
| **Knee OA** | **Appendicular lean mass** | rs72979233 | A | G | -0.0496 | 0.0143 | 2.54E-09 | 6.88E-11 |
| **Knee OA** | **Appendicular lean mass** | rs737142 | T | C | -0.0473 | 0.0039 | 2.12E-10 | 0.0540605 |
| **Knee OA** | **Appendicular lean mass** | rs7967762 | C | T | -0.057 | -0.0032 | 2.09E-09 | 0.2127 |
| **Knee OA** | **Hand grip strength (left)** | rs1047891 | C | A | -0.0471 | -0.00760568 | 2.74E-08 | 1.80E-06 |
| **Knee OA** | **Hand grip strength (left)** | rs10842226 | G | A | -0.0467 | -0.00605532 | 3.63E-09 | 5.70E-05 |
| **Knee OA** | **Hand grip strength (left)** | rs10974438 | A | C | 0.0434 | -0.00452864 | 4.89E-09 | 0.00359998 |
| **Knee OA** | **Hand grip strength (left)** | rs11629600 | T | A | 0.0432 | 0.0117405 | 1.04E-08 | 2.20E-13 |
| **Knee OA** | **Hand grip strength (left)** | rs11704274 | A | G | 0.0512 | 0.00296799 | 3.13E-09 | 0.0840001 |
| **Knee OA** | **Hand grip strength (left)** | rs1563350 | G | A | 0.0444 | -0.00309995 | 4.12E-09 | 0.05 |
| **Knee OA** | **Hand grip strength (left)** | rs2061027 | A | G | 0.0414 | -0.00419273 | 4.49E-09 | 0.00479999 |
| **Knee OA** | **Hand grip strength (left)** | rs2066928 | A | G | -0.0412 | -5.26E-04 | 1.20E-08 | 0.73 |
| **Knee OA** | **Hand grip strength (left)** | rs34195470 | A | G | -0.0523 | 0.00851166 | 3.13E-13 | 1.40E-08 |
| **Knee OA** | **Hand grip strength (left)** | rs3764002 | C | T | 0.0508 | -0.00263738 | 8.90E-10 | 0.12 |
| **Knee OA** | **Hand grip strength (left)** | rs3823277 | T | C | -0.0398 | 0.00603371 | 4.70E-08 | 8.90E-05 |
| **Knee OA** | **Hand grip strength (left)** | rs3843750 | C | G | 0.0435 | 5.25E-05 | 1.04E-08 | 0.97 |
| **Knee OA** | **Hand grip strength (left)** | rs4380013 | G | A | -0.0562 | 0.010147 | 8.73E-10 | 5.10E-08 |
| **Knee OA** | **Hand grip strength (left)** | rs4523957 | G | T | 0.0502 | 0.00504487 | 6.22E-12 | 0.0012 |
| **Knee OA** | **Hand grip strength (left)** | rs4912848 | C | T | -0.0453 | 0.00652215 | 9.81E-09 | 9.30E-05 |
| **Knee OA** | **Hand grip strength (left)** | rs72979233 | A | G | -0.0496 | 0.0170579 | 2.54E-09 | 4.30E-23 |
| **Knee OA** | **Hand grip strength (left)** | rs737142 | T | C | -0.0473 | 0.00272183 | 2.12E-10 | 0.0850002 |
| **Knee OA** | **Hand grip strength (left)** | rs7967762 | C | T | -0.057 | -7.79E-04 | 2.09E-09 | 0.7 |
| **Knee OA** | **Hand grip strength (left)** | rs9940278 | C | T | -0.058 | -0.00145333 | 3.19E-16 | 0.33 |
| **Knee OA** | **Hand grip strength (right)** | rs1047891 | C | A | -0.0471 | -0.0096867 | 2.74E-08 | 1.30E-09 |
| **Knee OA** | **Hand grip strength (right)** | rs10842226 | G | A | -0.0467 | -0.00616996 | 3.63E-09 | 4.20E-05 |
| **Knee OA** | **Hand grip strength (right)** | rs10974438 | A | C | 0.0434 | -0.00698065 | 4.89E-09 | 7.50E-06 |
| **Knee OA** | **Hand grip strength (right)** | rs11629600 | T | A | 0.0432 | 0.0122512 | 1.04E-08 | 2.10E-14 |
| **Knee OA** | **Hand grip strength (right)** | rs11704274 | A | G | 0.0512 | 0.00364673 | 3.13E-09 | 0.0340001 |
| **Knee OA** | **Hand grip strength (right)** | rs1563350 | G | A | 0.0444 | -0.00449962 | 4.12E-09 | 0.00439997 |
| **Knee OA** | **Hand grip strength (right)** | rs2061027 | A | G | 0.0414 | -0.00441165 | 4.49E-09 | 0.00299999 |
| **Knee OA** | **Hand grip strength (right)** | rs2066928 | A | G | -0.0412 | 9.19E-04 | 1.20E-08 | 0.54 |
| **Knee OA** | **Hand grip strength (right)** | rs34195470 | A | G | -0.0523 | 0.00796946 | 3.13E-13 | 1.10E-07 |
| **Knee OA** | **Hand grip strength (right)** | rs3764002 | C | T | 0.0508 | -0.00326461 | 8.90E-10 | 0.0530005 |
| **Knee OA** | **Hand grip strength (right)** | rs3823277 | T | C | -0.0398 | 0.00537214 | 4.70E-08 | 5.00E-04 |
| **Knee OA** | **Hand grip strength (right)** | rs3843750 | C | G | 0.0435 | 0.00200462 | 1.04E-08 | 0.2 |
| **Knee OA** | **Hand grip strength (right)** | rs4380013 | G | A | -0.0562 | 0.00757589 | 8.73E-10 | 4.90E-05 |
| **Knee OA** | **Hand grip strength (right)** | rs4523957 | G | T | 0.0502 | 0.00228154 | 6.22E-12 | 0.14 |
| **Knee OA** | **Hand grip strength (right)** | rs4912848 | C | T | -0.0453 | 0.00898045 | 9.81E-09 | 7.80E-08 |
| **Knee OA** | **Hand grip strength (right)** | rs72979233 | A | G | -0.0496 | 0.0181802 | 2.54E-09 | 6.00E-26 |
| **Knee OA** | **Hand grip strength (right)** | rs737142 | T | C | -0.0473 | 0.00524033 | 2.12E-10 | 9.40E-04 |
| **Knee OA** | **Hand grip strength (right)** | rs7967762 | C | T | -0.057 | 0.00242538 | 2.09E-09 | 0.24 |
| **Knee OA** | **Hand grip strength (right)** | rs9940278 | C | T | -0.058 | -3.18E-05 | 3.19E-16 | 0.98 |
| **Knee OA** | **Usual walking pace** | rs1047891 | C | A | -0.0471 | 0.00262984 | 2.74E-08 | 0.0530005 |
| **Knee OA** | **Usual walking pace** | rs10842226 | G | A | -0.0467 | 0.00185255 | 3.63E-09 | 0.15 |
| **Knee OA** | **Usual walking pace** | rs10974438 | A | C | 0.0434 | -0.00545424 | 4.89E-09 | 3.90E-05 |
| **Knee OA** | **Usual walking pace** | rs11629600 | T | A | 0.0432 | 0.00262542 | 1.04E-08 | 0.0539995 |
| **Knee OA** | **Usual walking pace** | rs11704274 | A | G | 0.0512 | -0.00179958 | 3.13E-09 | 0.22 |
| **Knee OA** | **Usual walking pace** | rs143384 | A | G | 0.0722 | -0.00785314 | 1.01E-23 | 1.10E-09 |
| **Knee OA** | **Usual walking pace** | rs1563350 | G | A | 0.0444 | -0.00427242 | 4.12E-09 | 0.0015 |
| **Knee OA** | **Usual walking pace** | rs2061027 | A | G | 0.0414 | -0.00374857 | 4.49E-09 | 0.00309999 |
| **Knee OA** | **Usual walking pace** | rs2066928 | A | G | -0.0412 | 0.0013305 | 1.20E-08 | 0.3 |
| **Knee OA** | **Usual walking pace** | rs34195470 | A | G | -0.0523 | 0.00463191 | 3.13E-13 | 2.90E-04 |
| **Knee OA** | **Usual walking pace** | rs3764002 | C | T | 0.0508 | -0.00281348 | 8.90E-10 | 0.051 |
| **Knee OA** | **Usual walking pace** | rs3823277 | T | C | -0.0398 | 0.00149417 | 4.70E-08 | 0.26 |
| **Knee OA** | **Usual walking pace** | rs3843750 | C | G | 0.0435 | -3.19E-04 | 1.04E-08 | 0.81 |
| **Knee OA** | **Usual walking pace** | rs4380013 | G | A | -0.0562 | 5.50E-04 | 8.73E-10 | 0.73 |
| **Knee OA** | **Usual walking pace** | rs4523957 | G | T | 0.0502 | -2.56E-04 | 6.22E-12 | 0.85 |
| **Knee OA** | **Usual walking pace** | rs4912848 | C | T | -0.0453 | 0.00329039 | 9.81E-09 | 0.021 |
| **Knee OA** | **Usual walking pace** | rs72979233 | A | G | -0.0496 | 0.00427144 | 2.54E-09 | 0.00369999 |
| **Knee OA** | **Usual walking pace** | rs737142 | T | C | -0.0473 | 6.66E-04 | 2.12E-10 | 0.62 |
| **Knee OA** | **Usual walking pace** | rs7967762 | C | T | -0.057 | 0.00241526 | 2.09E-09 | 0.17 |
| **Knee OA** | **Usual walking pace** | rs9940278 | C | T | -0.058 | 0.00963675 | 3.19E-16 | 5.30E-14 |

Abbreviations: IVs: instrumental variables; MR: Mendelian randomization；OA: osteoarthritis.
