# Supplementary material for: Genetic causality and site-specific relationship between sarcopenia and osteoarthritis: a bidirectional Mendelian randomization study
Source: Front Genet. 2024 Jan 8;14:1340245. doi: 10.3389/fgene.2023.1340245 (PMC10804883; doi:10.3389/fgene.2023.1340245)
Supplement: Supplementary file 4 [file Table4.DOCX]

**Supplementary Table 4. Statistical power in MR analysis.**

| exposure | outcome | R^2^ | power |
| --- | --- | --- | --- |
| Appendicular lean mass | All OA | 0.014287923 | 99.2% |
| Appendicular lean mass | Hand OA | 0.015752584 | 52.8% |
| Appendicular lean mass | Hip OA | 0.016221837 | 55.2% |
| Appendicular lean mass | Knee OA | 0.017256567 | 100% |
| Hand grip strength (left) | All OA | 0.001551592 | 81.7% |
| Hand grip strength (left) | Hand OA | 0.002129183 | 25.5% |
| Hand grip strength (left) | Hip OA | 0.001723856 | 19.7% |
| Hand grip strength (left) | Knee OA | 0.002157206 | 42.9% |
| Hand grip strength (right) | All OA | 0.001650979 | 58.00% |
| Hand grip strength (right) | Hand OA | 0.002312067 | 42.5% |
| Hand grip strength (right) | Hip OA | 0.002060478 | 30.7% |
| Hand grip strength (right) | Knee OA | 0.002473787 | 3.2% |
| Usual walking pace | All OA | 0.000707049 | 100.00% |
| Usual walking pace | Hand OA | 0.000707049 | 3.1% |
| Usual walking pace | Hip OA | 0.000618666 | 91.9% |
| Usual walking pace | Knee OA | 0.000707049 | 100.00% |
| All OA | Appendicular lean mass | 0.00021108 | 71.1% |
| All OA | Hand grip strength (left) | 0.000374994 | 13.2% |
| All OA | Hand grip strength (right) | 0.000360118 | 10.5% |
| All OA | Usual walking pace | 0.000445324 | 31.9% |
| Hand OA | Appendicular lean mass | 0.000353068 | 3.6% |
| Hand OA | Hand grip strength (left) | 0.000461321 | 31.8% |
| Hand OA | Hand grip strength (right) | 0.000461321 | 32.9% |
| Hand OA | Usual walking pace | 0.000461321 | 4.9% |
| Hip OA | Appendicular lean mass | 0.001193585 | 19.2% |
| Hip OA | Hand grip strength (left) | 0.00153887 | 5.3% |
| Hip OA | Hand grip strength (right) | 0.00153887 | 4.8% |
| Hip OA | Usual walking pace | 0.001145021 | 8.3% |
| Knee OA | Appendicular lean mass | 0.000624813 | 85.1% |
| Knee OA | Hand grip strength (left) | 0.000786329 | 8% |
| Knee OA | Hand grip strength (right) | 0.000786329 | 11.9% |
| Knee OA | Usual walking pace | 0.000909401 | 25.7% |

Abbreviations: OA: osteoarthritis.
