# Supplementary material for: Genetic causality and site-specific relationship between sarcopenia and osteoarthritis: a bidirectional Mendelian randomization study
Source: Front Genet. 2024 Jan 8;14:1340245. doi: 10.3389/fgene.2023.1340245 (PMC10804883; doi:10.3389/fgene.2023.1340245)
Supplement: Supplementary file 5 [file DataSheet1.docx]

**SUPPLEMENTARY FIGURES**


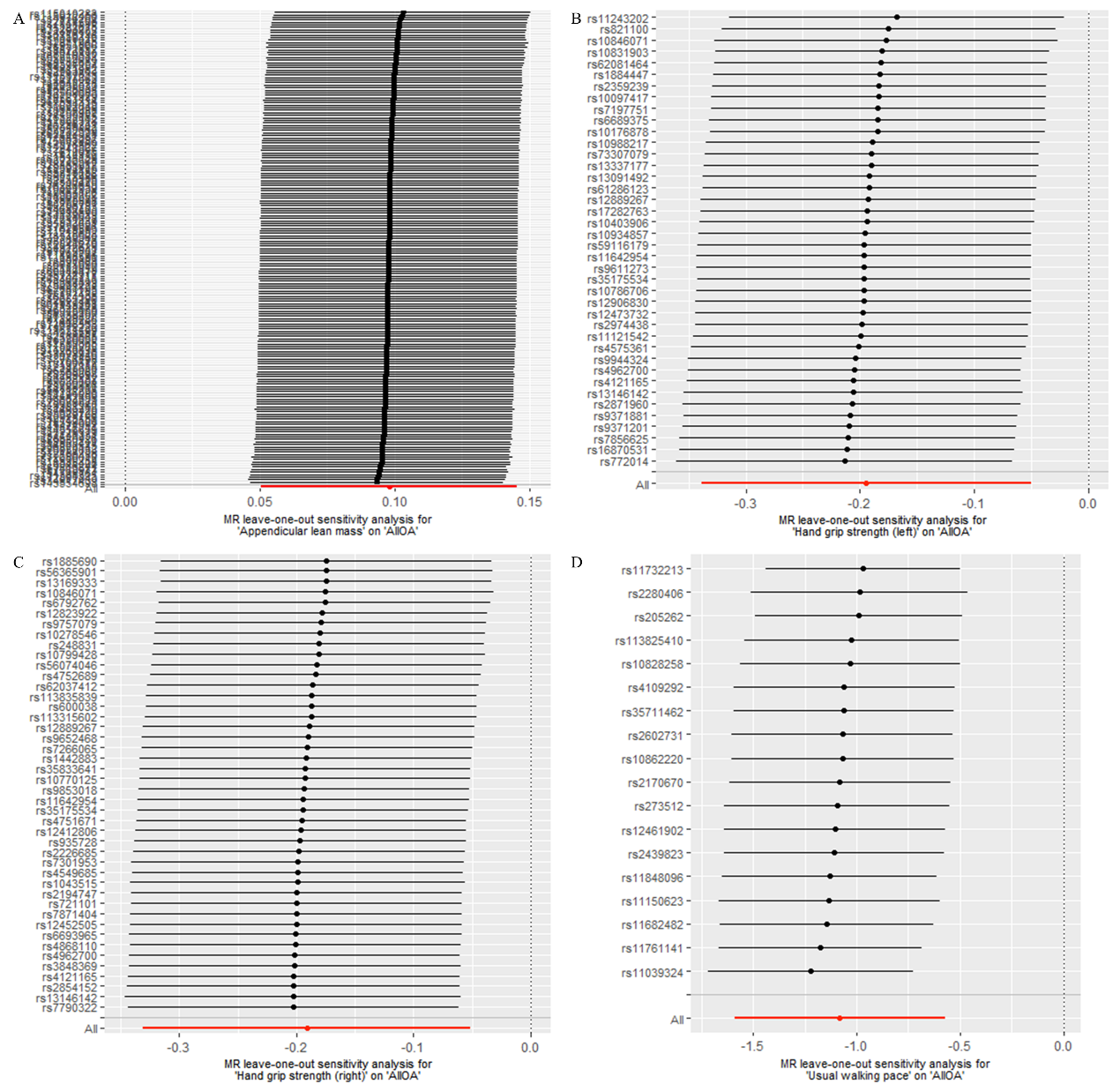


**Supplementary Fig 1. MR leave-one-out sensitivity analysis for sarcopenia related traits on All OA.**


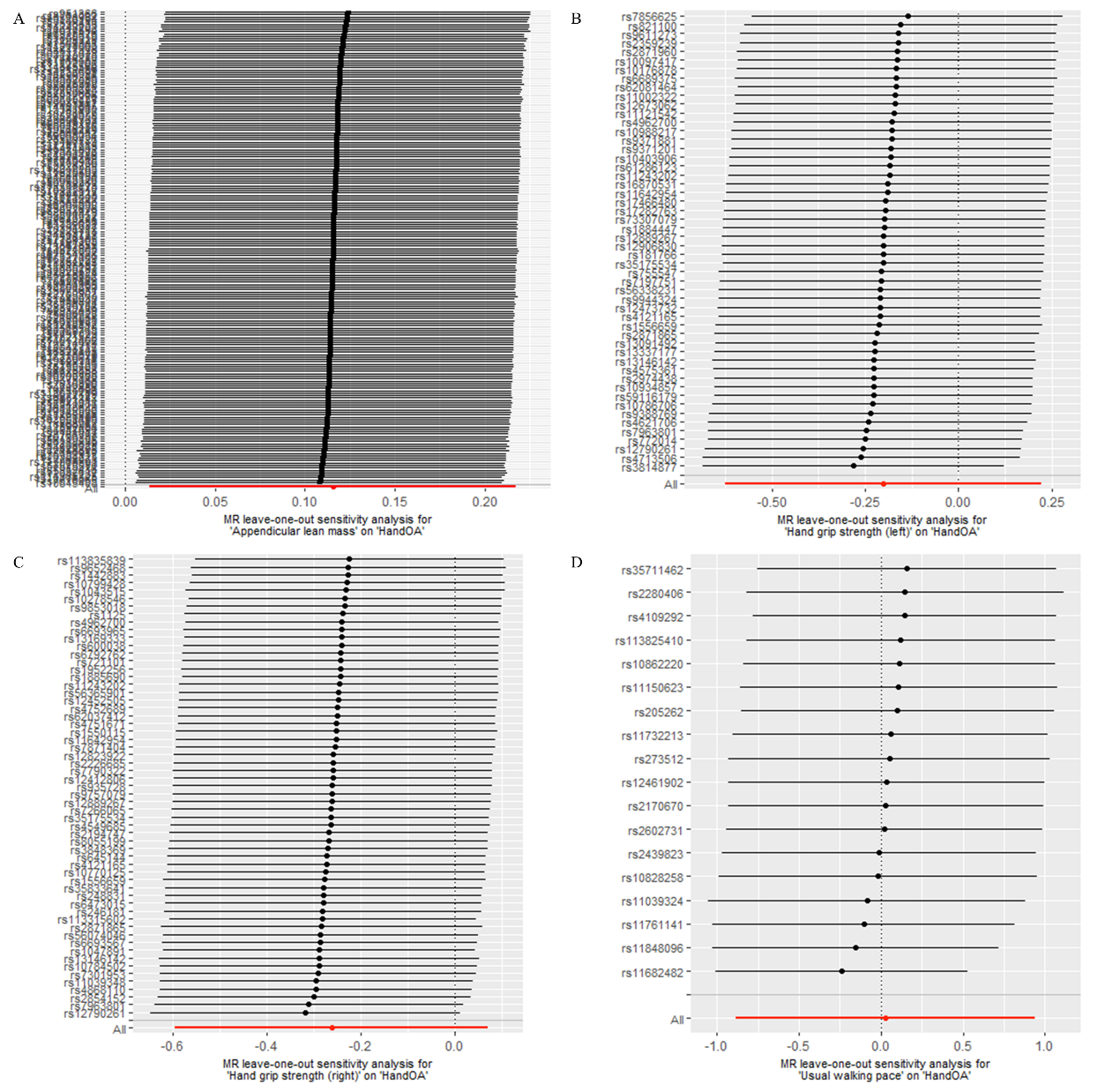


**Supplementary Fig 2. MR leave-one-out sensitivity analysis for sarcopenia related traits on Hand OA.**


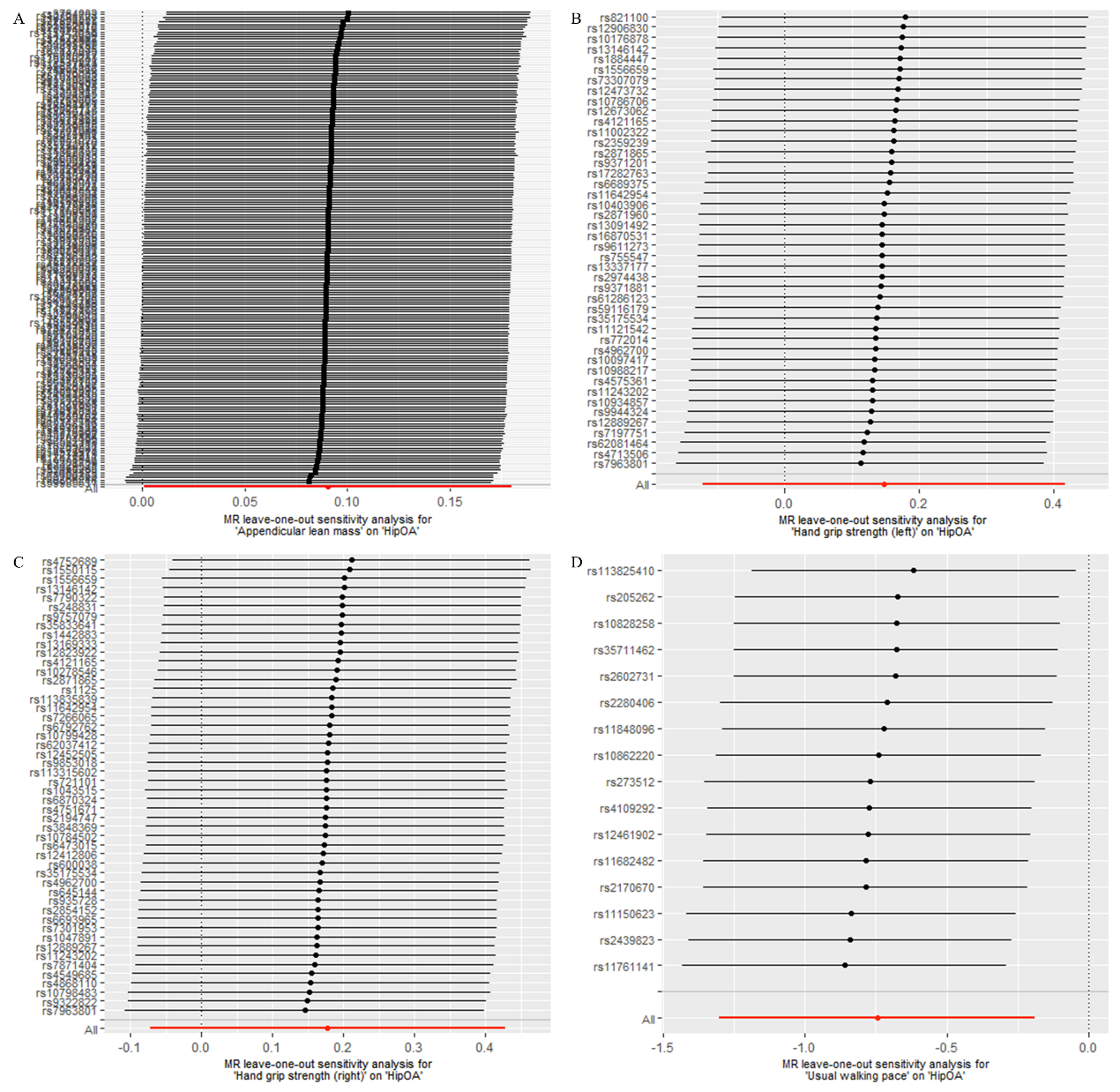


**Supplementary Fig 3. MR leave-one-out sensitivity analysis for sarcopenia related traits on Hip OA.**


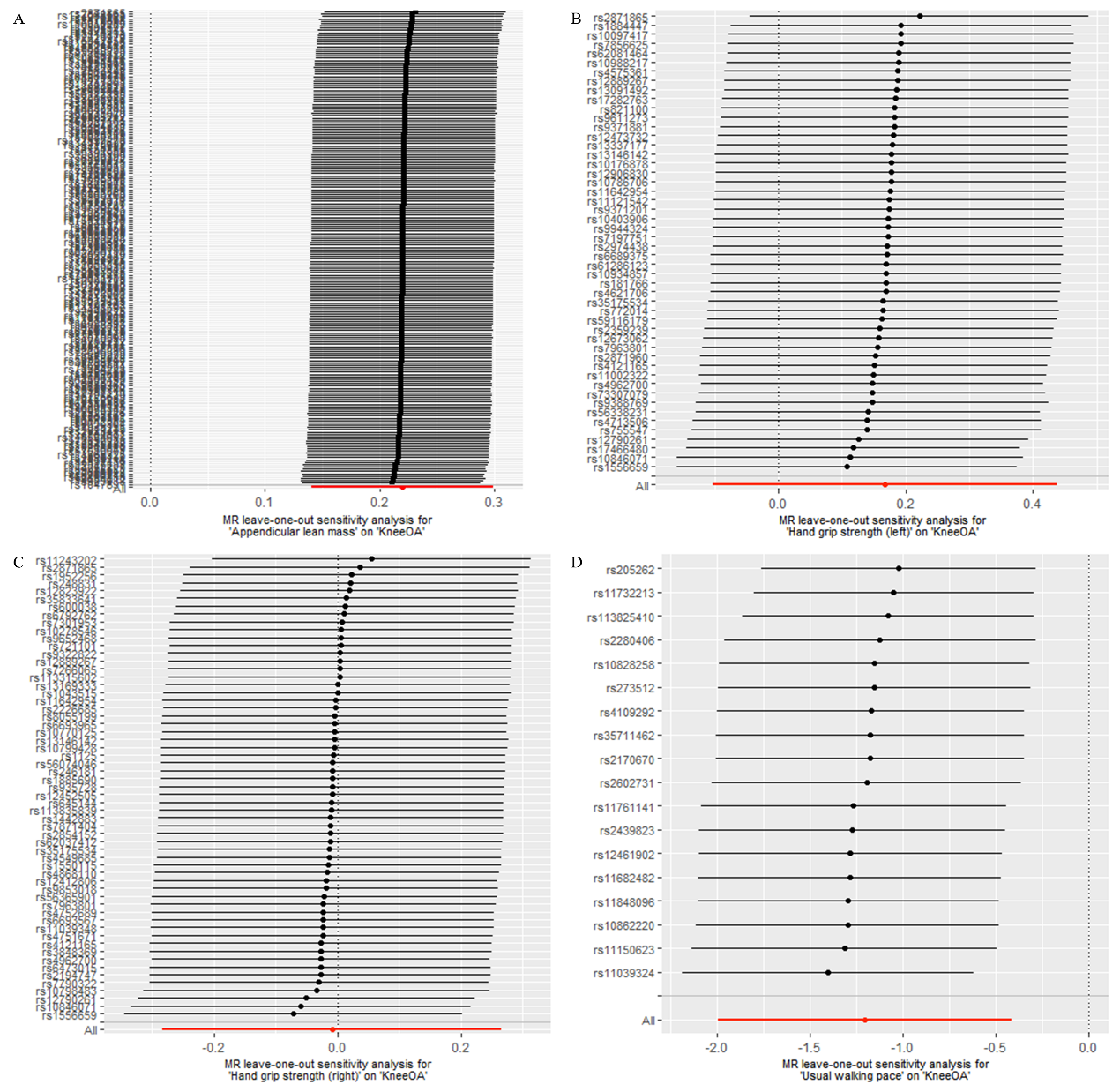


**Supplementary Fig 4. MR leave-one-out sensitivity analysis for sarcopenia related traits on Knee OA.**


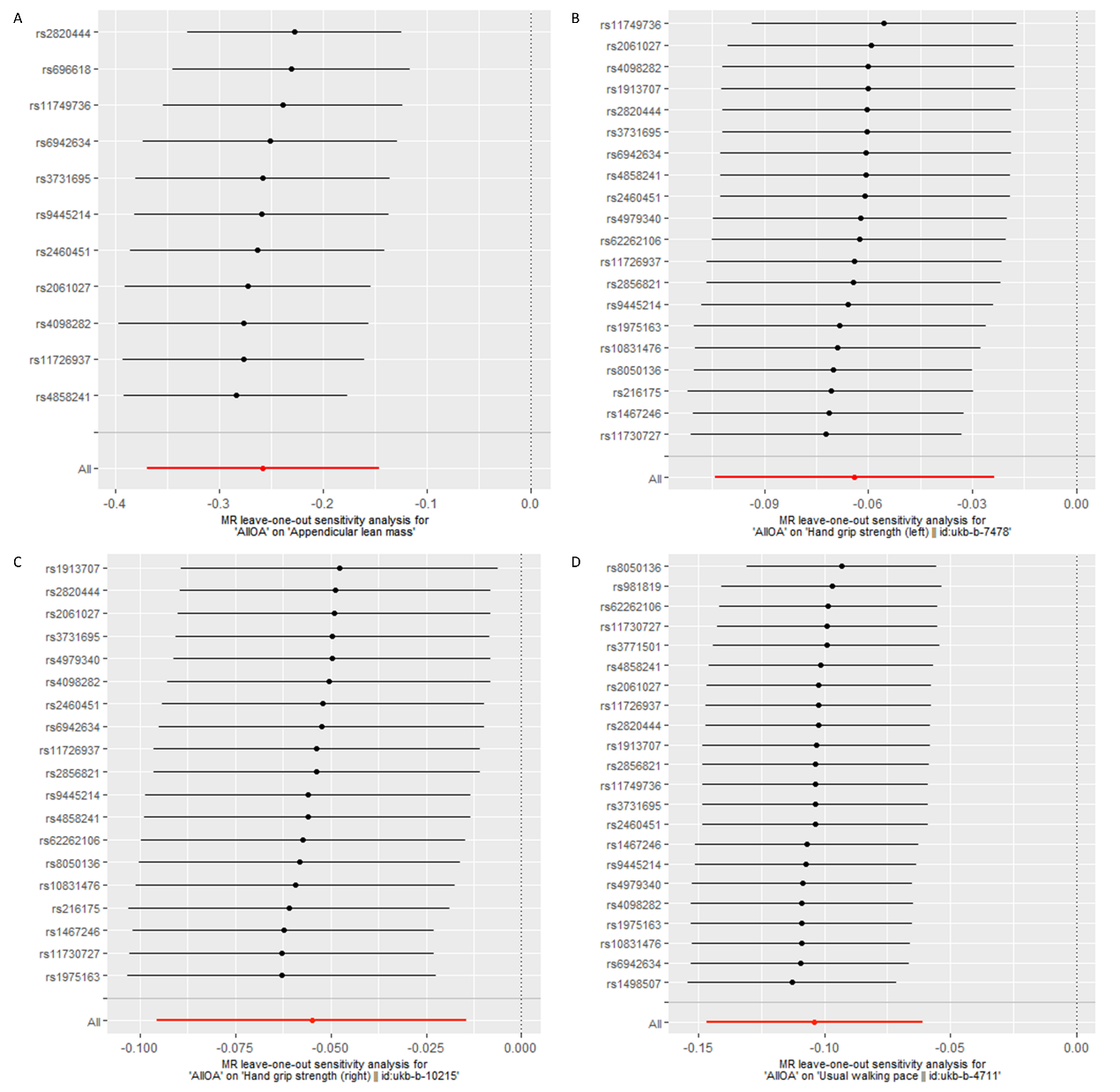


**Supplementary Fig 5. MR leave-one-out sensitivity analysis for All OA on sarcopenia related traits.**


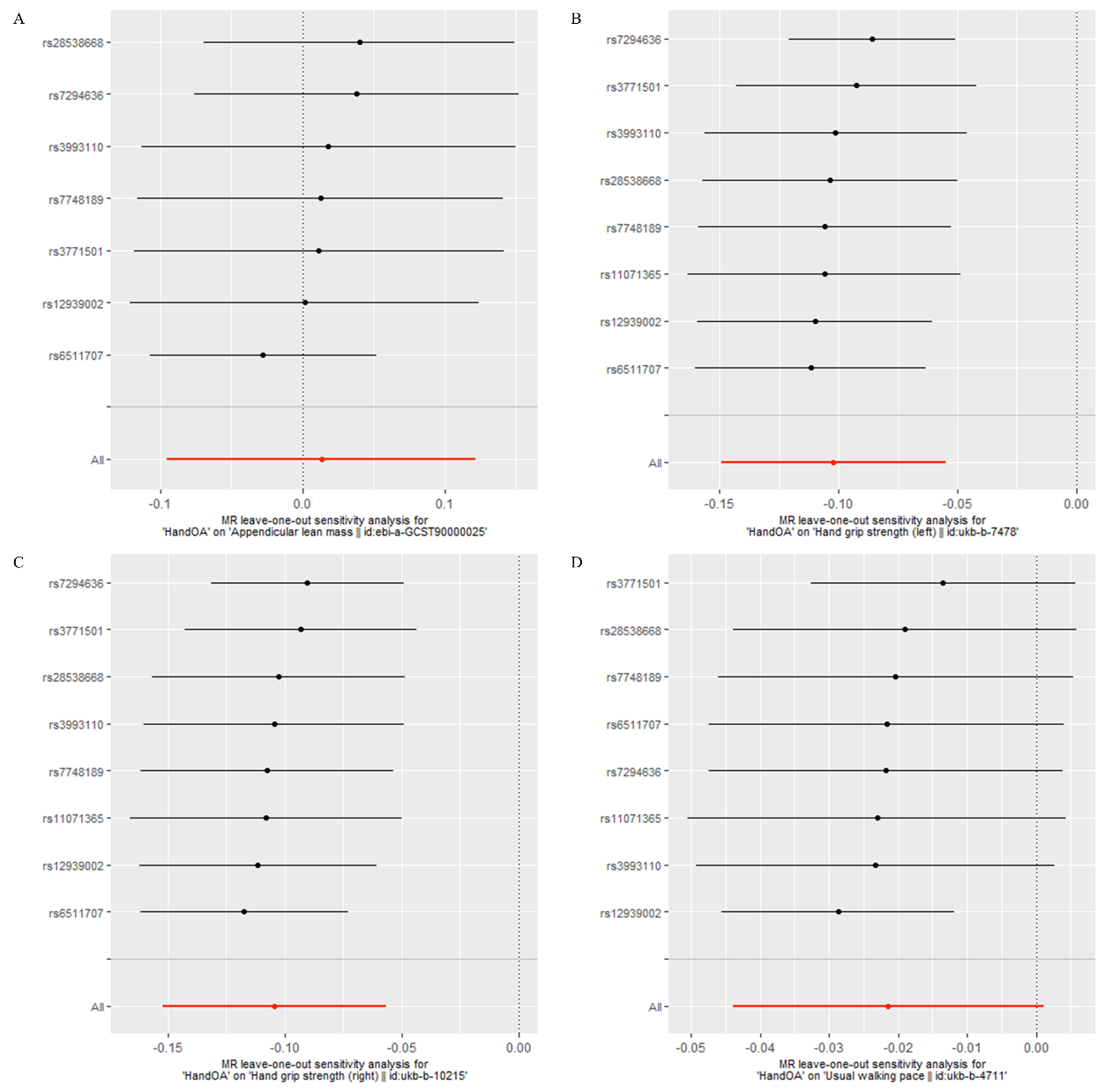


**Supplementary Fig 6. MR leave-one-out sensitivity analysis for Hand OA on sarcopenia related traits.**


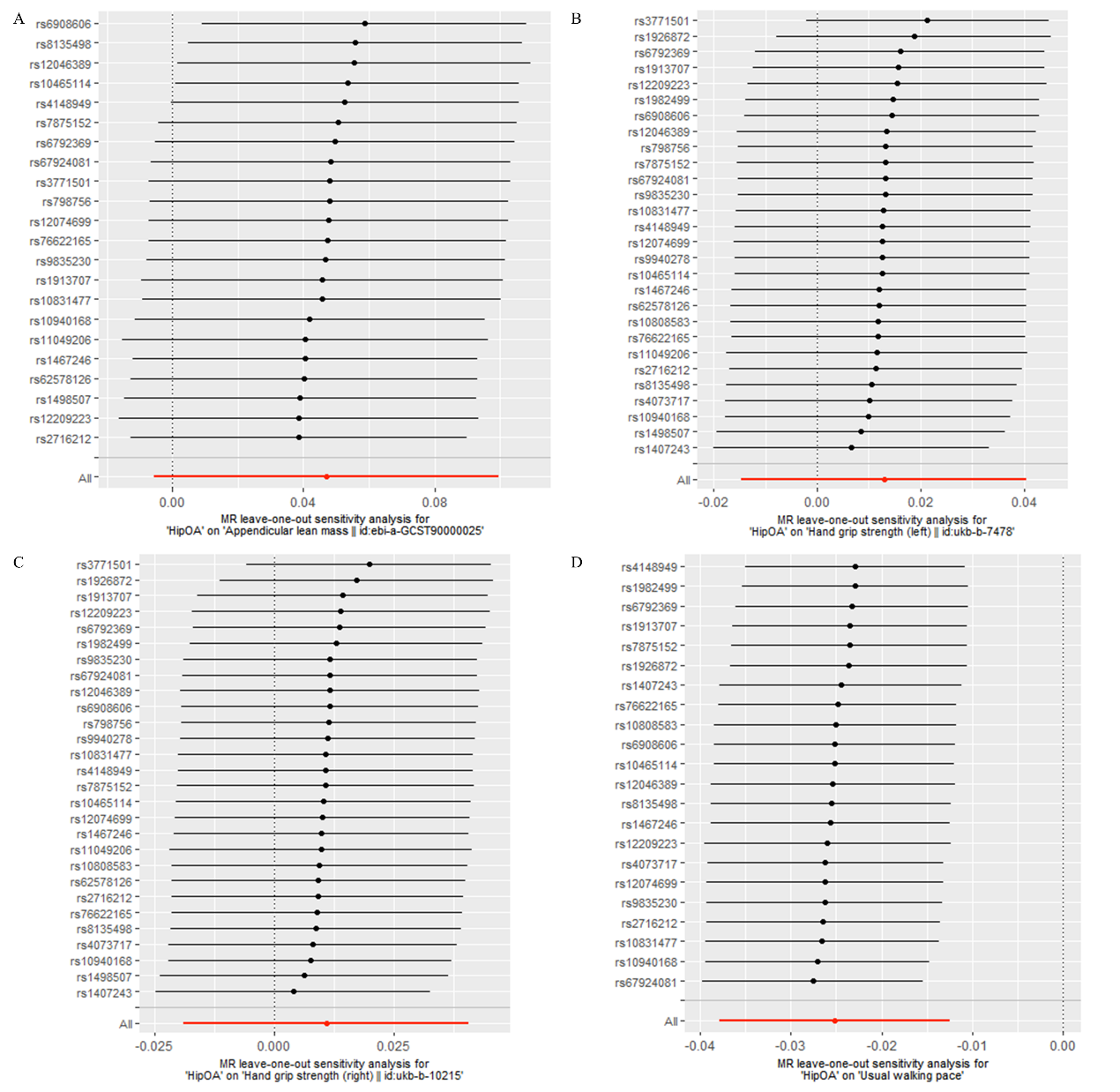


**Supplementary Fig 7. MR leave-one-out sensitivity analysis for Hip OA on sarcopenia related traits.**


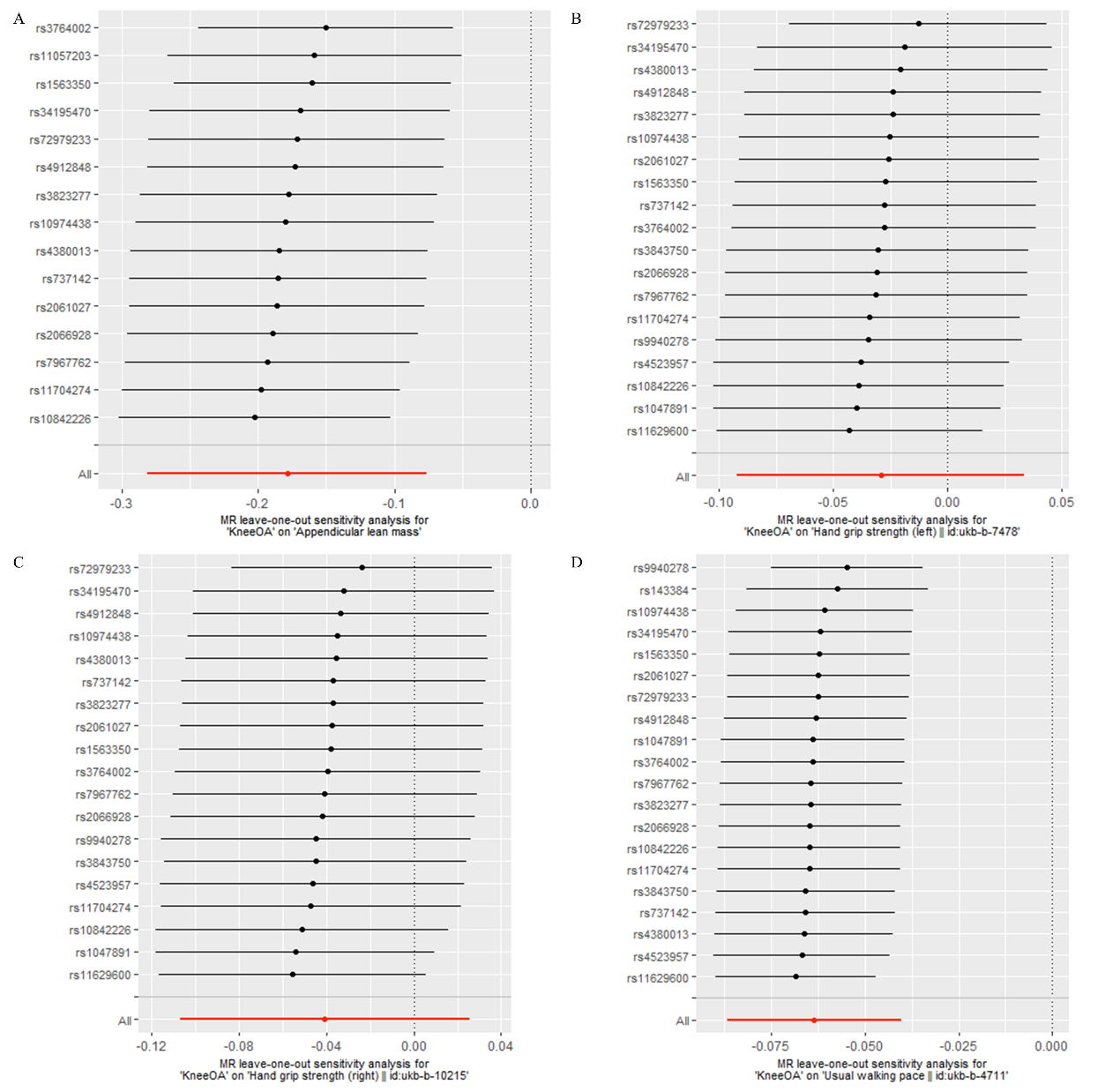


**Supplementary Fig 8. MR leave-one-out sensitivity analysis for Knee OA on sarcopenia related traits.**
